# Evolutionary dynamics of eukaryotic selenoproteomes: large selenoproteomes may associate with aquatic life and small with terrestrial life

**DOI:** 10.1186/gb-2007-8-9-r198

**Published:** 2007-09-19

**Authors:** Alexey V Lobanov, Dmitri E Fomenko, Yan Zhang, Aniruddha Sengupta, Dolph L Hatfield, Vadim N Gladyshev

**Affiliations:** 1 Department of Biochemistry, University of Nebraska, Lincoln, NE 68588, USA; 2 Section on the Molecular Biology of Selenium, National Cancer Institute, National Institutes of Health, Bethesda, MD 20892, USA

## Abstract

**Background:**

Selenocysteine (Sec) is a selenium-containing amino acid that is co-translationally inserted into nascent polypeptides by recoding UGA codons. Selenoproteins occur in both eukaryotes and prokaryotes, but the selenoprotein content of organisms (selenoproteome) is highly variable and some organisms do not utilize Sec at all.

**Results:**

We analyzed the selenoproteomes of several model eukaryotes and detected 26 and 29 selenoprotein genes in the green algae *Ostreococcus tauri *and *Ostreococcus lucimarinus*, respectively, five in the social amoebae *Dictyostelium discoideum*, three in the fly *Drosophila pseudoobscura*, and 16 in the diatom *Thalassiosira pseudonana*, including several new selenoproteins. Distinct selenoprotein patterns were verified by metabolic labeling of *O. tauri *and *D. discoideum *with ^75^Se. More than half of the selenoprotein families were shared by unicellular eukaryotes and mammals, consistent with their ancient origin. Further analyses identified massive, independent selenoprotein losses in land plants, fungi, nematodes, insects and some protists. Comparative analyses of selenoprotein-rich and -deficient organisms revealed that aquatic organisms generally have large selenoproteomes, whereas several groups of terrestrial organisms reduced their selenoproteomes through loss of selenoprotein genes and replacement of Sec with cysteine.

**Conclusion:**

Our data suggest many selenoproteins originated at the base of the eukaryotic domain and show that the environment plays an important role in selenoproteome evolution. In particular, aquatic organisms apparently retained and sometimes expanded their selenoproteomes, whereas the selenoproteomes of some terrestrial organisms were reduced or completely lost. These findings suggest a hypothesis that, with the exception of vertebrates, aquatic life supports selenium utilization, whereas terrestrial habitats lead to reduced use of this trace element due to an unknown environmental factor.

## Abstract 2

In silico and metabolic labeling studies of the selenoproteomes of several eukaryotes revealed distinct selenoprotein patterns as well as an ancient origin of selenoproteins and massive, independent losses in land plants, fungi, nematodes, insects and some protists, suggesting that the environment plays an important role in selenoproteome evolution.

## Background

Selenium is an essential trace element in many, but not all, life forms. Its essentiality is based on the fact that this element is present in natural proteins in the form of selenocysteine (Sec), a rare amino acid that chemically differs from serine or cysteine (Cys) by a single atom (for example, Se instead of O or S) [1]. Sec is known as the 21st amino acid in the genetic code as it has its own biosynthetic machinery, a tRNA and an elongation factor, and is inserted into nascent polypeptides co-translationally in response to the Sec codon, UGA [2-4]. Selenoproteins often escape attention of genome annotators, because in-frame UGA codons are interpreted as stop signals. However, several bioinformatics tools have recently been developed that help identify these genes [5,6]. The use of these methods begins to shed light on proteins and processes dependent on selenium, as well as on the occurrence and distribution of these processes in various life forms.

Sec is typically found in active sites of redox enzymes, which are functionally similar to thiol-based oxidoreductases [7]. Sec-containing proteins occur in all major lines of descent (for example, eukaryota, eubacteria and archaea), but not all organisms have these proteins. Prokaryotic genomes have been extensively analyzed for the occurrence of selenoprotein genes [8], but among eukaryotes, only the genomes of mammals (human, mouse) [9], nematodes (*Caenorhabditis elegans *and *C. briggzae*) [10], fruit fly (*Drosophila melanogaster*) [11], green alga (*Chlamydomonas reinhardtii*) [12] and Plasmodia [13,14] have been analyzed with regard to the entire set of selenoproteins (selenoproteomes). In addition, the genomes of the plant *Arabidopsis thaliana *and the yeast *Saccharomyces cerevisiae *have been scanned for the occurrence of selenoprotein genes and Sec biosynthetic/insertion machinery genes and found to have neither [9].

Selenoproteome analyses also revealed that various organisms have substantially different sets of selenoproteins. One example of uneven selenoprotein occurrence is selenoprotein U (SelU), which occurs in fish, birds and some unicellular eukaryotes, but is present in the form of a Cys-containing homolog in mammals and many other eukaryotes. Even a narrower occurrence has been described for SelJ and Fep15 [15,16].

In this study, we characterized the selenoproteomes encoded in several completely sequenced eukaryotic genomes. Detailed analyses of these selenoproteomes and comparison with those of other eukaryotic model organisms revealed an ancient origin of most eukaryotic selenoproteins and a possibility of increased Sec utilization in aquatic environments and decreased use of Sec in terrestrial habitats. These studies provide important insights into selenoprotein origin and dynamics of selenoprotein evolution.

## Results and discussion

### Eukaryotic selenoproteomes

Several eukaryotes have been previously analyzed for their selenoprotein content (selenoproteomes). These studies identified 24-25 selenoproteins in mammals and 0-4 selenoproteins in other organisms. It is generally thought that many eukaryotic selenoproteins evolved in vertebrates, but evolutionary paths have not been examined for the majority of these proteins. In this work, we analyzed the selenoproteomes of several additional model eukaryotes, whose genomes have been completed. These included marine algae (*Ostreococcus tauri *and *O. lucimarinus*), a diatom (*Thalassiosira pseudonana*), a soil amoeba (*Dictyostelium discoideum*), an insect (*Drosophila pseudoobscura*), and a red alga (*Cyanidioschyzon merolae*).

#### Drosophila pseudoobscura

The *D. pseudoobscura *subgroup [17] is found mainly in the temperate and tropical zones of the New World [18]. Application of an earlier version of SECISearch to the *D. melanogaster *genome identified three selenoprotein genes (SelK/G-rich, SelH/BthD and SPS2); however, it was not known whether this set represents the entire *Drosophila *selenoproteome. We applied an advanced version of SECISearch (see Materials and methods and Additional data file 1) to analyze the *D. pseudoobscura *genome and, in addition, analyzed *D. pseudoobscura *and *D. melanogaster *genomes in parallel to identify evolutionarily conserved selenocysteine insertion sequence (SECIS) elements using relaxed SECIS criteria. These searches resulted in the same, already known set of three selenoproteins (Table 1), suggesting that the selenoproteome of insects of the *Drosophila *genus consists of these three proteins. By homology analyses, we then identified three selenoproteins in a mosquito, *Anopheles gambiae*, and one in a honey bee, *Apis mellifera*.

**Table 1:** Identification of selenoprotein genes in eukaryotic model organisms

		Loose pattern		Default pattern		
				
Organism name	Genome, thousands of bp	Primary sequence criteria	Energy criteria	Primary sequence criteria	Energy criteria	Number of selenoproteins
*O. lucimarinus*	13,393	31,132	7,541	2,120	464	29
*O. tauri*	16,414	30,381	7,379	1,934	401	26
*T. pseudonana*	32,577	81,040	8,977	3,129	675	16
*D. discoideum*	34,564	37,435	7,11	2,128	37	5
*D. pseudoobscura*	138,581	181,793	20,702	6,303	1,010	3
*C. merolae*	16,381	27,578	5,987	651	149	0

#### Ostreococcus tauri

*O. tauri *is a unicellular green alga that was discovered in the Mediterranean Thau lagoon in 1994. It belongs to the family Prasinophyceae, which is thought to be the most primitive in the green plant lineage from which all other green algae and ancestors of land plants have descended. This organism has a very small genome, 11.5 Mb [19], especially when compared to other sequenced Plantae genomes (for example, the *Arabidopsis *genome is 125 Mb [20] and that of *Chlamydomonas *exceeds 100 Mb [21,22]). The *O. tauri *genome is densely packed and provides a useful genomic model for green plants [23]. Previous research revealed the lack of selenoproteins in land plants [9], whereas 10 selenoproteins were detected in the green alga *C. reinhardtii *[12]. Surprisingly, we detected 26 selenoprotein genes in *O. tauri*.

Among the known selenoproteins detected in *O. tauri*, fourteen were homologs of human selenoproteins (thioredoxin reductase (TR), SelT, SelM, SelK, SelS, Sep15, SelO, SelH, SelW and five glutathione peroxidase (GPx) homologs), five were homologs of eukaryotic selenoproteins with restricted distribution (MsrA, SelU and three PDI homologs) and three were homologs of bacterial selenoproteins (methyltransferase, thioredoxin-fold protein and peroxiredoxin). We also identified four novel eukaryotic selenoproteins in the *O. tauri *genome. These included a predicted membrane selenoprotein (MSP) and three hypothetical proteins of unknown function. In addition, several excellent SECIS element candidates were identified during analysis, but at present no suitable open reading frames (ORFs) could be identified upstream of these structures, in part because of the inadequate length of contigs. Therefore, the total number of *Ostreococcus *selenoproteins might be even higher than 26.

**Figure 1:**
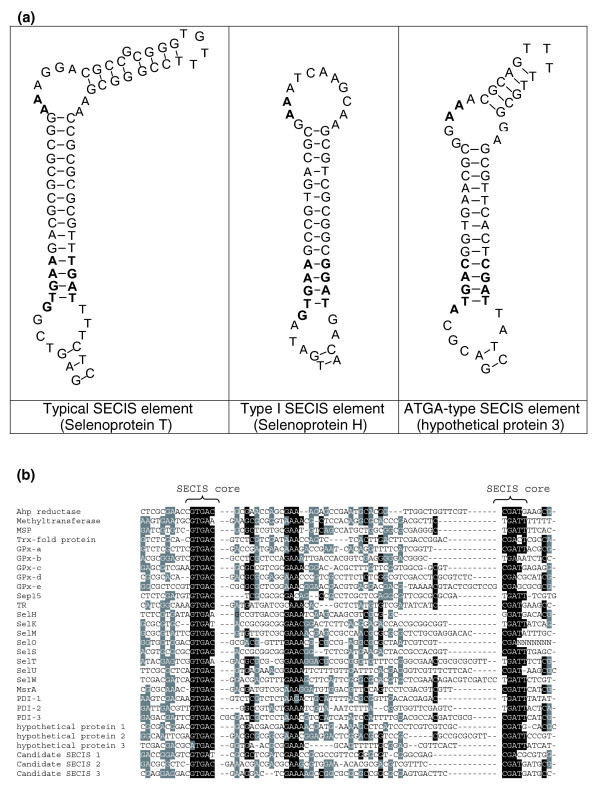
*Ostreococcus *SECIS elements. **(a) **The most characteristic features of *O. tauri *and *O. lucimarinus *SECIS elements are a long mini-stem and an unpaired G preceding the SECIS quartet (core). A SelT SECIS element is shown as a typical example (left structure). Only two exceptions were found, including a type I SECIS element in SelH (middle structure) and a SECIS element with an unpaired A nucleotide preceding the SECIS core (right structure). **(b) **Alignment of nucleotide sequences of all *O. tauri *SECIS elements. Location of the SECIS core is indicated. Conserved nucleotides are highlighted. Black and grey highlighting shows sequence conservation.

Of interest was the observation that all *O. tauri *SECIS elements except one had a conserved G in the position directly preceding the quartet of non-Watson-Crick interacting nucleotides (Figure 1). Most eukaryotic SECIS elements have an A in this position, although the G was described in several zebrafish and nematode selenoprotein genes [10,24,25]. In addition, almost all *O. tauri *SECIS elements had a long mini-stem in the apical portion of the structure (for example, SelT in Figure 1). This feature was also observed previously in a number of *Chlamydomonas *SECIS elements [12].

**Figure 2:**
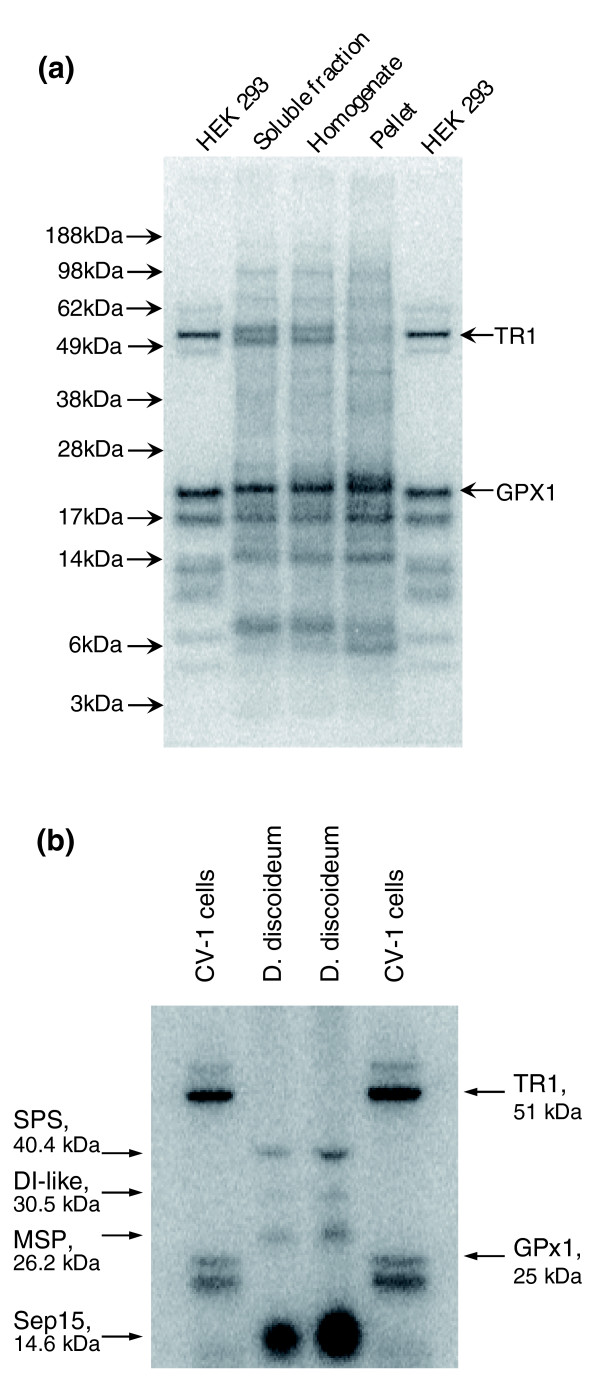
Metabolic labeling of *O. tauri *and *D. discoideum *with ^75^Se. *O. tauri *and *D. discoideum *cells were grown in the presence of ^75^Se [selenite], cell lysates prepared, proteins resolved by SDS-PAGE and analyzed using a PhosphorImager. **(a) ***O. tauri*. Three middle lanes represent the soluble fraction, homogenate and pellet fraction as shown above the gel. For comparison, HEK 293 cells were metabolically labeled with ^75^Se, and migrations of thioredoxin reductase 1 (TR1) and glutathione peroxidase 1 (GPx1) are shown. **(b) ***D. discoideum*. Two middle lanes represent two independent samples of ^75^Se-labeled *D. discoideum *cells. The four radioactive bands correspond to the indicated selenoproteins identified *in silico*. For comparison, monkey CV-1 cells were metabolically labeled with ^75^Se, and migrations of TR1 and GPx1 are shown on the right.

We metabolically labeled *O. tauri *cells with ^75^Se and analyzed the selenoprotein pattern on SDS PAGE gels using a PhosphorImager (Figure 2a). This method detects the most abundant selenoproteins. The overall pattern was similar to that of human HEK 293 and other mammalian cells. As in mammalian cells, the dominant 25 kDa band in the alga was likely a glutathione peroxidase, and one or both major selenoprotein bands in the 50-55 kDa range likely corresponded to thioredoxin reductase. Consistent with the genomics analysis, the number of selenoprotein bands in the *O. tauri *sample was higher than in mammalian cells.

#### Ostreococcus lucimarinus

*O. lucimarinus*, previously known as *Ostreococcus *sp. CCE9901, is a close relative of *O. tauri *adapted to high light and isolated from surface waters. Its genome size is 13.2 Mb. Homologs of all identified *O. tauri *selenoproteins were found in *O. lucimarinus*. In addition, three new sequences were identified, raising the number of selenoproteins in this organism to 29. This is the largest selenoproteome of all previously analyzed eukaryotes (although even larger selenoproteomes apparently exist; Lobanov and Gladyshev, unpublished). Additional selenoproteins included a peroxiredoxin, and peroxiredoxin-like and SelW-like proteins. The latter *O. lucimarinus *selenoprotein contained two predicted Sec residues.

Similar to *O. tauri*, all *O. lucimarinus *SECIS elements except one had a conserved G in the position directly preceding the SECIS core (Figure 1a), and in addition a single ATGA-type SECIS element was found. Interestingly, single ATGA-type SECIS elements occur in different selenoprotein genes in the two *Ostreococcus *species. In *O. lucimarinus*, this SECIS type is within a glutathione peroxidase gene, while in *O. tauri *the ATGA-type SECIS is in the gene for a hypothetical protein. In contrast to *O. tauri*, no type I SECIS elements (Figure 1a) were found in *O. lucimarinus*.

#### Cyanidioschyzon merolae

*C. merolae *is an ultrasmall unicellular red alga that lives in acidic hot springs. It is thought to retain primitive features of cellular and genome organization. *C. merolae *has a simple cell architecture, containing a single nucleus, a single mitochondrion and a single chloroplast. Its genome size is 16 Mbp, which is approximately one-seventh the size of the *A. thaliana *genome. Its chloroplast might be among the most ancestral [26]. A BLAST search against the *C. merolae *genome revealed several known components of the Sec insertion machinery, including SBP2, EFsec, SecS and SPS2, suggesting that selenoproteins should also be present in this organism. However, a search for SECIS elements followed by ORF analyses revealed no candidate selenoproteins in the *C. merolae *genome.

**Figure 3:**
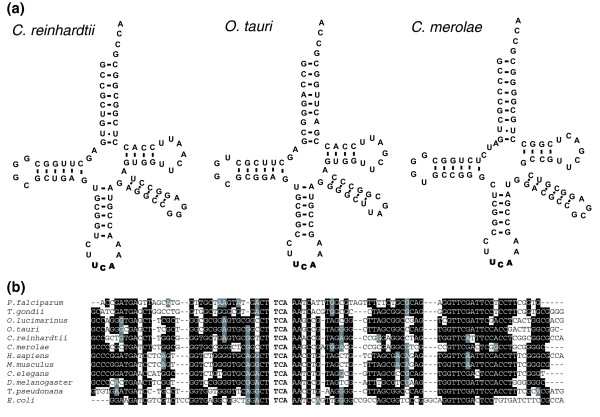
Sec tRNA. **(a) **Cloverleaf structures of Sec tRNAs from *C. reinhardtii, O. tauri *and *C. merolae*. **(b) **Nucleotide sequence alignment of *C. reinhardtii *and *C. merolae *Sec tRNAs with known Sec tRNAs. Black and grey highlighting shows sequence conservation.

A BLASTN-based analysis of the *C. merolae *genome using known Sec tRNAs as query sequences did not identify Sec tRNA homologs, and the searches that utilized default versions of standard tRNA detection programs, ARAGORN and tRNAscan-SE, were also unsuccessful. We were able to identify the *C. merolae *Sec tRNA using our recently described tool for detection of unusual tRNAs [27]. This tRNA (Figure 3) has all the features characteristic of Sec tRNAs, such as the UCA anticodon and a long variable stem.

**Figure 4:**
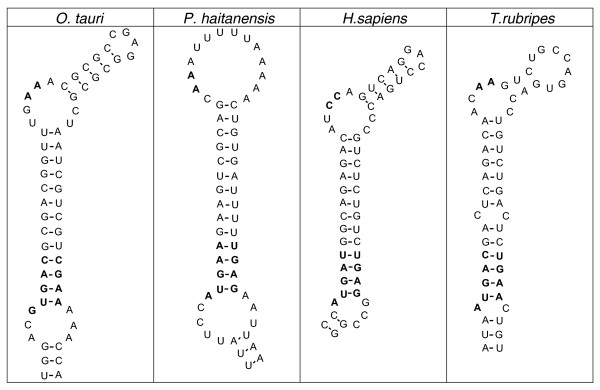
Red algae selenoprotein O. SECIS elements in *O. tauri *(green alga) and *P. haitanensis *(red alga) SelO genes. The *P. haitanensis *SECIS element belongs to type I, while *O. tauri *to type II structures.

We applied additional sensitive tools for identification of selenoproteins in the red algal genome. Most homologs of known selenoproteins were found to either have Cys in place of Sec or were missing in this organism. We further carried out a search for Sec/Cys pairs in homologous sequences using the *C. merolae *genome and all protein sequences extracted from NCBI non-redundant database. Again, no selenoproteins were detected in *C. merolae*. To test if related organisms possess selenoproteins, all available red algal ESTs were extracted from NCBI dbEST and searched for SECIS elements using SECISearch. This analysis revealed one *bona-fide *selenoprotein, SelO, in *Porphyra haitanensis*, which was also highly homologous to the *O. tauri *SelO (Additional data file 2). The red algal SECIS element was also detected in these sequences (Figure 4).

The presence of the Sec insertion machinery in *C. merolae *and detection of a selenoprotein in a related red alga suggest that Sec-containing proteins exist in this evolutionary branch. It is possible that the difficulties in identifying selenoproteins in *C. merolae *may be due to incompleteness of the genome or presence of lineage-specific selenoprotein(s), whose homologs are not represented in sequence databases. In addition, it is possible that the small selenoproteome of *C. merolae *resulted in unusual SECIS elements, which could not be detected by SECISearch. It is clear, however, that the selenoproteome of this organism is extremely small.

#### Thalassiosira pseudonana

*T. pseudonana *is a marine-centric diatom that serves as a model for studies on diatom physiology [28]. A Sec tRNA sequence [29] and one selenoprotein, Sec-containing glutathione peroxidase [30], have been identified in this organism. In this work, we isolated and directly sequenced the *T. pseudonana *Sec tRNA (see Additional data file 3 for the sequence and clover-leaf structure), which exhibited features typical of eukaryotic Sec tRNAs.

By searching for SECIS elements, we detected 16 selenoprotein genes in *T. pseudonana *(Table 1). In addition, a partial SelO sequence was detected, but it did not include the regions corresponding to the possible Sec codon and SECIS element. The *T. pseudonana *selenoproteome includes two GPx homologs, SelT, TR, SPS2, two SelM, two SelU, MsrA, two PDI homologs, a predicted SAM-dependent methyltransferase, two peroxiredoxins and one thioredoxin-like protein. It is remarkable that in spite of large evolutionary distances, *Ostreococcus*, *Thalassiosira *and mammalian selenoprotein sets were large and showed a significant overlap, whereas many other eukaryotes, including some animals, had small selenoproteomes.

#### Dictyostelium discoideum

*D. discoideum *is a slime mold that primarily inhabits soil or dung and feeds on bacteria. We previously reported the finding of Sec tRNA in this organism [31]. In the present study, we analyzed its selenoproteome and found SPS2, SelK, Sep15, MSP and a homolog of thyroid hormone deiodinase (Table 2). The presence of the deiodinase homolog was unexpected as thyroid hormones are not known to occur in amoebae. However, this sequence assignment was unambiguous; for example, the *D. discoideum *selenoprotein exhibited 39% sequence identity to iodothyronine deiodinase type I from *Fundulus heteroclitus *(accession number AAO31952) and 37% identity to iodothyronine deiodinase type III from *Sus scrofa *(accession number NP_001001625). Among the five amoebae selenoproteins, MSP had the narrowest distribution and could only be detected in *Dictyostelium*, *Chlamydomonas*, *Volvox *and both *Ostreococcus *species. This novel selenoprotein had two Sec residues.

**Table 2:** Selenoproteins identified in the analyzed eukaryotic genomes

Selenoprotein family	*O. tauri*	*O. lucimarinus*	*T. pseudonana*	*D. discoideum*	*D. pseudoobscura*
SelK	+	+		+	+
SelH	+	+			+
SPS2			+	+	+
DI				+	
Sep15	+	+		+	
MSP	+	+		+	
Gpx	+++++	+++++	++		
SelT	+	+	+		
TR	+	+	+		
SelM	+	+	++		
SelU	+	+	++		
MsrA	+	+	+		
PDI	+++	+++	++		
Methyltransferase	+	+	+		
Peroxiredoxin	+	+++	++		
Thioredoxin-fold protein	+	+	+		
SelO	+	+			
SelW	+	++			
SelS	+	+			
Hypothetical protein 1	+	+			
Hypothetical protein 2	+	+			
Hypothetical protein 3	+	+			

Total	26	29	16	5	3

Each '+' corresponds to one selenoprotein gene.

**Figure 5:**
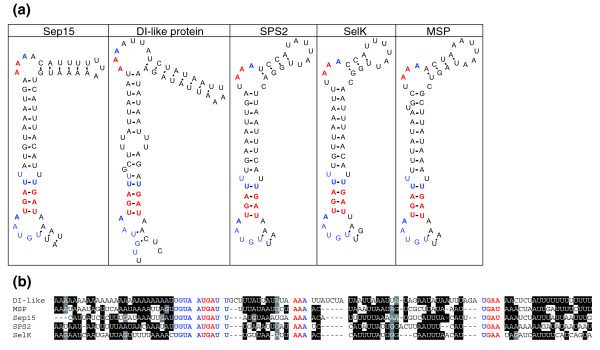
*Dictyostelium discoideum *SECIS elements. **(a) **SECIS elements in *D. discoideum *selenoprotein genes. Sequences conserved in eukaryotic SECIS elements are shown in red, and *Dictyostelium*-specific conserved sequences are shown in blue. **(b) **Alignment of *D. discoideum *SECIS elements. A UGUA sequence preceding the SECIS core, and a U-U mismatch in the stem-loop structure represent additional conserved features in *Dictyostelium *SECIS elements. Black and grey highlighting shows sequence conservation.

Interestingly, all identified *Dictyostelium *SECIS elements had a highly conserved UGUA sequence that preceded the SECIS core, and a U-U mismatch immediately following it (Figure 5). The SECIS element of the deiodinase-like protein had two U-U mismatches; however, they were located further from the SECIS core. All detected SECIS elements were type II structures [24]. The deiodinase-like SECIS element had an extremely long mini-stem. As discussed above, the latter feature was also observed in many *Ostreococcus *selenoprotein genes, whereas it rarely occurs in SECIS structures in other organisms. All *Dictyostelium *SECIS elements had an unpaired AAA in the apical bulge. The areas of strong conservation include an SBP2-binding site and nucleotides interacting with this protein [32]. Since the five selenoproteins have different evolutionary histories and are not homologous with each other, the conservation of primary sequences in *Dictyostelium *SECIS elements must represent convergent evolutionary events.

We used the observation of unusually high sequence conservation of *Dictyostelium *SECIS elements to develop a modified version of SECISearch, which allowed the searches wherein other search parameters were relaxed. However, application of this procedure did not detect additional selenoproteins.

To further examine the *Dictyostelium *selenoproteome, we metabolically labeled the amoebae cells with ^75^Se and analyzed the selenoprotein pattern on SDS PAGE using a PhosphorImager (Figure 2b). Four selenoprotein bands were detected, which corresponded in size to the four selenoproteins identified computationally (SPS, MSP, DI and Sep15). Apparently, Sep15 was a major selenoprotein in *D. discoideum*, whereas SelK was not detected. The latter selenoprotein might be expressed at low levels or under different growth or developmental conditions than those examined in our study.

### Comparative analysis of eukaryotic selenoproteomes

Selenoproteins are found in all three domains of life, which share several protein and RNA components involved in Sec biosynthesis and insertion, suggesting an origin of the Sec machinery that predates the last universal common ancestor. Thus, Sec decoding is an ancient trait that has been maintained for hundreds of million of years without widespread expansion or loss.

**Figure 6:**
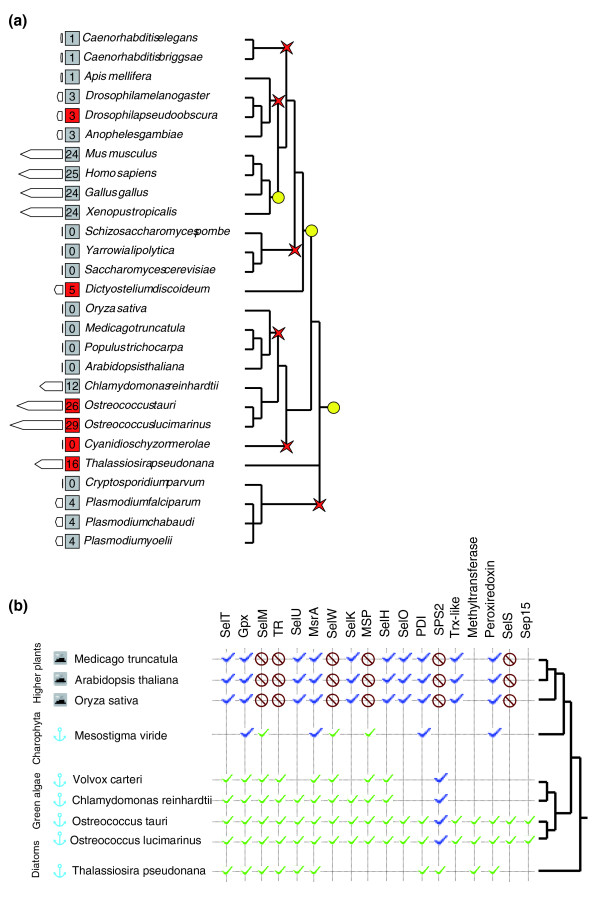
Eukaryotic selenoproteomes. **(a) **A simplified cladogram of model organisms discussed in the text that illustrates distribution of selenoproteins in eukaryotes. The number of selenoproteins in each indicated model organism is shown in red (current study) and gray (previously analyzed and other model organisms) squares, and is proportional to the size of the bars on the left. Yellow circles show possible origins of various selenoprotein families, and red crosses examples of massive selenoprotein loss. **(b) **Selenoprotein evolution in plants. The 'mountain' symbols show terrestrial organisms, and 'anchors' those that live in aquatic environments. Green checkmarks indicate the presence of an indicated selenoprotein in the corresponding genome. The presence of Cys-containing homologs is shown by blue checkmarks. Crossed red circles indicate absence of either Sec- or Cys-containing homologs. Unfilled spots correspond to lack of data due to unfinished genomes, unclear relationship between proteins and lineage specific gene duplications.

We compiled newly and previously characterized selenoproteomes and analyzed the occurrence of particular selenoproteins against taxonomic distribution of species based on the tree of life [33]. The number of selenoproteins varied from zero (in plants, yeast and some protists) to 29 (in *Ostreococcus*) (Figure 6a). Significant differences in the composition of selenoproteomes could be seen even among related organisms. For example, among viridiplantae, all higher plants lacked selenoproteins, whereas the green algae *Chlamydomonas *and *Ostreococcus *had 12 and 26-29 selenoproteins, respectively (Figure 6b). Three selenoproteins were found in *Mesostigma viride*, a Streptophyte and a common ancestor of land plants [34].

Tracing individual selenoproteins, we found that some selenoprotein families were present in many organisms and others in only a few species, yet each identified family had a unique pattern of occurrence (Figure 6a). None of the selenoproteins matched the overall Sec trait (compared to the occurrence of Sec machinery). SelK was among the most widespread selenoproteins. This protein of unknown function is present in nearly all eukaryotes that utilize Sec (but is replaced with a Cys-containing homolog in nematodes and several other organisms). An additional widespread selenoprotein was SelW, which also occurs in most (but not all) selenoprotein-containing eukaryotes. Several other selenoproteins, such as glutathione peroxidase and thioredoxin reductase, also had a wide distribution.

### Origin of many selenoproteins precedes animal evolution

Since mammalian selenoproteomes were large and included essentially all known eukaryotic selenoproteins, they were initially thought to represent the entire eukaryotic selenoproteome. Subsequent identification of selenoproteins with highly restricted occurrence added further complexity, but did not challenge the overall idea of recent evolution of the majority of eukaryotic selenoproteins. However, our analysis of selenoproteomes of six eukaryotic model organisms and their comparison with the previously characterized selenoproteomes revealed that 20 of the 25 human selenoproteins have Sec-containing homologs in many unicellular organisms. Similarly, taking into account protein families, at least 11 of the 16 mammalian selenoprotein families could be traced back to single-cell eukaryotes. SelU, which is not a selenoprotein in mammals, is present in some animals and protozoa and may be viewed as an additional ancient selenoprotein family. Overall, these data suggest that the origin of many selenoproteins not only precedes animal evolution, but can be dated back to the ancestral eukaryotes. Thus, many of these original selenoproteins were preserved during evolution and remain in vertebrates (including mammals), green algae and a variety of protists, whereas many other organisms manifested massive selenoprotein losses.

It should be noted that Cys/Sec replacement is not always unidirectional and that prior evolutionary analyses suggest that both a Sec loss and gain is possible [35]. However, the probability of independent parallel Sec gain, as well as consecutive homoplastic Sec-to-Cys and Cys-to-Sec substitutions in a single protein position, is extremely rare, and no selenoprotein families are known that evolved more than once. Two factors are required for a Cys-to-Sec change to take place. First, the presence of Sec insertion machinery, such as Sec tRNA, SECIS-binding protein SBP2, Sec-specific elongation factor and Sec synthase. This requirement is met (for example, all components of the machinery are present) if at least one other selenoprotein is present in the same organism. Second, a SECIS element should evolve in the 3'-untranslated region. While only a single nucleotide change is sufficient to change the codon from Cys to Sec (that is, UGA instead of UGC or UGU), evolution of new SECIS elements is difficult. On the other hand, once Sec is replaced with Cys, the presence of the SECIS element provides no competitive advantage and this structure is quickly lost. Unless the reverse Cys-to-Sec mutation takes place before disruption of the SECIS element, the probability of restoring Sec is extremely low. Unless strong pressure exists to preserve Sec, its functional replacement with Cys may be expected. Combined, these factors allow us to assume that the character-state Sec follows Dollo's behavior.

### Selenoproteins with restricted occurrence are common to organisms with large selenoproteomes

In addition to the many ancient eukaryotic selenoproteins, several selenoproteins have a more narrow distribution. For example, SelP, SelN, MsrB and SelI appear to be specific to animals, whereas MSP, peroxiredoxin and thioredoxin-like protein could be detected only in unicellular eukaryotes. These observations suggest an emerging picture of selenoprotein evolution wherein core selenoprotein families evolved first, followed by the origin of additional selenoproteins in more narrow groups of organisms. The new selenoproteins further increased the size of the selenoproteomes and remain prevalent in organisms with large selenoproteomes. In our current analysis, several *Ostreococcus *and *Thalassiosira *selenoproteins fit this pattern, in addition to the rare selenoproteins previously discovered (for example, SelU, SelJ and Fep15). However, it could not be excluded that new selenoproteins might also occasionally evolve in organisms with small selenoproteomes (for example, red algae).

### Independent events of massive selenoprotein loss in eukaryotes

We further identified and examined several groups of organisms characterized by massive selenoprotein loss. Location of these organisms on the eukaryotic tree of life suggests independent events of selenoprotein loss (Figure 6a). Five examples of selenoprotein loss are discussed below.

#### Plants

As discussed above, *A. thaliana*, *O. sativa *and other higher plants lost both selenoproteins and Sec insertion machinery, whereas these genes were preserved in green algae, for example, *Chlamydomonas*, *Volvox *and *Ostreococcus*. An early Streptophyte, *M. viride*, has both Sec machinery and selenoproteins. Thus, there was a specific selenoprotein loss event in the Streptophyte subset of Viridiplantae, which invaded land. Analysis of selenoproteins present in green algae suggests that they were either replaced with Cys-containing homologs or entirely lost in land plants (Figure 6b). A more distantly related *C. merolae *also manifested a large-scale selenoprotein loss.

#### Apicomplexan parasites

The high selenoprotein content of *Thalassiosira *(as a reference point), the reduced selenoproteome of *Plasmodium *and the lack of selenoproteins in *Cryptosporidium parvum *illustrates an example of massive selenoprotein loss in apicomplexan parasites.

#### Fungi

We screened all completely sequenced fungal genomes and could detect neither selenoproteins nor Sec insertion machinery. These data suggest that selenoprotein genes were likely lost at the base of the fungi kingdom.

#### Insects

The small selenoproteomes of *A. gambiae*, *A. mellifera*, *D. pseudoobscura *and *D. melanogaster*, which consist of one to three selenoproteins, is an additional example of large-scale selenoprotein loss. On the other hand, aquatic arthropods, such as shrimp, have many selenoprotein genes (based on the expressed sequence tag (EST) analyses as the genomes are not yet available; unpublished data). Thus, it appears that selenoprotein genes were massively lost in either insects, or all terrestrial arthropods.

#### Nematodes

The selenoproteomes of *C. elegans *and *C. briggsae *have only one selenoprotein, thioredoxin reductase, and, therefore, the Sec insertion system is used to decode only a single UGA codon in these nematodes [10].

The decreased size of selenoproteomes in these five groups of organisms appears to be not only due to the loss of entire selenoprotein genes, but also due to replacement of Sec with Cys. Thus, Cys-containing homologs, while often catalytically inefficient, may occasionally compensate for selenoprotein loss [36].

### A hypothesis for association of large selenoproteomes and aquatic life

The mosaic occurrence of eukaryotic selenoproteins and their consistent loss in different phyla suggest that the decreased selenoproteome size is the result of a selective force. What could be the factors responsible for or associated with selenoprotein loss? Comparative analysis of organisms with large and small selenoproteomes shows that many of the selenoprotein-rich organisms live in aquatic environments. In contrast, almost all organisms that lack or have a small number of selenoproteins are terrestrial (Figure 6). Considering independent, large-scale selenoprotein loss in these organisms, a common denominator appears to be the non-aquatic habitat. It should be noted, however, that the differences between aquatic and terrestrial selenoproteomes are ultimately influenced by specific environmental factors that differ with habitat. Therefore, the aquatic/terrestrial association should not be viewed as the basis for selenoprotein loss/gain, but rather a convenient illustration of differences between these organisms. Once environmental factors are identified, this association may be modified to reflect these factors rather than habitat.

**Figure 7:**
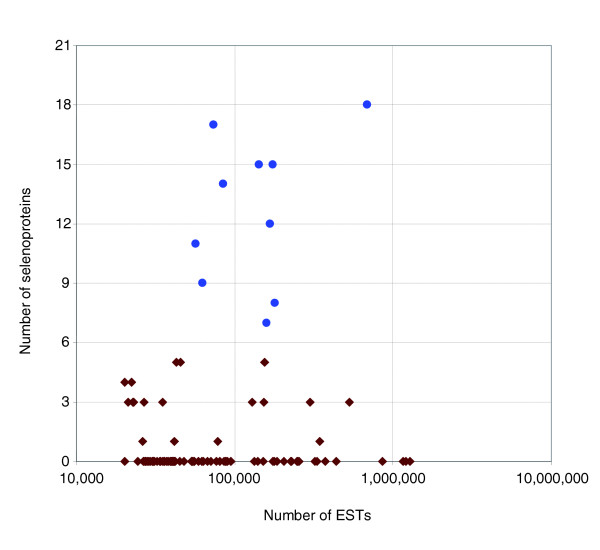
Aquatic invertebrates have more selenoproteins than terrestrial organisms. Numbers of detected selenoproteins were plotted against the total number of available (redundant) ESTs for organisms that are represented by more than 25,000 ESTs. Vertebrate ESTs were excluded from this analysis due to large size of these organisms. Blue circles correspond to aquatic and brown squares to terrestrial organisms. The difference is statistically significant (*P *value is less than 2 × 10^-6^).

To further examine selenoprotein content of aquatic and terrestrial organisms, we analyzed organisms that are well represented by ESTs. We excluded large animals (vertebrates) from this analysis because their intra-organismal environment would be less affected by environmental conditions due to availability of their outside protective cover and complex morphology. With this limitation, aquatic eukaryotes had more selenoprotein genes than terrestrial organisms (Figure 7).

Whether *C. merolae *fits this association is not clear. This organism lives in highly acidic sulfate-rich hot springs (pH 1.5, 45°C). It is possible that this extreme environment is responsible for the reduced use of Sec in red algae. The pKa of Sec is approximately 5.5. Whereas this residue would be ionized in most organisms under physiological conditions, at low pH, protonation of Sec may minimize its catalytic advantages. Abundance of sulfate in hot springs might also be of importance, as selenium and sulfur have similar chemistries.

One possible explanation for the occurrence of large selenoproteomes in aquatic organisms is bioavailability of selenium in oceans. Dissolved organic selenides can account for approximately 80% of the dissolved selenium in ocean water [37] and represent an important source of selenium for phytoplankton. Following the food chain, this could explain a large number of selenoproteins in algae and fish. Likewise, a considerable number of selenoproteins in mammals could reflect the consequence of food sources, body size and relatively recent (in evolutionary terms) emergence of these organisms from marine environments. An additional factor may be constancy in the environmental conditions and nutrients in the aquatic environments. For aquatic organisms, environmental changes are slower and involve gradients of temperature, pH, pressure, oxygen and chemical environment. In contrast, in terrestrial environments, the changes are more frequent and they happen more suddenly. As a result, terrestrial organisms often face feast and starvation situations. An attractive factor to explain the differences between aquatic and terrestrial selenoproteomes may be oxygen content. Higher content of oxygen in air than in aquatic environments may make highly reactive selenoproteins more susceptible to oxidation in terrestrial organisms and select against the use of these proteins.

Whether mammals and other vertebrates fit the hypothesis on the preferential use of selenium in aquatic environments is not clear. We note, however, that fish have larger selenoproteomes than those living in terrestrial environments, including mammals, reptiles and birds. Further genomic analyses of these organisms could clarify evolutionary changes in utilization of selenium. In future studies, it would also be important to determine which of the factors discussed above influence the preferential use of Sec in aquatic organisms or are responsible for the loss of selenoproteins in terrestrial organisms.

## Conclusion

Until recently, the mammalian selenoproteome was thought to represent accurately eukaryotic selenoproteins and to be of recent (perhaps vertebrate) origin. However, as additional genome sequences became available, selenoproteins with restricted occurrence have been identified. In mammals, these proteins either occur in the form of Cys-containing homologs or are absent altogether; instead, these rare selenoproteins have been found in several lower eukaryotic organisms. In our work, the searches of additional eukaryotic genomes identified new selenoprotein genes, revealed examples of convergent evolution of SECIS elements, and identified many features of selenoproteome organization and evolution. Integrated analyses of eukaryotic selenoproteomes suggested that the majority of eukaryotic selenoprotein families evolved in single-celled eukaryotes. Our data show that the mosaic occurrence of selenoproteins is the consequence of selective, independent selenoprotein loss events in various eukaryotic phyla. Moreover, these analyses revealed an interesting pattern: large selenoproteomes tend to occur in aquatic life, whereas the organisms that lack selenoproteins or have small selenoproteomes are mostly terrestrial (with the notable exception of mammals, whose large bodies and intra-organismal homeostasis support an internal environment that may be less dependent on habitat). Further studies will be needed to test this hypothesis and identify environmental factors that influence selenium utilization.

## Materials and methods

### Databases and programs

All genome, EST and predicted protein sequences were downloaded from NCBI [38], except for the genomes of *T. pseudonana*, *O. tauri*, and *O. lucimarinus*, which were obtained from Joint Genome Institute [39]. SECISearch [9] was used for identification of SECIS elements. FASTA package [40] and BLAST were used for similarity searches. MFOLD version 3.2 [41] was used for prediction of RNA secondary structures.

### Identification of homologs of known selenoprotein genes

Query sequences included a full set of human selenoproteins [9] as well as the following selenoproteins absent in mammals: *Chlamydomonas *MsrA [12], *Gallus gallus *SelU [42], *Danio rerio *SelJ and Fep15 [15,16], and *Emiliania huxleyi *protein disulfide isomerase [43]. A stand-alone version of TBLASTN program was utilized for detection of nucleotide sequences corresponding to known selenoprotein families. A candidate Sec residue should correspond to a Sec residue in a known selenoprotein family or a Cys residue in orthologous proteins in order to be considered further. Downstream regions of predicted selenoprotein sequences were analyzed for the presence of candidate SECIS elements using SECISearch and for SECIS-like structures using MFOLD [41]. All detected SECIS candidates were further examined for compliance with the current SECIS consensus model.

### Searches for SECIS elements

Nucleotide sequences were scanned using SECISearch (Additional data file 1). In addition, the default and loose patterns of SECISearch were modified as described elsewhere [12] to accommodate organism-specific selenoprotein searches. These modifications allowed increased sensitivity of SECISearch and supported identification of unusual SECIS structures. The overall strategy of the searches was similar to that previously described [9]. Statistics of the searches (numbers of candidates corresponding to different steps in the search process) are shown in Table 1. In an additional search for *D. discoideum *SECIS elements, the following pattern was used as a query: TGTAATGATT_(10-12 nucleotides)_AAA_(24-35 nucleotides)_TGAT. This search then continued as described for other organisms.

The primary sequence analysis step included searches for SECIS-like structures that satisfy NTGA__AA__GA or NTGA__CC__GA (N is any nucleotide) motifs in nucleotide sequences. Additional requirements were that the distance between the quartet (NTGA) and the unpaired AA in the apical loop is 10-13 nucleotides, and the distance between the unpaired AA and the GA that base-paired with the quartet is 15-39 nucleotides.

The secondary structure analysis step examined for consistency with the eukaryotic SECIS element consensus. Several additional filters were implemented to filter out candidates with unsuitable secondary structures, including SECIS elements with more than two unpaired nucleotides in a row and Y-shaped SECIS elements.

The free energy for each candidate structure was estimated; the free energies for the whole structure (threshold value of -12.6 kcal/mol) and the upper stem-loop (threshold value of -3.7 kcal/mol) were calculated. Only thermodynamically stable structures were considered further.

Based on the location of candidate SECIS elements, candidate ORFs were predicted in upstream regions. SECIS candidates located within coding regions of known proteins were filtered out. An additional requirement was the presence of at least one homologous protein in the NCBI non-redundant database. If SECIS elements and ORFs corresponding to known protein families were on different DNA strands, the candidates were filtered out.

The final step included manual sequence analyses of predicted selenoprotein ORFs located upstream of candidate SECIS elements.

### Searches using the Sec/Cys homology approach

For three organisms, *O. tauri*, *O. lucimarinus *and *C. merolae*, additional procedures for selenoprotein detection included the search for Sec/Cys pairs in homologous sequences. ORFs with in-frame TGA codons were extracted that satisfied the following criteria: Sec-flanking regions for these proteins were conserved; and homologs could be detected that contained Cys in place of Sec. TBLASTX was used to examine all potential ORFs with in-frame UGA codons against NCBI non-redundant protein database. All hits were then tested for the occurrence of SECIS elements. Orthologous proteins were defined as bidirectional best hits. PSI-BLAST was used for identification of distant homologs. Homologs were further confirmed by phylogenetic trees construction.

### Phylogenetic analyses

Numerous attempts to derive a tree of life using various methods that were based on genes encoding ribosomal RNAs and several proteins have been published. However, their principle existence has been questioned recently because of either an insufficient amount of discriminating characters or other biases such as horizontal gene transfer and chimerism. To avoid such problems, we adopted a eukaryotic branch of a phylogenetic tree recently developed by Ciccarelli *et al*. [33]. This highly resolved tree of life utilized 31 concatenated, universally occurring genes with indisputable orthology in 191 species with completed genomes across all three domains of life. The missing organisms were filled in using a 'Tree of Life' web project [44] and selected publications 5-48]. Although the horizontal gene transfer is highly prevalent in prokaryotes, it is less so in eukaryotes, particularly in multicellular organisms. We also analyzed selenoprotein evolution in the eukaryotic domain. To reconstruct the phylogenies of selenoproteins, we adopted a character-based tree estimation method, a maximum parsimony approach that implies that the preferred phylogenetic tree is the tree that requires the least number of evolutionary changes.

### Metabolic labeling of *D. discoideum *and *O. tauri *cells

*D. discoideum *cells were grown as previously described [31], the medium was supplemented with 100 μCi of ^75^Se [selenite] (University of Missouri Research Reactor), and the cells were further maintained under continuous shaking for two days. A similar procedure was used for labeling *O. tauri *cells, except that they were grown in K-medium. The radioactive bands were visualized on the gel with a PhosphorImager. Samples of ^75^Se-labeled mammalian HEK 293 and CV-1 cells were included, which were prepared as described previously [49].

## Abbreviations

Cys, cysteine; EST, expressed sequence tag; GPx, glutathione peroxidase; MSP, membrane selenoprotein; ORF, open reading frame; Sec, selenocysteine; SECIS, selenocysteine insertion sequence; TR, thioredoxin reductase.

## Authors' contributions

AVL, DEF and YZ performed computational analyses. DEF and AS carried out experimental analyses. AVL, DLH and VNG wrote the manuscript. All authors read and approved the final manuscript.

## Note added in proof

Two recent studies reported the complete genomes of *O. tauri *and *O. lucimarinus *[50,51]. One of these articles identified 18 and 20 selenoprotein genes in *O. tauri *and *O. lucimarinus*, respectively [51]. Compared to our analyses, this published study did not detect 17 selenoproteins in the two organisms, whereas the protein they designated as SelA and predicted to contain three selenocysteines appears to be a false positive. Nevertheless, the large number of detected selenoproteins in *Ostreococcus *further highlights the association with aquatic life reported in our work.

## Additional data files

The following additional data are available with the online version of this paper. Additional data file 1 presents a block-scheme of the searches for selenoprotein genes. Additional data file 2 contains amino acid sequence alignments of selenoproteins identified in this study. Additional data file 3 contains sequence and predicted clover-leaf structure of T. pseudonana Sec tRNA. Additional data file 4 has representative phylogenetic trees of selenoproteins.

## Supplementary Material

Additional data file 1 Block-scheme of the searches for selenoprotein genes. Click here for file

Additional data file 2 Amino acid sequence alignments of selenoproteins identified in this study. Click here for file

Additional data file 3 Sequence and predicted clover-leaf structure of *T. pseudonana *Sec tRNA. Click here for file

Additional data file 4 Representative phylogenetic trees of selenoproteins. Click here for file
