# Supplementary figures and images for: Evolutionary dynamics of eukaryotic selenoproteomes: large selenoproteomes may associate with aquatic life and small with terrestrial life
Source: Genome Biol. 2007 Sep 19;8(9):R198. doi: 10.1186/gb-2007-8-9-r198 (PMC2375036; doi:10.1186/gb-2007-8-9-r198)

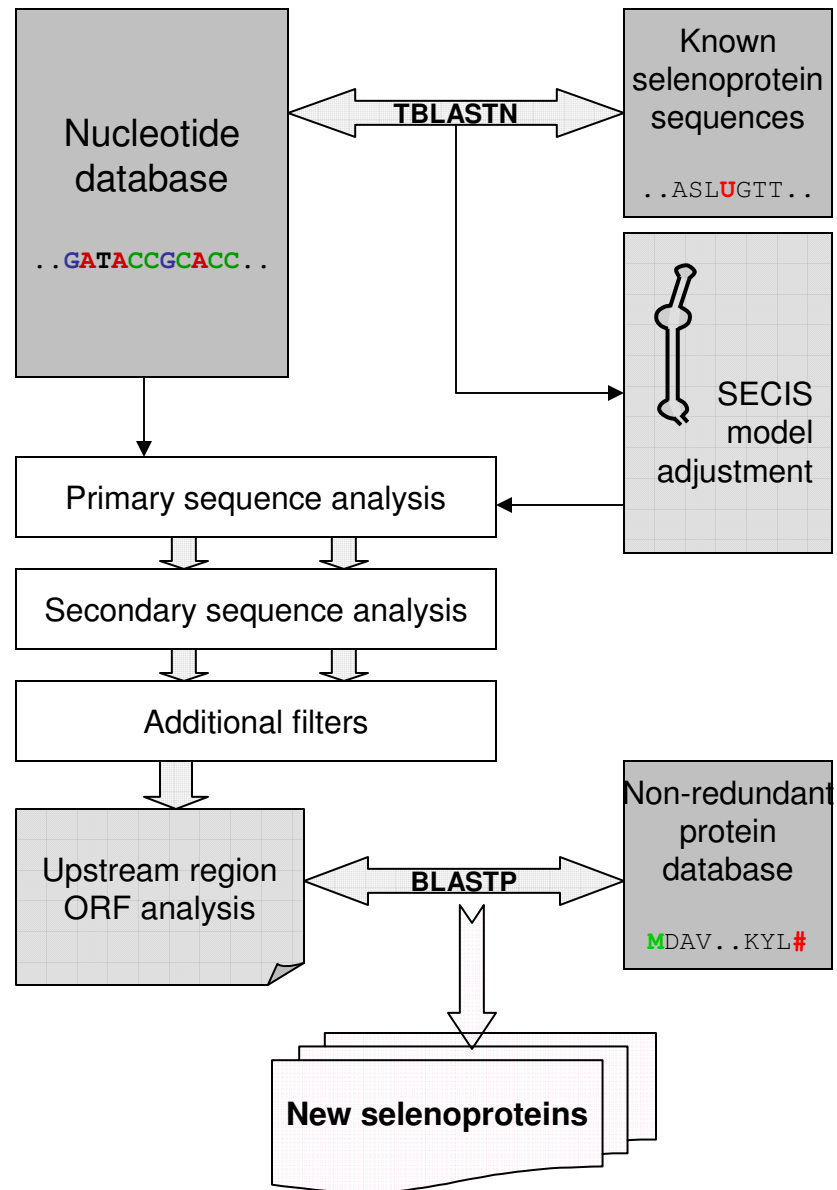

Supplement: Additional data file 1 — Block-scheme of the searches for selenoprotein genes. [file gb-2007-8-9-r198-S1.pdf]
