# Supplementary material for: Evolutionary dynamics of eukaryotic selenoproteomes: large selenoproteomes may associate with aquatic life and small with terrestrial life
Source: Genome Biol. 2007 Sep 19;8(9):R198. doi: 10.1186/gb-2007-8-9-r198 (PMC2375036; doi:10.1186/gb-2007-8-9-r198)
Supplement: Additional data file 2 — Amino acid sequence alignments of selenoproteins identified in this study. [file gb-2007-8-9-r198-S2.pdf]

The following sequences were used in protein sequence alignments:

Protein disulfide isomerase 1:
O. tauri (CR954199.1), *E. huxleyi* (BAD98262.1), *T. brucei* (AAZ12643.1), O. sativa (NP_910169.1), *A. thaliana* (NP_001031555.1), *D. discoideum* (AAB86685.1), *T. pseudonana* (AAFD01001012.1), *O. lucimarinus* (Chr_1 [128917, 129582]).

Protein disulfide isomerases 2 and 3:
O. tauri (CR954212.2, CR954213.2), *D. aromatica* (YP_284260.1), *C. intestinalis* (AK175004.1), O. sativa (NP_919053.1), *A. thaliana* (NP_198706.1), *T.pseudonana* (AAFD01000009.1), *O. lucimarinus* (Chr_12 [327601, 328434], Chr_13 [41676, 42341]).

Alkyl hydroperoxide reductase:
O. tauri (CR954209.2), *O.batsensis* (ZP_00998681.1), *B. mallei* (AAU50065.1), *B. suis* (AAN29421.1), *T. pseudonana* (AAFD01000669.1, AAFD01001369.1), *O. lucimarinus* (Chr_21 [297076, 297594]).

Methionine sulfoxide reductase A:
O. tauri (CR954209.2), *A. bacterium* (YP_591088.1), *C. reinhardtii* (AAN32904.1), *L. sativa* (AAF19789.1), *T. pseudonana* (AAFD01000063.1), *O. lucimarinus* (Chr_9 [236068, 236619]).

SAM dependent methyltransferase:
O. tauri (CR954221.1), MLMS-1 delta proteobacterium (EAT05729.1), *M. acetivorans* (AAM07134.1), *T. pseudonana* (AAFD01001090.1), *O. lucimarinus* (Chr_16 [125451, 126959]).

Selenoprotein H:
H. sapiens (Q8IZQ5), *C. reinhardtii* (ALO_fgenesh2_pg.C_scaffold_79000012), *D. melanogaster* (28571177), *O. tauri* (CR954200.1), *O. sativa* (AAX95353.1), *A. thaliana* (AAT69174.1), *M. musculus* (AAH56177.2), *O. lucimarinus* (Chr_2 [844937, 845995]).

Selenoprotein M:
O. tauri (CR954200.1), *M. musculus* (NP_444497), *H. sapiens* (AAH13421.1), *C. reinhardtii* (AAN32900.1), *T. pseudonana* (AAFD01000636.1, AAFD01000635.1), *O. lucimarinus* (Chr_4 [249088, 248666]).

Selenoprotein U:
O. tauri (CR954209.2), *H. sapiens* (AAQ89418.1), *M. musculus* (AAH56635.1), *C. elegans* (CAA91349.1), *C. reinhardtii* (ALO_ALO_fgenesh1_est.C_scaffold_38000031), *G. gallus* (CV860672.1), *T. pseudonana* (AAFD01000083.1, AAFD01001297.1), *O. lucimarinus* (Chr_9 [184824, 185411]).

Selenoprotein Sep15:
O. tauri (CR954210.2), *H. sapiens* (NP_004252.2), *M. musculus* (Q9ERR7), *D. rerio* (AAO65272.1), *O. lucimarinus* (Chr_10 [235706, 236176]).

Trx-fold selenoprotein:
O. tauri (CR954201.2), *O. sativa* (XP_469608.1), *A. thaliana* (NP_201385.2), *G. gallus* (XP_425044.1), *T. pseudonana* (AAFD01000908.1), *O. lucimarinus* (Chr_1 [945135, 945881]).

Selenoprotein W:
O. tauri (CR954199.1), *H. sapiens* (NP_003000.1), *M. musculus* (NP_033182.1), *D. rerio* (AAO86696.1), *C. reinhardtii* (BU654801.1, BP092691.1), *O. lucimarinus* (Chr_1 [1078795, 1078325], Chr_9 [279715, 279981]).

Selenoprotein T:
O. tauri (CR954203.2), *H. sapiens* (AAH26350.2), *G. gallus* (NP_001006557.2), *C. elegans* (CAB01684.1), *O. sativa* (NP_915340.1), *A. thaliana* (BAD43801.1), *C. reinhardtii* (BQ818029.1), *T. pseudonana* (AAFD01000527.1), *O. lucimarinus* (Chr_3 [801397, 802044]).

Thioredoxin reductase:
O. tauri (CR954199.1), *C. reinhardtii* (AAN32903.1), *M. musculus* (NP_056577.2), *H. sapiens* (NP_877419.1), *G. gallus* (XP_414371.1), *T. pseudonana* (newV2.0.genewise.318.11.1), *O. lucimarinus* (Chr_1 [733779, 735293]).

Selenoprotein S
O. tauri (CR954210.2), *H. sapiens* (AAI07775.1), *M. musculus* (Q9BCZ4), *G. gallus* (NP_001019905), *O. lucimarinus* (Chr_10 [15008, 15424]).

Selenoprotein O
O. tauri (CR954218.2), *H. sapiens* (AAP85540.1), *M. musculus* (NP_082181.2), *A. thaliana* (NP_196807.2), *O. sativa* (BAD61584.1), *O. lucimarinus* (Chr_17 [328041, 330110]).

Selenoprotein K
O. tauri (CR954214.2), *H. sapiens* (AAH13162.2), *M. musculus* (Q9JLJ1), *G. gallus* (NP_001020612.1), *C. reinhardtii* (AAN32902.1), *D. discoideum* (XP_646897.1), *D. melanogaster* (NP_572763.3), *D. pseudoobscura* (Contig7446_Contig2444), *O. lucimarinus* (Chr_20 [286697, 286966]).

Selenophosphate synthetase:
D. discoideum (U68248.1), *D. pseudoobscura* (Contig7891_Contig7492A.fa), *D. melanogaster* (NP_477478.4), *H. sapiens* (NP_036380.2), *D. rerio* (AAO65274.1), *C. elegans* (NP_502604.1)

Iodothyronine deiodinase:
D. rerio (AAO65268.1), *G. gallus* (CAA72136.1), *H. sapiens* (AAH17717.2), *D. discoideum* (AC115598.2)

Glutathione peroxidase:
O. tauri (CR954207.2, CR954205.2, CR954200.1, CR954208.2, CR954209.2), *D. rerio* (AAO86703.1), *O. sativa* (XP_473459.1), *C. reinhardtii* (AAL14348.1), *A. thaliana* (CAB78203.1), *M. musculus* (AAH86649.1), *H. sapiens* (P07203), *C. elegans* (CAB03004.1), *Chlamydomonas sp.*W80 (BAA83594.1), *T. pseudonana* (newV2.0.genewise.27.59.1, newV2.0.genewise.61.23.1), *O. lucimarinus* (Chr_8 [565963, 566670], Chr_5 [241440, 242054], Chr_9 [80255, 80815], Chr_7 [49879, 50784], Chr_2 [754675, 755391]).

MSP:
O. tauri (CR954210.2), *D. discoideum* (JC2V2_0_02852), *C. reinhardtii* (1031014H11.y1), *V. carteri* (ABSY206275.g1), *O. lucimarinus* (Chr_1 [146656, 147396]).

Hypothetical protein 1:
O. tauri (CR954221.1), *O. lucimarinus* (Chr_1 [180994, 181551]).

Hypothetical protein 2:
O. tauri (CR954216.2), *O. lucimarinus* (Chr_15 [364871, 365098]).

Hypothetical protein 3:
O.tauri (CR954208.2), *O. lucimarinus* (Chr_8 [589802, 590269]).

Prx-like protein:
O. lucimarinus (Chr-4 [713178, 713915]).

Dictyostelium discoideum deiodinase-like protein

```
D.dictyostelium     1 ----------------MNYLFNIFEEEKPIKKAPIITEECYRNG--------YHPSKWEL
D.rerio             1 -MGSAVGFALRKLFVYISAVLMVCAAILQMSMLKLLSFISPGR---------MRKIHMKM
G.gallus            1 ----------------AACILLFPRFLLTAVMLWLLDFLCIRKKMLT-----MPTAEEAA
H.sapiens           1 MLRSLLLHSLRLCAQTASCLVLFPRFLGTAFMLWLLDFLCIRKHFLGRRRRGQPEPEVEL

D.dictyostelium    37 A----QVLLNG---IWKPYKFKSYMQYKSRERLNLQADTQCPDAILLDDQTNQEVSLYDL
D.rerio            51 G--ERSTMTQNPKFRYEDWGPAFFSLAFIKTLFFVNWCSLGLEAFEGHAAPDSALITLD-
G.gallus           40 G-AGEGPPPDDPPVCVSDS-NRMFTLESLKAVWHGQKLDFFKSAHVGSPAPNPEVIQLDG
H.sapiens          61 NSEGEEVPPDDPPICVSDD-NRLCTLASLKAVWHGQKLDFFKQAHEGGPAPNSEVVLPDG

                                              *
D.dictyostelium    90 FNSNNNEKSVQ-DRPMVLICGSFTUPPFRDKMCMFQDIFVDFKEWVDIYIVYLKEIHPAD
D.rerio           108 RQKTSVHRFLKGNRPLVLSFGSCTUPPFLYKLDEFKQLVKDFSNVADFLIVYLAEAHATD
G.gallus           98 QKRLRILDFARGKRPLILNFGSCTUPPFMARLRSFRRLAADFVDIADFLLVYIEEAHPSD
H.sapiens         120 FQSQHILDYAQGNRPLVLNFGSCTUPPFMARMSAFQRLVTKYQRDVDFLIIYIEEAHPSD

D.dictyostelium   148 EWYIGGDEISLCYRQPKTMEDRREIIKDLKEYAPFCTIPFLIDKMDNNFNKVYDAVPERL
D.rerio           168 AWAFKNN-VDIS--VHKNLEERLAAARTLLKEDPPC--PVVVDEMNNITASKYGALPERL
G.gallus          158 GWVSSDAAYSIP--KHQCLQDRLRAAQLMREGAPDC--PLAVDTMDNASSAAYGAYFERL
H.sapiens         180 GWVTTDSPYIIP--QHRSLEDRVSAARVLQQGAPGC--ALVLDTMANSSSSAYGAYFERL

D.dictyostelium   208 YVLEDKKFKYVGGPGPFGFIPEELREFLTKRYKPHLLDLKNNLSIPPSQ
D.rerio           223 YVIQSGKVIYQASDLGGQA------------------------------
G.gallus          214 YVIQEEKVMYQGGRGPEGYKISELRTWLDQ-YKTRLQSPGAVVIQV---
H.sapiens         236 YVIQSGTIMYQGGRGPDGYQVSELRTWLER-YDEQLH--GARPRRV---
```

Protein disulfide isomerase 1

```
A.thaliana        1 MAKSQIWFGFALLALLLVS-------AVADDVVVLTDDSFEKEVGKDKGALVEFYAPWCG
O.sativa          1 MATPQISRKALASLLLLVAAAAAVSTASADDVLALTESTFEKEVGQDRAALVEFYAPWCG
E.huxleyi         1 ------------------------------------------------------------
O.lucimarinus     1 ------------------------------------------------------------
O.tauri           1 ------------------------------------------------------------
T.pseudonana      1 ------------------------------------------------------------
D.discoideum      1 MKILLFVTLIALAFVALCS--------AEGNVVVLSPDNFDTVVDGSKTVFVKFYAPWCG
T.brucei          1 ------------------------------------------------------------

A.thaliana       54 HCKKLAPEYEKLGASFKKAKS-VLIAKVDCD--EQKSVCTKYGVSGYPTIQWFPKGSLEP
O.sativa         61 HCKKLAPEYEKLGASFKKAKS-VLIAKVDCD--EHKSVCSKYGVSGYPTIQWFPKGSLEP
E.huxleyi         1 -----------------MALRSLTLLCAAAG----------------------------A
O.lucimarinus     1 -----------MATMARVVAMAFATLALVAG----------------------------A
O.tauri           1 -----------MARSFALVVAAIAALFASS-----------------------------A
T.pseudonana      1 -----------------MKTTAFALLAMAAT----------------------------A
D.discoideum     53 HCKKLAPDFEILADTFAPVSNKVVIAKVDCDQADNKALCSKYDVSGYPTLKIFDK-STTA
T.brucei          1 -----------MRNSFALLLLSVAIAFVTVG-----------S---------FAD---EA

A.thaliana      111 QKYEGPRNAEALAEYVNKEGGTNVKLAAVPQNVVVLTPDNFDEIVLDQNKDVLVEFYAPW
O.sativa        118 KKYEGQRTAEALAEYVNSEAATNVKIAAVPSSVVVLTPETFDSVVLDETKDVLVEFYAPW
E.huxleyi        16 SAG-----------------------------AIELTPDNFDELVLKSGKAAFIKFLAPW
O.lucimarinus    22 DAS-----------------------------AFVGTDANFDREVMNSGKHVFVKYLAPW
O.tauri          21 EAS-----------------------------AFLATDKNFDSAVLNSGKHAFVKFLAPW
T.pseudonana     16 TA------------------------------SLELTPDNWDAET--AGKTVFIKFLAPW
D.discoideum    112 KDYNGARSVDELLTYINNHAKTNVKVKKAPSNVVDLSPSNFDSVVLDKSKNVLVEFYAPW
T.brucei         27 KDS------------------------------VELTPDNFDKVALDTEKHVFVMFYAPW

                    *
A.thaliana      171 CGHCKSLAPTYEKVATVFKQEEGVVIANLDADA--HKALGEKYGVSGFPTLKFFPKDNKA
O.sativa        178 CGHCKHLAPIYEKLASVYKQDEGVVIANLDADK--HTALAEKYGVSGFPTLKFFPKGNKA
E.huxleyi        47 UGHCKKMKPDWDSLASTFEDSKKVLIADVDCTTG-GKPLCEKYGVRGYPTIKYFNPPDEE
O.lucimarinus    53 UGHCKSMKPAWDRLGADYKDSASVMIVDVDCTAE-GSGTCAKVGVKGYPTIKYYPAGDKK
O.tauri          52 UGHCKSMKPAWDRLGADYKDSASVIIADVDCTAE-GSGTCNKVGVQGYPTIKYYTAGDKK
T.pseudonana     44 UGHCKKMKPDWDKLAEKFAGSATQLVADVDCTTE-GKPLCDANGVRGYPTIKWGDPADLQ
D.discoideum    172 CGHCKKLMPDYEILGNTYANEKDVVIAKIDCDAADNKAICSKYGVTGFPTLKWFGKQSKD
T.brucei         57 CGHCKRLKPKWEELAKEMKDETSVVIARLDADK--HRNVAERFDVRGYPTLLLFARSKKE

A.thaliana      229 GHDYDGGRDLDDFVSFINEKSGTSRDSKGQLTSKAGIVESLDALVKELVAASEDEKKAVL
O.sativa        236 GEDYDGGRELDDFVKFINEKCGTSRDSKGQLTSEAGIVESLAPLVKEFLGAANDKRKEAL
E.huxleyi       106 GEDYKGGRSLDELKKFAENELG----PGCSVDLMDNCSEEQKGKLKEYIDMAPEKRTEML
O.lucimarinus   112 GKDYQGGRDYDALKSFVTKTLDK---PLCDAATKKGCAKNEVEFLKKMDGKSKSELKSEL
O.tauri         111 GKDYQGGRDYDELKKFVTKTLDK---PLCDAVTKKGCAKNEIEFLNKMDGKSKAELKSEL
T.pseudonana    103 --DYQGGRDYVALEKFATENLK----PVCSPKNIDLCDDDKKADIKKFLEMAPADLDALI
D.discoideum    232 GEKYEQGRDLDTFINYINKQAGVNRVKGGKLAVGAGRVEQLDTIATEFIAAAAEVRKELV
T.brucei        115 GLRYEGARDVAALKEFVKSNM---------------------------------------

A.thaliana      289 SRIEEEASTLKGSTTRY--------VTLLETRKELHRK---RFRLC--------------
O.sativa        296 SKMEEDVAKLTGPAAKYGKIYVNSAKKIMEKGSEYTKKESERLQRMLEKSISPSKADEFV
E.huxleyi       162 ETLKKELADAESTHEALLKELQATYKES-MDKLEKLKEESAPKIKLLKAATPAPKAEGAK
O.lucimarinus   169 KEKTAELNEIKKEMKAATKEFNAKTQAW-----KKRQAVINRANKLLNDLIAASDK----
O.tauri         168 KEKETETAEIKKEMKAATKEFNTKTQAW-----KKRQAVLARAQKLLKDLIKAPDTN---
T.pseudonana    157 AAEEQKLEDAEASFKDEVQKLQDKYQALSTEKDEKIAAVKASGLGLMKSVKASAPASGS-
D.discoideum    292 KKAQTVVDSLPEELRIEGSYYVKVMKTIAEKSIDFVTTEIARITKLVSGSMSGKKADEFA
T.brucei            ------------------------------------------------------------

A.thaliana          ------------
O.sativa        356 IKKNILSTFSS-
E.huxleyi       221 DEV---------
O.lucimarinus   220 TEL---------
O.tauri         220 AEL---------
T.pseudonana    216 DEL---------
D.discoideum    352 KKLNILESFKSK
T.brucei            ------------
```

Protein disulfide isomerases 2 and 3

```
C.intestinalis          1 --------------------------------------------------MSKFLHIRVV
D.aromatica             1 -----------------------------------------------------MLTIEIV
A.thaliana              1 ------------------------------MAESAS-----------NTASKKLIQIDVS
O.sativa                1 MNQIKSNPQIKVNQNSVAEVSLPSSSWSRLLAQAASPPHNWTRIYMASNAGKKLIQIDVS
T.pseudonana            1 --------------------------------------------------------MWYP
O.tauri PDI2            1 --------------------------------MR---------GVKLARGRHQLVCVELV
O.lucimarinus PDI2      1 --------------------------------MRRAAREAARVGTRGARAKHQLVTVELV
O.tauri PDI3            1 ------------------------------------------------MTTRLPLVIDVV
O.lucimarinus PDI3      1 ------------------------------------------------MTTRQ-LTIQIV

                              *
C.intestinalis         11 SDIMUPWCWVGKRNLETAMKASENE----------YKFKVTWE----PFLLRPNMPL-E-
D.aromatica             8 SDIVCPWCFIGLRRLDLAIAQVRQEI-------PDFSCTKRWR----PFFLNPDTPP-E-
A.thaliana             20 SDSVCPWCFVGKKNLDKAIEASKDQY----------NFEIRWR----PFFLDPSAPK-E-
O.sativa               61 SDTVCPWCFVGKKNLEKAMEQNKDKF----------DFEVRWH----PFFLNPNAPK-E-
T.pseudonana            5 VNSIUPWCFIGKRRMEEALDDAKTRYPH-------LTFDVRWR----PFELDPRLPTGE-
O.tauri PDI3           13 SDIVUPWCFVGKRHLERAVRAL-----------------TSKD----SN------QK-V-
O.lucimarinus PDI3     12 SDVVUPWCYVGVKNLDRARAALRPDVASSRAVWRPFQLVSSHD----WRRMGADVAR-A-
O.tauri PDI2           20 SDTMUPNGFIGKRHLERAMRAVTEKP-------LDVSNPVRFSVIRVPFFLEPEYPRNES
O.lucimarinus PDI2     29 SDTMUPNGYIGKRQLQRAMRAVQREP-------LDANNPLRFTVLRVPFFLEPEYPRDEA

C.intestinalis         55 -GIAKQGDFG-------PHTPGAQRLINVGRKVGVEFAFKQPRYPYT-LFGHCALEFAIK
D.aromatica            55 -GEPYLPFLEKKFGGRAPVEALFERVRAAGRPYGLDYAFEKIERRAN-TLQAHRLIHWAQ
A.thaliana             64 -GVSKKEFYLQKYG--NRYQGMFARMSEVFKGLGLEFDTA--GLTGN-SLDSHRLIHYTG
O.sativa              105 -GIKKSDYYRMKFGP-IQFEHATARMTEIFRGLGMEYDMS--GLTGN-TMDSHRLITLAG
T.pseudonana           53 -GKDKMKHYEAKFGA-DNIRRMIPRMKATAREHGINMEYG--GNVGN-TFDSHRLIWKAR
O.tauri PDI3           44 -EVN---WR-----------PFEL------------------------------------
O.lucimarinus PDI3     66 -GVNKRSWYNERFGA-DTVATFEPRLASAFAKAGIEGAYTLDGNTGD-TRPAHRVAAYAE
O.tauri PDI2           73 WSETNRTRLERKWGGKAQFEAQKRRHRLKERGREVGIDSFNLDRLASSTMQSHRLVQWVT
O.lucimarinus PDI2     82 WSETNRTRLERKWGGKREFDAQKRRHRLKERGEDVGIKHFNLDRLASSTMASHRLVQWVT

C.intestinalis        106 KDPSGSIQTQLQESLFKSYFTDGEYP-DVETVSTVAATCGLNREEVKSFISDESN--LAA
D.aromatica           113 QHG---DAERLVERLFVAQFQRGEAVSDPALLVRLAAECGYPAATVADYLASSED--ADT
A.thaliana            118 KQA-PEKQHTLVEELFIGYFTQGKFIGDREFLVETANKVGIEG--AEEFLSDPNNG-VTE
O.sativa              160 HQG-YDKQSALVEELFQSYFCHGKFIGDRQVLLDAARKVGIEG--AEELLQDSNKG-VDE
T.pseudonana          108 EVGGSELQDKVVESIFKAYFEENKSLGDSAVLEECANRAGMKD--TKDFLTNSQLG-RDE
O.tauri PDI3           53 -------QDKFMEAIFTRYFLKGETPCDRDVLLAAATEAGLDREECEKVIADDDA--FAA
O.lucimarinus PDI3    123 ETHGPAAQDAFMRAMFHRYFIEALAPCDEAVMRDAASAAGLDEAAVSKVLADGEASPFET
O.tauri PDI2          133 KHHGCAVSEKLYNELNRRHFVEGQKLNDREMLCDAAAAAGLDRAKAEEFLASGEG--EAE
O.lucimarinus PDI2    142 KNHGCTASETLYNDLNKRHFEDGQKLNDKRMLAEAAARVGVDANEAMEFLQSGEG--EME

D.aromatica           168 VRAMEAEVRAAG-ISMVPTFIVDR-------KMVVVGAEDPSILAGAIRQVLAERG----
C.intestinalis        163 VKRKAAQWSANG-VSGVPYFIINDCP-------VFSGAQEPAAFNI--------------
A.thaliana            174 VKEELAKYSKN--ITGVPNYTING-------KVKLSGAQPPETFQSAFKAASA-------
O.sativa              216 VKEELNKYSSG--ISGVPHFVING-------KFQLSGGQPPNAFTRAFDVAAKDGAQ---
T.pseudonana          165 VEREKNEFGRAFQCNGVPMFVIDE-------RFVLHGAQEKEAFLRTFGKVSMEL-----
O.tauri PDI3          104 EVEEQKRRFAAR-VSGVPHFIISHGG----RRLEFGGAQPPDVFAEAFVDLLGIDELVVE
O.lucimarinus PDI3    183 VVEEQMSATRAR-VRGVPHFIITCDGDGASRKIEIGGAQPPEAFLDAFAELLDLDADDVA
O.tauri PDI2          191 IEGALHVLRRMG-IHSIPNFIIGA-------KHVLSGAVHSSELINVFRDIERTGKGAPE
O.lucimarinus PDI2    200 IEGALLILRKMG-INSIPNFIVGA-------QHILSGAVHSSELIKLFRQIERTGKGAPD

D.aromatica               ----------------------------
C.intestinalis            ----------------------------
A.thaliana                ----------------------------
O.sativa                  ----------------------------
T.pseudonana              ----------------------------
O.tauri PDI3          159 ----------------------------
O.lucimarinus PDI3    242 ATKS------------------------
O.tauri PDI2          243 SAFSAILGIPDEVVEKPLDKSYFAEASA
O.lucimarinus PDI2    252 SAFAAVLGIGDDVIARPLDASYN-EASA
```

Peroxiredoxin

```
B.mallei          1   MTDETADDRPRRFATPGRVSRPLRMREAGLGRVVCRERPYRYEGPGMIQVGDTLPDAQLF
B.suis            1   MT---------------------------------------------IKVGDRLPAATFK
O.batsensis       1   MA---------------------------------------------ISKGDTLPDATLV
O.lucimarinus     1   MVA----------------------------------------------VGDQFPDVVAQ
O.tauri           1   MVA----------------------------------------------VGDAFPDVVAQ
T.pseudonana      1   MCS-------------------------------------------------RVPSLHLL
T.pseudonana 2    1   MC--------------------------------------------------LLPSVELH

                                                          *
B.mallei          61  EYLDDARAGCTLGPNAFGVREQTAGKRVVIFGLPGAFTPTCSAQHVPGYVAHAEPLRSA-
B.suis            16  VKTADGVTEMTT-DDVF------KGRKVVLFAVPGAFTPTCSLNHLPGYLENRDAILAK-
O.batsensis       16  EFTAEGPKEVSL-SERL------AGRNVVIFALPGAYTGTCTTAHVPSFIRTVDQFRDK-
O.lucimarinus     15  SHEPSWPTPVKMRARLA-------GKKSLVVGLPGAFTPTUSTCQVPGYLAGQDALRAS-
O.tauri           15  SHEPTWPTPVKMRARLA-------GKRTIVVGLPGAFTPTUSTCQVPGYLAGQDALKNA-
T.pseudonana      12  CS--------NIQDYCK-------DKSVVIVGLPGAFTPTUSTKQIPDYVEKQDALRSK-
T.pseudonana 2    11  SGFP--PQKIDLATYTA-------NKSVVIIGLPGAFTPTUSNAQVPSYVELQDALHTKA

B.mallei          120 GIDEIWCVAVNDAFVMGAWGRDLHTAGK-VRMMADGSAAFTHALGLTQDLS---ARGMGI
B.suis            68  GVDQIAVVAVNDPFVMGAWAQSTGGEGK-ILFLADGSATFTKAAGLDIDLS---GGGLGV
O.batsensis       68  GVDEVICLSVNDPFVMAAWGKDTGAADAGIAMLGDPRAEFTKAVGMDFTAP---PVGLID
O.lucimarinus     67  GIDEVLVMCVNDAAVMGAWAIDQKIQGSNISFLADPNSVITKALDVELVAPGPCAKLGPK
O.tauri           67  KIDEVLVYCVNDAAVMGAWAIDQKIAGSNIAFVADPNSVMTKALDMELTHPGPCAKLGPK
T.pseudonana      56  GVDSVVILAVNDGAVMMNWAKSQKVGLSMLTFLGDPNSELTKALDLELTHPGPVGVLGPG
T.pseudonana 2    62  GIDEIIIYCVNDGAVMSAWANDLGVDKGRLTFLGDPEGVLTRKLEMEMTHPGPISVGIIG

B.mallei          176 RSRRYAMVVDDGVVKTLFVEA-------PGKFEVSDAASVLAGLTR-----
B.suis            124 RSKRYSAIVEDGVVKSLNIEEQ------PGQAVTSAASALLAQL-------
O.batsensis       125 RSKRYAMYVKDGVVTVLHAEEN------PGQCDVSGGESMLEAI-------
O.lucimarinus     127 RCKRFAMYVDDGVIKVLNVSEAPDDPAGDARPEASCIDNMLKEIAAL----
O.tauri           127 RCKRFAMFVEDGVIKVINVSEGPDDPAGDERPESSCIDNMLKDIAAL----
T.pseudonana      116 RCKRFAMHVVDGVIKAVNVSESADDPVSTKQDVLTLKPLLSRIPCQTKDRG
T.pseudonana 2    122 RCK-VAVYAVNGEVKYVAVSEAEDDPAGDENPEKTLAPAILDAIVGLKDEL
```

Methionine sulfoxide reductase A

```
A.bacterium        1 ------------------------------------------------------------
L.sativa           1 MFLLRTTTATTTPASLPLPLLSISSHLSLSKPSSFPVTSTKPLFTLRHSSSTPKIMSWLG
C.reinhardtii      1 ------------MAT--------------------------------------N------
O.lucimarinus      1 ---MRAAVATARVAAP-------------------------------------SRASANK
O.tauri            1 ---MRAS-GTVVAATS-------------------------------------SRAVNRM
T.pseudonana       1 ---MNNICPEIPTAPQ-------------------------------------K------

                                                             *
A.bacterium        1 -------------MEK------------------ATFAAGCFWGVEETFRTTPGVVATAV
L.sativa          61 RLGXGTRTPADASMDQSSIAQGPDDDIPAPGQQFAQFGAGCFWGVELAFQRVPGVSKTEV
C.reinhardtii      5 ---------------------G-NG---ASGAELATFALGUFWHPEASFANVPGVVKTRV
O.lucimarinus     21 R-NVRALAAMPASKDARRTDRG-DGFVVPENCQLAVFASGUFWGPDASFSALKGVKNVRV
O.tauri           20 RAQTTARSSASASGSLARTQRG-DGFKIPEGCELAVFASGUFWGPDATFSALKGAKHVRV
T.pseudonana      15 ----------------------------ALSNEVAVLASGUFWHPQRDFKRLEGVVDVIV

A.bacterium       30 GYTGGHTENPTYHDVCTDTTGHAEAVEVTYDPAKVSYDDLLKIFWENHNPTQMNRQGPDV
L.sativa         121 GYTQGFLHNPTYNDICSGTTNHSEVVRVQYDPKACSFDSLLDCFWERHDPTTLNRQGNDV
C.reinhardtii     40 GYTGGSRPNPTYESVCAG-DGHTEAMRVWFDPAIISYEDLLKQFFREHDPTQKSK-----
O.lucimarinus     79 GYTGGESDFPTYSAVCAGGTGHTEGYYVAYDAATTSFDKLLEQFWSEHQPTYESK-----
O.tauri           79 GYTGGETDLPTYTTVCAGSTGHTEAYYVAYSPAETSYDKLLDQFWAEHSPTYMSK-----
T.pseudonana      47 GYTGGQKKNPTYQNIMDA----TEAFIVEFDPSVISYEEILNEWAAQHAPFYPSK-----

A.bacterium       90 GAQYRSAIFFYSPEQEAKARASKEALEKSGRFSKPIVTQVVP-----AEPFYRAEEYHQQ
L.sativa         181 GTQYRSGIYFYTPEQEKAAIEAKERHQK--KLNRTVVTEILP-----AKKFYRAEEYHQQ
C.reinhardtii     94 -CQYKSAVWYHSEAHGTALAAVMKELEG--KYRVRLATTVDP-----AGDWWDAEEYHQK
O.lucimarinus    134 -AQYKSAIWAQTPEQYEKAVASKEAREK--QVQRKFFTDIYPPEVSLATPWWDAEEYHQK
O.tauri          134 -SQYKSAIWAQTPEQYEKAVASKDAAER--RVGRTMFTDIYP-----DTAWWDAEEYHQG
T.pseudonana      98 -CQYRSAIFYCSEEQQSAAQKKIEELGK--DGQRSVYVDLEP-----VSAFYRGEEYHQD

A.bacterium      145 YLLKRGRT--------------HCHI
L.sativa         234 YLAKGGRFGFRQSTEKGCNDPIRCYG
C.reinhardtii    146 YIEKSMMRRG----------SAGWW-
O.lucimarinus    191 YYNKPRVSR------------FGW--
O.tauri          186 YYRKPRVSR------------FGW--
T.pseudonana     150 FLDKQAGAR------------APMF-
```

SAM dependent methyltransferase

```
O.lucimarinus     1 MLVPRERQPAVARLRPPSTYRLHRPPRRLRVRLRRFSILVFFRFARRARAFFSNAVDLVV
O.tauri           1 ------------------------------------------------------------
T.pseudonana      1 ------------------------------------------------------------
D.p. MLMS-1       1 ------------------------------------------------------------
M.acetivorans     1 ------------------------------------------------------------

O.lucimarinus    61 VVVSFVDALFQKLRIAQDSESTAHARDLARRVRRLQRVSIDRGRRSRAPLQRS--RVARA
O.tauri           1 --------------------------------------------------MTT--PVA--
T.pseudonana      1 --------------------------------------------------AGT--GTT--
D.p. MLMS-1       1 -----MNHETRSAADVADDICVFFAKSPSIGVRRDERRNLKRKEQFMETVKNDEIRQAVR
M.acetivorans     1 ----------------------------------------------MDAAEK---KEVIK

O.lucimarinus   119 RAFDQLSSTSSSPFVSPRARFRARRARRRTARSCPSRHGSRVASSASSASSASSASSSPR
O.tauri           7 -AYDALAS------------------------------GS--------------------
T.pseudonana      7 -GIACCPG------------------------------GS--------------------
D.p. MLMS-1      56 RQYGQVAD--S----------G----------------GAG-------------------
M.acetivorans    12 KKYQEIAT--L----------G----------------GS--------------------

O.lucimarinus   179 DMARCATRRRMASHRDVVRDAYARTALSAGREGCCVT----PLDARVKIGYTRDELALAG
O.tauri          16 -------------------DAFAP---------CCVP----ESD-RAHIGYADADRAFVG
T.pseudonana     16 ------------------INQSGE----GG---CCVS----VKP--EALGYTQEQIIKAG
D.p. MLMS-1      69 ------------------CGCSTT---------CCDAPGASAEVLSQGLGYTADDVAGVP
M.acetivorans    24 ------------------CCSGGG---------CCGD--LSAADLSRSLGYSEADVQAVP

O.lucimarinus   235 -GANLGVGCGAPHQFAELQAGEAVCDLGCGAGVDVVLAALSVGERGVVVGVDMTPEMLRE
O.tauri          43 -DADLGLGCGAPVTRAMLAQGEAVLDLGSGGADAFLAARAVGKFGRVTGVDASESMIRR
T.pseudonana     45 KDANLGLGCGNPISFANIKEGETVVDLGSGAGVDCFLAADLVGEKGLVIGCDMTPDMIYK
D.p. MLMS-1     102 MGANMGLGCGNPQAIAGLKAGEIVLDLGSGGGFDCFLAARQVGETGRVIGVDMTPEMISQ
M.acetivorans    55 D-ANLGLGCGNPTAFAELKPGDIVLDLGSGAGFDSFLAAQRVGSLGKVIGVDMTQEMVKK

O.lucimarinus   294 ARARAAEASERANADGRECARAEFRLGELERLPCRDGEFDVVMSNCVINLCEDKRAALAE
O.tauri         102 ARERA-----RT----EEISAVEFRLGELESIPVESGTYDCVISNCVINLCGDKRRAFAE
T.pseudonana    105 ARQNALN---------RKCTNATFRLGEIEHLPIADSTVDVVISNCVINLSPDKAQVFRE
D.p. MLMS-1     162 ARANATK---------SGDRNVEFRLGEIENLPVADGAVDVIISNCVINLSPEKRRVFAE
M.acetivorans   114 AQDNARK---------YGYSNVEFRQGDIEALPLDDRSVDVIISNCVINLAPDKEKVFRE

                                                          *
O.lucimarinus   354 AFRALKPGGRLCVADVVSRG-NALPEALKTNEAL-ACUVSGAHEQDILRDMLRDVGFV-R
O.tauri         153 AFRALKPGGRLCVSDVLRRHPGDLPERLRTNEAL-ACUVTGAHEEDVLRQMLRDVGFV-R
T.pseudonana    156 IHRILKPGGRIAISDVVIRPSKIIPERLKTSEAL-ACUVSGAPEMASLEQYLVDAGFK-E
D.p. MLMS-1     213 AYRILKAGGRLAISDVVATA--ELPEAVRKDMALYTGCIAGASLVSDIEQMLTEVGFT-E
M.acetivorans   165 AFRVLKPGGRMYVSDMVLLE--DLPEDLKNDCDLLAGCVAGALLKEEYLGLLKKAGFSFK

O.lucimarinus   411 AEIRVKEESREYIKHWMPGSGAEDYVVAAEVVAHKPGTLTSVVQGACKRVGELLYAAWLA
O.tauri         211 AEIVVKEESANYIKHWMPGSGAEDHVIAADVLAYKSRTFFGACERVMKRVSDVAYAVWLA
T.pseudonana    214 VDMKLKEESRAIISQWLPGSGAEDFVISAEITARK-------------------------
D.p. MLMS-1     270 IRVSTKDESKSFIRDWAPGTDVADYVVSATIEATKPAT----------------------
M.acetivorans   223 ILAEDSDVSK---RQYE-GLPVESLKLKAWV-----------------------------

O.lucimarinus   471 QARHHAAHTDHSQDDDEPECCAPGPAKKPLPKC--
O.tauri         271 QARHHAEHTDTTPRVEEPACCTPGPEKKPVPKCUQ
T.pseudonana        -----------------------------------
D.p. MLMS-1         -----------------------------------
M.acetivorans       -----------------------------------
```

Selenoprotein H

```
H.sapiens          1 ---MAPR--------------------------GRKRKAEAAV-----VAVAEKR-----
M.musculus         1 ---MAPH--------------------------GRKRKAGAAP-----METVDKR-----
D.melanogaster     1 ---MPP----------------------------KRNKKAEAP-----IAERDAG-----
D.pseudoobscura    1 ---MPP----------------------------KRRKKVIAT-----FVPDPGA-----
A.thaliana         1 ---MASKKVDGEGKGKAIANTRMLRSMDR-KTRSDTKRDGSSSKLMKIESPEKKKRKTTK
O.sativa           1 ---MPPKRKSPAAT-AAVGSPRKTRSMAVGKQRAEAPAKAAKKEAAAAAAPPEQK-----
C.reinhardtii      1 ---MAPKRKAAAA--------------------AEAPAKKAK-------APAKKK-----
O.tauri            1 MAAKAPKKTTAKKTTAKKPAAKKPAAKRTAVKKTTATKKPAAKK-----KPAAKKP----
O.lucimarinus      1 --------------------------------RDDDFRAIAAKK-----KPAAKK-----

H.sapiens         22 --------EKLANGGEGM----------------------------------EEATVVIE
M.musculus        22 --------EKLAEG----------------------------------------ATVVIE
D.melanogaster    20 --------EELDPN---------------------------------------APVLYVE
D.pseudoobscura   20 --------AALDPS---------------------------------------QPVLYIE
A.thaliana        57 AKNVGAAKKKVKKEEVAVKIEKEEEEDDD-------------AAEKEEDDDSDKKKIVIE
O.sativa          52 ------GRKRAKKEDAQVAAAAEK--DSG-------------AVV------SDGKRIVVE
C.reinhardtii     26 --------EAEKKEETAPVAADAG----------------------------DG--IVIE
O.tauri           52 -----AAKKPAAKSTCAIFSAPRARAGTDVCVSLDFLRISAAKKKPAAKKKPAAKKATAK
O.lucimarinus     19 --------KPAAK-----------------------------K-KPAAKKKPAAKKAKAK

                         *
H.sapiens         40 HCTSURVYGRNAAALSQALR-LEAPELPVKVNPT-KPRRGSFEVTLLR---PDGSSAELW
M.musculus        34 HCTSURVYGRHAAALSQALQ-LEAPELPVQVNPS-KPRRGSFEVTLLR---SDNSRVELW
D.melanogaster    33 HCRSURVFRRRAEELHSALRERGLQQLQLQLNALGAPRRGAFELSLSAGGMGKQEQVALW
D.pseudoobscura   33 HCRSURVFRRRAEELHAALQERGLQQLQLQLNVEGTPRRGAFELHLAK--QPTTEQHFLW
A.thaliana       104 HCKQCKSFKERANEVKEGLEEA-VPGIIVTVNPD-KPRRGCFEIREEG-----GETFISL
O.sativa          85 ACTQCRQFKIRAAKVKEDLESS-VPGVSVVINPE-KPRRGCLEIREEG-----GEVFISL
C.reinhardtii     48 ACKSUGAFKTRATKLEKLIKEG-APGTSVSVNPD-KPRKGCFEVRGPG-----GKTFVSL
O.tauri          107 TSKKUGVFLRRAEHLKKLLADT--AGIDMTIERD-SSCGGFEVFVDE-------IAVVSL
O.lucimarinus     41 ASKKUGVFLRSAERLRTLLAS---AGIEVEIERD-NTCRGFEVFVDD-------VAVVSL

H.sapiens         95 TGIKKGPPRKLKFPEPQEVVEELKKYLS--------------------------------
M.musculus        89 TGIKKGPPRKLKFPEPQEVVEELKKYLS--------------------------------
D.melanogaster    93 SGLKRGPPRARKFPTVEEVYDRIVGILGDQQESKEQINTQKLSKIDLPGSEALASPKKSE
D.pseudoobscura   91 SGLKR-TPRAQKFPPVDDVFRWIVEMLGQVMEKQQ------------PGQAGDQIPKEEE
A.thaliana       157 LAMKR-PFTPMKELNMEEVIADIVEKIK--------------------------------
O.sativa         138 LNMPR-PFTEMKKLDMDEVIKDIANKIS--------------------------------
C.reinhardtii    101 LDMPR-PFTKLKALDVEALAEEVLAALKE-------------------------------
O.tauri          157 LNMPR-PFTKLRELDLEVVAKDVALLCAS-------------------------------
O.lucimarinus     90 LDMPR-PFVKLRALDLERVAKDVSSLCA--------------------------------

H.sapiens            ------------------------------------------------------------
M.musculus           ------------------------------------------------------------
D.melanogaster   153 STEEAQENEAPTSTSTSRK-SKKEQKSEEEPTQVDSKEAKQSKELVKTKRQPKAQKKQAK
D.pseudoobscura  138 PEPELKQEAVPAKKSQKRKRSAKTTKSEPEPVNVPN------------------------
A.thaliana           ------------------------------------------------------------
O.sativa             ------------------------------------------------------------
C.reinhardtii        ------------------------------------------------------------
O.tauri              ------------------------------------------------------------
O.lucimarinus        ------------------------------------------------------------

H.sapiens            --------------------------------------
M.musculus           --------------------------------------
D.melanogaster   212 ASESQEEVAEDKPPSSQKRKRTTRSSTDEATAGAKRRR
D.pseudoobscura  174 ----EEQVVS--------------------TNNAKRRK
A.thaliana           --------------------------------------
O.sativa             --------------------------------------
C.reinhardtii        --------------------------------------
O.tauri              --------------------------------------
O.lucimarinus        --------------------------------------
```

Selenoprotein M

```
                                                                                       *
H.sapiens           1 -----MS--LLLPPLALLLLAALVAPATAATAYRPDWNRLSG-LTRARVETCGGUQLNR
M.musculus          1 -----MS--ILLSPPSLLLLAALVAPATSTTNYRPDWNRLRG-LARGRVETCGGUQLNR
C.reinhardtii       1 ----------MRALALALLALALIAHEANAK-------------VARGEFISCPGURLNH
O.lucimarinus       1 MTSTATTTRTTTRRRPARRVVAARVVAACVALARVARGTAGEGAFARAQFQSCPGUKLNR
O.tauri             1 -----MT--VARRRSSASFLFAFVLFLACASSALAESATS----YARAQFQSCPGURLNR
T.pseudonana M1     1 -----MI--LVRIFPPVLLFLSMLLVWSDAQEQR-----------LTGLIESCSGUALNR
T.pseudonana M2     1 ---------MLSSASRILFLLATILALTTAT--------------LHGRIESCSGUKLNK

H.sapiens          53 LKEVKAFVTQDIPFYHN---LVMKHLPGADPELVLLGRRYEELERIPLS-EMTREE---I
M.musculus         53 LKEVKAFVTEDIQLYHN---LVMKHLPGADPELVLLSRNYQELERIPLS-QMTRDE---I
C.reinhardtii      38 LPEVKKFIRGDIEKDKAYENLTVNFVPGRTPELVLYDENGVEIERMFVD-KLKYDE---L
O.lucimarinus      61 LPEIKTFLKEVVEVGGYGERVGVTWTPGHPPSLHMQDERGNNVETAKLTSEWTVER---V
O.tauri            50 LPEIKAFIKDVVEVGKYGDKVSVLWTHGHAPTIHMQDDKGTNVESVVLTSEWTVDQ---V
T.pseudonana M1    43 LPELKSFLKDLNGVDLYKN-VEVHFISGKRAVLTIFEGKYTGVELAERR-KVEAEETGSL
T.pseudonana M2    38 LPVLKSFLKDGE-AESYRN-VEVKYVPGKQAVLTIYEG--------------EGEE---V

H.sapiens         106 NALVQELGFYRKAAPDAQVPPEYVWAPAKPPEETSDHADL-----
M.musculus        106 NALVQELGFYRKSAPEAQVPPEYLWAPAKPPEEASEHDDL-----
C.reinhardtii      94 HTLVQSKGFKRRDPNAAAATTTATASTRRARAARRLLRATEETAC
O.lucimarinus     118 ETYLNERGFYRPGQERETARVEL----------------------
O.tauri           107 KEYLSERGFDPIKQEK----SEL----------------------
T.pseudonana M1   101 QKTLEAQRYSLSKREQKLQMKERLKKLQTARDNYMIEAPFVEQ--
T.pseudonana M2    79 EGWVEKEKIVLSELETKVTVVWVLTECRFMCCN---SAVFI----
```

Selenoprotein U

```
H.sapiens          1 ----MWSIGAGALGAAALALLLANTDVFLSK-PQKAALEYLEDIDLKTLEKEPR------
M.musculus         1 --MGMWSIGVGAVGAAAVALLLANTDMFLSK-PRKAALEYLEDIDLKTLEKEPR------
G.gallus           1 ----MWSVGLGAVGAAITGIVLANTDLFLSK-PEKATLEFLEAIELKTLGSEPR------
C.elegans          1 ----MAFLGYGAAAALGGALVYANLPTYLTIGAVAPTFAHLAAAKLVPIRGGPKEKEVVE
C.reinhardtii      1 MQRSATTVGRSRPIRFAPRPLSVRVQAAAAPFATNGTTDAYNRIKGIKVYRSSD------
O.lucimarinus      1 -------------------MTAARATYSDKHTRVSASFGDVKDIRLRPLPKRDD------
O.tauri            1 -------------------------MYGDKHVQITASFDDVKDIRLRPLPLSDG------
T.pseudonana U1    1 ------------------MIWVTTSDPSLSHHLVHPQLVSLKFNDNPLVEEVDV------
T.pseudonana U2    1 ------------MSVPTRTLLTSKAIAAAQTILSAALLTGLDICDTTVKPKSPP------

                                               *
H.sapiens         50 ---TFKAKELWEKNG-AVIMAVRRPGCFLCREEAADLS-SLKSMLDQL-GVPLYAVVKEH
M.musculus        52 ---TFKAKELWEKNG-AVIMAVRRPGCFLCRAEAADLM-SLKPKLDEL-GVPLYAVVKEQ
G.gallus          50 ---TFKASELWKKNG-AVIMAVRRPGUFLCREEASELS-SLKPQLSKL-GVPLYAVVKEK
C.elegans         57 RNEQFTADSLFKKGP-IMVMAVRRPGCMLCRREAAELH-TLLPLLKEK-GIELAAVVHET
C.reinhardtii     55 -GELVDLTSLWGPNERAVVAFARSFGUFFCWELAIQLRRDVKPKLDEM-GIKLFLVSIGT
O.lucimarinus     36 -EESFAASGLWREKP-CVIVVMRRPGUVLCRGEAVKVY-ERKREFDAL-GCTLACVLKEG
O.tauri           30 -AAAFAASELWREKP-CVMVVMRRPGUILCRGEALKVR-AAKPELDAL-GVTLACVLKEG
T.pseudonana U1   37 FTGEEVLQQNVGSIG-TLVFVVRRPGUPLVRGDARILQ-SKIDASQAMEGFKFLSIVKET
T.pseudonana U2   43 KPLSEILPEQTGSAG-SICFVVRRPGUVLCREHGRQLM-DLANKSSEMKEFKLFGTVKEI

H.sapiens        104 IRTE--VKDFQPYFKG-EIFLDEKKKFYGP-QRRK----MMFMGFIRL-GVWYNFFRAWN
M.musculus       106 VKRE--VEDFQPYFKG-EIFLDEKKKFYGP-ERRK----MMFMGLIRL-GVWYNSFRAWN
G.gallus         104 IGTE--VEDFQHYFQG-EIFLDEKRSFYGP-RKRK----MMLSGFFR--GVWQNFFRAWK
C.elegans        114 RG----ANEFKSWFSGGDVYLDTDRTFYGPNERWL----PVWMGFLRF-GTYSNVYKAKK
C.reinhardtii    113 HARSKDFVEVTG-FPAENLFADPNNDLYTALGLIKG-VGATFLSVETPLAIKRRMDSGNT
O.lucimarinus     92 LPAE--VEEFKRDFWPEHLYLDEDKAFFKAVGGGKLKKGSLSAFLNPFSRIWKHAGDAKK
O.tauri           86 LPAE--VEEFKRDYWPEHLYLDEDKAFFKAVGGGKLRKGSLTAFLNPFGRVYKHAGEAKK
T.pseudonana U1   95 GVDNQGLNEFATDYFPYPSYKDQARVFYTALGSGK----MSFNPLGLI-KLIRDSMKRIK
T.pseudonana U2  101 GVDDEGLKEFHNEHFTYPLFKDDGLVFYNEFFGKRKIKLTTYNPIKLY-KGYKDMTQRLK

H.sapiens        155 GG-FSGN--LEGEGFILGGVFVVGSGKQGILLEHREKEFGDKVNLLSVLEAAKMIKPQTL
M.musculus       157 GG-FSGN--LEGEGFILGGVFVIGSGKQGILLEHREKEFGDRVNPLSVLEAVKKIKLQTP
G.gallus         154 NG-YSGN--LEGEGFTLGGVYVIGAGRQGVLLEHREKEFGDKVSLPSVLEAAEKIKPQAS
C.elegans        165 AK-VEGN--MEGEGRLLGGVYLIAN--NDIVFTHLEKEWGDAADIKEVRAAVEKFSEKSK
C.reinhardtii    171 ADLMDWMPPKNEQGLQQGGMFLFDG--DRTVLTHYDKATSDHADLSALLGVAGQLAADCD
O.lucimarinus    150 AGVKEHN--LNGEGLIMGGMFVMKPGSQGVQYQFQERNFGDHAPIEDVLAAAKAASEASK
O.tauri          144 TGVTQHN--LNGEGLIFGGSFVMKAGSKGVQYQFQERNFGDHAPIEDILAAAKAAAAE--
T.pseudonana U1  150 E----------------SGVKSYNM--KG-------------------------------
T.pseudonana U2  160 EKKLDGN--YAGEGLIQGGIVIFDK--EGKVRYAYEEEIGKEIEMESIVDALKAIQNDA-

H.sapiens        212 ASEKK------------
M.musculus       214 ASGRS------------
G.gallus         211 -----------------
C.elegans        220 -----------------
C.reinhardtii    229 NACELPPPPPPPPARRF
O.lucimarinus    208 -----------------
O.tauri              -----------------
T.pseudonana U1      -----------------
T.pseudonana U2      -----------------
```

Selenoprotein Sep15

```
H.sapiens        1 MAAGPSGCLVPAFGLRLLLATVLQAVSAFGAEFSSEACRELGFS-SNLLCSSCD-LLGQF
M.musculus       1 MAAGQGGWLRPALGLRLLLATAFQAASALGAEFASEACRELGFS-SNLLCSSCD-LLGQF
D.rerio          1 -MAG-------EVYLLWLLP-LLQGLASYGAELSSEACRELGFS-SNLLCSSCE-LLGQF
O.lucimarinus    1 MRAR-------RR--SATLACVASSLVSVVRAHSAAECAALGFVSSECSVDACDNLRSKT
O.tauri          1 MRAR-------RS--AVVALGVTATFASTASALDASACAERGFIASECARSSCESLLRAT

                                               *
H.sapiens       59 NLLQLDPDCRGCCQEEAQFETKKLYAGAILEVCGUKLGRFPQVQAFVRSDKPKLFR---G
M.musculus      59 NLLPLDPVCRGCCQEEAQFETKKLYAGAILEVCGUKLGRFPQVQAFVRSDKPKLFR---G
D.rerio         50 SLNQLDLPCRQCCQEEAQLENRKLYPGAILEVCGUKLGRFPQVQAFVRSDKPKLFR---G
O.lucimarinus   52 ASVALYDECRACCAASNDAAAPVTYARALVRVCSUKLGRFPELKKFQTESLPALRQTR-D
O.tauri         52 SSRAAHDDCVACCAADVGRAR----TKASLRVCEUKLSRYANLRAFRDDALPALRRERFD

H.sapiens      116 LQIKYVRGSDPVLKLLDDNGNIAE--ELSILKWNTDSVEEFLSEKLERI------------
M.musculus     116 LQIKYVRGSDPVLKLLDDNGNIAE--ELSILKWNTDSVEEFLSEKLERI------------
D.rerio        107 LQIKYVRGSDPVLKLLDDNGNIAE--ELSILKWNTDSVEEFLSEKLERI------------
O.lucimarinus  111 VRFDLVQGMSPTLTMYEDAASDNNGIDVDVGSWSSDVIVEYLKSKLTPSPDASSRSSRDEL
O.tauri        108 VDFKLVKNAPPTLALSSDDDDDDD--VIDVGSWDADVIAEYVRSKVTPR--GASPADRSEL
```

Trx-fold protein

```
G.gallus          1 -----------------MSRP----------PQCP---------------VVQQLRPVSV
A.thaliana        1 MILVSESPMAILSLRSSSSLPLICSTISPLCSKPMLSQLPSNFSSSIASVPVTKLKSSYS
O.sativa          1 ----------MAAAAASTSLPVPRVSLPP-SARPAAAPRHGLLIP--GRRGCFRLRGSPA
O.tauri           1 -----------MATARTATTR-AWTATRARGARRG---------------DARGRAAARS
O.lucimarinus     1 -----------MAMRASLAMRSSASALELRCARRAR--------------VARARRAAMM
T.pseudonana      1 ----MRHHLVASLLTATAALP-SSSAFAPSAPIVAISPQVR-------HAASSLFLSTNA

G.gallus         19 AP--------------------RPELAELREAAYCLVVDAD-GSRIPFGALYR----RQK
A.thaliana       61 SSVSPISRPRVVSARAATESFTDYREDIGEILGDVSIFTAS-GQRVQFSDLWD--QKDGI
O.sativa         48 APAAAASGSPSVPS-----SSPEAGSGIGDALGGVAIYSAATGEPVLFRDLWD--QNEGM
O.tauri          34 TS--------TIPS--------DAPSDALEALRGVELRRASDGTAVTIPELVTGGGAKDT
O.lucimarinus    36 TSAASGTTRVNVPS--------DAPSDALEALRAVEVMRAIDGARVTVPEVV---GARGT
T.pseudonana     49 NDGDCGCGNAAIIS--GSPSNDARDINPYEALAQSTVYSLD-GTPIQMSTLLP---N-TV

                              *
G.gallus         54 AIVVFVRNFLCYTCKEYVEDLAKVPRSYLQEANVRLIVIGQSSYHHIKPFCSLTGYT---
O.sativa        101 AVVALLRHFGC-PCCWELASVLRDTKERFDSAGVKLIAVGVGTPDKARILAERLPFP--L
A.thaliana      118 AAVVLLRHFGC-VCCWELATALKEAKPRFDAAGVKLIAVGVGTPDKARILATRLPFP--M
O.tauri          78 TVVTWLRSFGUFFASERAVELAREAIPKLKEMDARLVVVSIGTLERAKDFARENAFP--I
O.lucimarinus    85 VVVQFLRSFGUFFASERAVELARDAIPTLDAAGVRLVVVSIGTLERAKDFSRENDFP--I
T.pseudonana    102 SIVTLGRSFGU-PLCQEQLLQYSSKRDLLLENDITIAFVSIGKPEIGRELCTHLGIEDGE

G.gallus        111 HEMYVDPQREIYKMLGMKRGEGNDVSVQSP-----HVKSSML--LG-SIRSMWRAMTSPA
A.thaliana      175 ECLYADPERKAYDVLGLYFGLGRTFFNPAS----TKVFSRFS-----EIREATKNYT-IE
O.sativa        158 DYLYADPERKAYDLLGLYFGIGRTFFNPAS----ASVFSRFD-----SLKEAVKNYT-IE
O.tauri         136 EYLYADEQSETYDALKLNKGVDATFMRKSTP---ESILARWKDNGAKDLLGVLKRWK-PW
O.lucimarinus   143 ELLYADAESATYEALKLRKGAKQTFMEKSTP---ESILKRWNKDGAKDLLGVLKRWK-PW
T.pseudonana    161 EWIFADPENETYDKLALNRGWNTMIRPETAFRFRDRIFGSSG--SLDQLFEVLGKWKDAV

G.gallus        163 FDFQG--DPAQQGGTLILGPGNEVHFLHHDRNRLDHVPINSVLQLARS-----------
A.thaliana      225 ATPEDRSSVLQQGGTFVFR-GKKLLYGRKDEGTGDHPSLDDVINVCCKATVA-------
O.sativa        208 ATPDDRASVLQQGGMFVFR-GKELIYARKDEGTGDHAPLDDVLNICCKAPAA-------
O.tauri         192 LPPRP-DQGYQQGGSFVFR-GSKTVYVSYDESTGAHAPLEDILRAARGD----------
O.lucimarinus   199 LPPRP-DQGYQQGGSFVFR-DGVATYVSYDVSTGAHAPLDDIFEAAGVSTSN-------
T.pseudonana    219 YIPPKIEQSTNHGGAFLFK-GSEVVFAHYDASPGTHADPFEVIDRAIQTARNAKETSVV
```

Selenoprotein W

```
                                                                                  *
H.sapiens              1 -----------------------------------MA-LAVRVVYCGAUGYKSKYLQLKK
M.musculus             1 -----------------------------------MA-LAVRVVYCGAUGYKPKYLQLKE
D.rerio                1 -----------------------------------MT-VKVHVVYCGGUGYRPKFIKLKT
C.reinhardtii W1       1 ----------------------------------MAP-VQVHVLYCGGUGYGSRYRSLEN
C.reinhardtii W2       1 ---------------------------------MAKTSIAAQVVMCGGUGYRGRYRSLVE
O.tauri                1 ----------------------------------MRA-IPIHIEYCEKUNYMPRVKMLVD
O.lucimarinus          1 MLAQRAQAFATGGATVASRARARATRGRTGRRAQTRAGIPIHIEYCEKUNYMPRVKMFID
O.lucimarinus Wb       1 ----------------------------------MNP-L-VHITYUGGUGYLRFATAIER

H.sapiens             25 KLEDE------FP-GRLDICGEGTPQATGFF-----EVMVAG------------------
M.musculus            25 KLEHE------FP-GCLDICGEGTPQVTGFF-----EVTVAG------------------
D.rerio               25 LLEDE------FP-NELEITGEGTPSTTGWL-----EVEVNG------------------
C.reinhardtii W1      26 AIRMK------FPNADIKFSFEATPQATGFF-----EVEVNG------------------
C.reinhardtii W2      28 AYRRR------FP-LWVPTSPTTQRCSLEAF-----EISVNG------------------
O.tauri               26 KTEAALPGKFEFTWNVESVLKETVPAGQGWYAENQAEKPIPGGEAALWRKGAFEVLRLDT
O.lucimarinus         61 KTNDACPGKFDFTYNVEEVLRATVPEGQGWYIENNAEKPIPGGESALWRKGAFEVLRLDT
O.lucimarinus Wb      25 AIVAK------FPRASV--AAEPTPTTSGAL-----EVKVAG------------------

H.sapiens             55 -KLIHSKKK-GDGYVDTESKFLKLVAA-IKAALAQG--
M.musculus            55 -KLVHSKKR-GDGYVDTESKFRKLVTA-IKAALAQCQ-
D.rerio               55 -KLVHSKKN-GDGFVDSDSKMQKIVTA-IEQAMGK---
C.reinhardtii W1      57 -ELVHSKKN-GGGHVDNQEKVERIFAK-IGEALAK---
C.reinhardtii W2      58 -GLVHSKEK-GMQFPYAPESWSGCT-------------
O.tauri               86 KELLYSKKAEGSHLVDGKGGPEGKLGKFIDEVLAKA--
O.lucimarinus        121 DELLYSKKADGSHLVDGKGGPEGKLGKFIEEVMKAAA-
O.lucimarinus Wb      54 -ELVHSKLTRGDGYVDDPVKIERILSA-VERALGENAR
```

Selenoprotein T

```
H.sapiens        1 -----------------MRLLLLLLVAASAMVRSEASANLG-----------GVPSKRLK
G.gallus         1 ------------------------------------------------------------
C.elegans        1 MRIHDELQKQDMSRFGVFIIGVLFFMSVCDVLRTEEHSHDENHVHEKDDFEAEFGDETDS
A.thaliana       1 -----------MDKTQLILLGLPIFLLCSDLFNLFTP---PPPK----------SQHQSP
O.sativa         1 -----------MDRVQLVLLGLPILLFCSDLVTLFGPEQLPTPQ-------PDLPPHPSP
C.reinhardtii    1 ---------MQGLHKGAILLGIVALFIGADCFGVMGG-----------------SKAPSQ
O.lucimarinus    1 -----------MLSRRAVGATRARATRARSRALVAASGIVLAIV-------ASDIYGAYA
O.tauri          1 -----------MTSR----ADRALFLRAAAVALVSCA--------------VADGWRTYA
T.pseudonana     1 ------------------------------------------------------------

                                                               *
H.sapiens       33 MQYATG---------------------------PLLKFQICVSUGYRRVFEEYMRVISQR
G.gallus         1 MAYATG---------------------------PLLKFQICVSUGYRRVFEEYMRVISQR
C.elegans       61 QSFSQGTEEDHIEVREQ-SSFVKPTAVHHAKDLPTLRIFYCVSCGYKQAFDQFTTFAKEK
A.thaliana      37 PSISETL--D-FPAQK--ST--------GVGYGNTVEINFCISCSYKGTAVSMKKMLESV
O.sativa        43 DAASDAVQPDDIAADAAASAQIAEPQVDGPASGTTVELKFCASCSYRGNAVTVKKMLETS
C.reinhardtii   35 ARVQSAMDPDGGLSLG--G---------------KLHVSFCNSUGMRGAFVQVMELARRR
O.lucimarinus   43 STATETAPSETFRAMSRGAG--------------KVVVGFCTSUSYRGGFNQLARAIENE
O.tauri         32 NALRGASPSEAFVRASE-SG--------------KVAIGFCTSUSYRGGFEQLKRALESE
T.pseudonana     1 --MHRPPPS--------------------------IRVFFCTQUGMKANFLRVREILINE

H.sapiens       66 YPD--IRIEGENYLPQPIYRHIASFLSVFKLVLIGLIIVGKDPFAFFG---MQAPSIWQW
G.gallus        34 YPD--IRIEGENYLPQPIYRHIASFLSVFKLVLIGLIIVGKDPFAFFG---MQAPSIWQW
C.elegans      120 YPN--MPIEGANFAPVLWKAYVAQALSFVKMAVLVLVLGGINPFERFG---LGYPQILQH
A.thaliana      84 FPG--LDVVLANYPAPAPKRILAKVVPVAQVGVIGLIMGGEQIFPMIG--IAQPPAWYHS
O.sativa       103 FPG--IHVVLENYPPPFPKRALSKAVPFLQVGAMATLMAGDQIFPRFG--MVPPP-WYYS
C.reinhardtii   78 YPG--LEVVGTPYPLPAWKVPVVKALQVVQFGLLGMCLAGDKVFAALG---VPVPAWYTQ
O.lucimarinus   89 F-G--MATTGGHWPPSATAVALGRAFSWVQIALWTATFFGERAATRVFGESAVVPAWLKS
O.tauri         77 FVG--VPVIGGHWPASAMGSAIARFTEYARAYAFVLILVGAAAFEWAG---MEMPSIVRS
T.pseudonana    33 FPGQWSSIEGENYPAPEWTKLAGSVVSALQIFGMVLVMVGDSIWSYIPGFRRGPPEVYYK

H.sapiens      121 -GQENKVYACMMVFFLSNMIENQCMSTGAFEITLNDVPVWSKLESGHLPSMQQLVQILDN
G.gallus        89 -GQENKVYACMMVFFLSNMIENQCMSTGAFEITLNDVPVWSKLESGHLPSMQQLVQILDN
C.elegans      175 -AHGNKMSSCMLVFMLGNLVEQSLISTGAFEVYLGNEQIWSKIESGRVPSPQEFMQLIDA
A.thaliana     140 -LRANRFGSMASTWLLGNFLQSFLQSSGAFEVSCNGELVFSKLKEGRFPGEIELRDLSSG
O.sativa       158 -LRANRFGTMATIWLFGNFAQSFLQSSGAFEVYCNGQLVFSKLSEQRFPSEFELRELIGN
C.reinhardtii  133 NVASNRFGAAMGVWFVGNMVVTNMQNTGAFEVFFNGDLIFSKLAEGRMPSVPELISPMQA
O.lucimarinus  146 -MSENKFQTAIMTFWVFNIVSANVLNTGAFEVFYDGELVSSKLQSGKLPRVDTIFEGIRA
O.tauri        132 -MSENKMHSALVAFWVSNMIATNCLNTGAFEVYFDGKLVTSKLASKTLPRIDAVFDGIRS
T.pseudonana    93 -LKDNPALALIGVFLIIPSYIQSFANTGAFEIMLDGKVIFSKLELGRMPNVAEIIKAVES

H.sapiens      180 EMKLN----VHMDSIPHHRS-------------------------
G.gallus       148 EMKLN----VHMESMPHHRS-------------------------
C.elegans      234 QLAVLGKAPVNTESFGEFQQTV-----------------------
A.thaliana     199 TMTKP----FVTGSY------------------------------
O.sativa       217 RLPDS----QFGKNLEKVWS-------------------------
C.reinhardtii  193 FFEGPAG--LHVGGAGASRPGLTGAGMGHGPELSGVGAAAVGLTG
O.lucimarinus  205 IQGK-----------------------------------------
O.tauri        191 IRGK-----------------------------------------
T.pseudonana   152 AGLHR-----GR---------------------------------
```

Thioredoxin reductase

```
H.sapiens         1 ------------------------------------------------------------
M.musculus        1 ------------------------------------------------------------
G.gallus          1 MPPPGQTQLPDWDGLKLRVRTLIATHRVMIFSKSYCPYCHRVRRRRGASLLLGPTQTLPF
C.reinhardtii     1 ------------------------------------------------------------
O.lucimarinus     1 ------------------------------------------------------------
O.tauri           1 ------------------------------------------------------------
T.pseudonana      1 ------------------------------------------------------------

H.sapiens         1 ------------------------------------------------------------
M.musculus        1 ------------------------------------------------------------
G.gallus         61 LPGRSRPLVRPPGEAGGSGLGSPRGRGARADPAGTAPCEWWAAICGVVSFPPVGAHSIEV
C.reinhardtii     1 ------------------------------------------------------------
O.lucimarinus     1 ------------------------------------------------------------
O.tauri           1 ------------------------------------------------------------
T.pseudonana      1 ------------------------------------------------------------

H.sapiens         1 ------------------------------------------------------------
M.musculus        1 ------------------------------------------------------------
G.gallus        121 MRGRSWVLGGFFIFQGRRVKELFSSLGVQYYALELDVTDDGPSIQQVLAELTNQRTVPNV
C.reinhardtii     1 ------------------------------------------------------------
O.lucimarinus     1 ------------------------------------------------------------
O.tauri           1 ------------------------------------------------------------
T.pseudonana      1 ------------------------------------------------------------

H.sapiens         1 ------------------------MNGP-E--DLPKSYDYDLIIIGGGSGGLAAAKEAAQ
M.musculus        1 ------------------------MNGS-K--DPPGSYDFDLIIIGGGSGGLAAAKEAAK
G.gallus        181 FINGKHIGGCDATYKAYENGTLQRILGD-V--KDAETYDYDLIVIGGGSGGLACSKEAAT
C.reinhardtii     1 ------------------------MAAAGAPAEGASAYEYDLVVIGGGSGGLACAKEAAK
O.lucimarinus     1 ------------------------MTETSC--KGDHGHEYDVVVIGGGSGGLAAAKEAAK
O.tauri           1 ------------------------MSET----KGDHGYEYDVVVIGGGSGGLAAAKEAAK
T.pseudonana      1 ------------------------------------PYEYDLLVLGGGSGGLAASKEAAA

H.sapiens        34 YGKKVMVLDFVTPTPLGTRWGLGGTCVNVGCIPKKLMHQAALLG-QALQDSRNYGWKVEE
M.musculus       34 FDKKVLVLDFVTPTPLGTRWGLGGTCVNVGCIPKKLMHQAALLG-QALKDSRNYGWKVED
G.gallus        238 LGKKVMVLDYVVPTPLGTSWGLGGTCVNVGCIPKKLMHQAALLG-QALKDSRAYGWQYDE
C.reinhardtii    37 LGKKVCLLDYVVPSPAGTSWGLGGTCVNVGCIPKKLMHNAGLLG-EGFSDARGYGWKLPE
O.lucimarinus    35 HGAKTMCLDFVKPSPAGTTWGLGGTCVNVGCIPKKLMHQAGILG-ESFSDAREYGWKLAS
O.tauri          33 HGAKTACLDFVKPSPAGTTWGLGGTCVNVGCIPKKLMHQAGLLG-ESFSDAREYGWKLAS
T.pseudonana     25 HGARVAVLDYVKPSPAGSTWGLGGTCVNVGCIPKKLMHTAALLNYQQKVDQPHYGINVSE

H.sapiens        93 T--------------VKHDWDRMIEAVQNHIGSLNWGYRVALREKKVVYENAYGQFIGPH
M.musculus       93 T--------------VKHDWEKMTESVQSHIGSLNWGYRVALREKKVVYENAYGRFIGPH
G.gallus        297 Q--------------VKHNWEIMVEAVQNYIGSLNWGYRLSLREKSVTYQNSYGEFVEPH
C.reinhardtii    96 K--------------IEMNWEDLVMGVQNHIGSLNWGYRVALREASVKYLNAKGSFVDAH
O.lucimarinus    94 ---------------EGHDWGKMVEQIQNHIGSLNFGYRTTLREKNVTYVNAYGRFKDKN
O.tauri          92 ---------------EGHDWPKMVEQIQNHIGSLNFGYRTTLREKNVTYINAYGKFKDAH
T.pseudonana     85 SQTEEWMGMSQDNADAPHSWGILKNNVQNHIRGLNFKYRVDLREKEVTYLNMLGKFKDAH

H.sapiens       139 RIKATNNKGKEKIYSAERFLIATGERPRYLGIPGDKEYCISSDDLFSLPYCPGKTLVVGA
M.musculus      139 RIVATNNKGKEKIYSAERFLIATGERPRYLGIPGDKEYCISSDDLFSLPYCPGKTLVVGA
G.gallus        343 KIKATNRKGQVTYHTAETFVLATGERPRYLGIPGDKEYCITSDDLFSLPYCPGKTLVVGA
C.reinhardtii   142 TVEAVERNGTKHTLTAERVVIAVGGRPKYLGVPGDKELCITSDDIFSRATPPGKTLVVGA
O.lucimarinus   139 TIIATKKNGQEQVITTDKVVVAVGGRPSYPDAPGAKECCITSDDIFSKPEAPGKTLCVGA
O.tauri         137 TIVATKKNGTEQIITTDKVVIAVGGRPAYPDAPGAKECCITSDDIFSKPDAPGKTLCVGA
T.pseudonana    145 TVETVDKKGNVGSITASRFLIAVGGRPSPLDCEGG-ELAISSDDVFSLENDPGKVLCVGA

H.sapiens       199 SYVALECAGFLAGIGLDVTVMVRSILLRGFDQDMANKIGEHMEEHGIKFIRQFVPIKVEQ
M.musculus      199 SYVALECAGFLAGIGLDVTVMVRSILLRGFDQDMANKIGEHMEEHGIKFIRQFVPTKIEQ
G.gallus        403 SYVALECAGFLAGLGLDVTVMVRSILLRGFDQEMAEKIGAHMETHGVTFIRKFVPTQVER
C.reinhardtii   202 SYIALECAGFLRALGYEVAVMARSIFLRGFDQEIAELIGKDMERRGVRMIKPAVPTAFER
O.lucimarinus   199 SYISLETAGFLTALGFDTAVAIRSIPLRGFDQEVAEKIVKYMGEHGTRFLRDSQPTVFEK
O.tauri         197 SYISLETAGFLTALGFDTSVAIRSIPLRGFDQEVAEKICKYMGKHGTRFLRDSQPSQFEK
T.pseudonana    204 SYISLECAGFLKGIGKDVTVAVRSILLRGFDRECADLIGEHMRHEGIVFKEEVVPKKLVK
```

```
H.sapiens       259 IEAGTPGRLRVVAQSTNSEEIIEGEYNTVMLAIGRDACTRKIGLETVGVKINEKTGKIPV
M.musculus      259 IEAGTPGRLRVTAQSTNSEETIEGEFNTVLLAVGRDSCTRTIGLETVGVKINEKTGKIPV
G.gallus        463 LEDGTPGRLKVTAKSTEGPEFFEGEYNTVLIAIGRDACTRNIGLQTIGVKINEKNGKVPV
C.reinhardtii   262 DGE----QIKCTFKNLDFGVEMSESFDTVLLAVGRDACTFDLGLEKVGVTYDKSSGKIPV
O.lucimarinus   259 QEDG---KIKVTFENTMFGNTFEETFDTVVCAVGRDAVTEGLDLPAAGVEFNPKNGKIAC
O.tauri         257 QEDG---KIKVTFENTMFGNTFEETFDTVVCAVGRDAVTEGLDLPAAGVEFNAKNGKIPC
T.pseudonana    264 TEGG---RIAVTFSNGD-----VEEYDTVLAAIGRTGDTSKLGLENVGIDVNPKNAKIPA

H.sapiens       319 TDEEQTNVPYIYAIGDILEDKVELTPVAIQAGRLLAQRLYAG--STVKCDYENVPTTVFT
M.musculus      319 TDEEQTNVPYIYAIGDILEGKLELTPVAIQAGRLLAQRLYGG--SNVKCDYDNVPTTVFT
G.gallus        523 NDEERTNVPYVYAIGDILDGKLELTPVAIQAGKLLARRLYGG--SSTKCDYINVPTTVFT
C.reinhardtii   318 TA-EQTNVPSIYAIGDVLESRQELTPVAIKAGIRLARRLYAG--ATLQMDYDAVPTTVFT
O.lucimarinus   316 VD-EQTNVDNIYAIGDVLDTRQELTPVAIKAGVRLMRRVFADTPYKEKMNYDLVPTTVFT
O.tauri         314 VD-EQTNVPNIYAIGDVLDTRQELTPVAIKAGVRLMRRVFADTPYKEKMNYDLVPTTVFT
T.pseudonana    316 KL-EQTCTPNIYVIGDVMDGCPELTPVAIHAGKMLSRRLFAG--STAPMDYRNVCTTVFT

H.sapiens       377 PLEYGACGLSEEKAVEKFGEENIEVYHSYFWPLEWTI--------PSRDNNKCYAKIICN
M.musculus      377 PLEYGCCGLSEEKAVEKFGEENIEVYHSFFWPLEWTV--------PSRDNNKCYAKIICN
G.gallus        581 PLEYGSCGLAEEKAIEEYGKQNLEVYHSLFWPLEWTV--------PGRDNNTCYAKIICN
C.reinhardtii   375 PLEYGCVGYSEEAATVKYGADNIEVYVSYLKPLEWTMNHEEHNGEPVRADNSVFVKLITN
O.lucimarinus   375 PLEYGTIGMSEELAVETYGADNVECYVSYFKPLEWTLNHEEHKGVPVRGDNACYVKLITN
O.tauri         373 PLEYGTIGMSEELAVETYGADNVECYISYFKPLEWTVNHEEHNGVPVRDDNACFVKLITN
T.pseudonana    373 PLEYGTVGYSEDDAIAEFGKENVEVYHKYFIPLEWSLS-------PSRSESQGFCKAIVY

H.sapiens       429 TKDNERVVGFHVLGPNAGEVTQGFAAALKCGLTKKQLDSTIGIHPVCAEVFTTLSVTKRS
M.musculus      429 LKDDERVVGFHVLGPNAGEVTQGFAAALKCGLTKQQLDSTIGIHPVCAEIFTTLSVTKRS
G.gallus        633 KLDGNRVVGFHVLGPNAGEVTQGFAAAIKCGLTKELLDETIGIHPTCAEVFTTMDITKSS
C.reinhardtii   435 TADNERVVGAHYLGPNAGEIIQGVAVAVKANATKADFDDCIGIHPTVAEEFTILEVTKRS
O.lucimarinus   435 LADDERVVGFHYLGPNAGEVTQGYAVAMKMGATKKDFDETVGIHPTVSEEFTILEITKRS
O.tauri         433 LADDERVVGFHYLGPNAGEVTQGYAVAMKMGATKRDFDETVGIHPTVSEEFTILEITKRS
T.pseudonana    426 KATR-KVLGLHYLGPNAGEVMQGFGTAMKLGCKFEDITETVGIHPTTAEELTTLSITKAS

                             *
H.sapiens       489 GASILQAGCUG
M.musculus      489 GGDILQSGCUG
G.gallus        693 GQDITQRGCUG
C.reinhardtii   495 GKSALKKGCUG
O.lucimarinus   495 GIDPTKKGCUG
O.tauri         493 GVDPSKRGCUG
T.pseudonana    485 GADAKASGCUG
```

Selenoprotein S

```
H.sapiens       1 MERQEE----SLSARPALETEGLRFLHTTVGSLLATYGWYIVFSCILLYVVFQKLSARLR
M.musculus      1 MDRDEE----PLSARPALETESLRFLHVTVGSLLASYGWYILFSCILLYIVIQRLSLRLR
G.gallus        1 MELGDRGGAGPGPGKPALEREGLELLQHTVGALLSSYGWYILLACVAIYLIVQKISPYLR
O.lucimarinus   1 -----------------MWTAYARVASVVENALGINFHAFVACVTLVIALTYPKLAKSVS
O.tauri         1 ----------------MMFGIDADVARIVS--IAL---------ALVAAYALTRFMASTS

H.sapiens      57 ALRQRQLDRAAAAVEPDVVVKRQEALAAARLKMQEELNAQVEKHKEKLKQLEEEKRRQKI
M.musculus     57 ALRQRQLDQAETVLEPDVVVKRQEALAAARLRMQEDLNAQVEKHKEKLRQLEEEKRRQKI
G.gallus       61 MRPSSQQGATGAAVEPDMVVRRQEALLASRLRMQEELNAQAERYKEKQRQLEEQKRRQKI
O.lucimarinus  44 DRLDSRAMNDRERLER-YQRNRQRALEKR----EEELRRAASETKGGKAELD---RAAAL
O.tauri        34 IKAERRAEEERQKLER-YRAQRQRALERR----EEELRAASFERENGKDTTD---RETAL

H.sapiens     117 EMWDSMQEGKSYKGNAKKPQEEDSPGPSTSSVLKR-KSDRKPLRGGGYNPLSGEGGGACS
M.musculus    117 EMWDSMQEGRSYKRNSGRPQEEDGPGPSTSSVIPKGKSDKKPLRGGGYNPLTGEGGGTCS
G.gallus      121 EMWESMQEGKSYKGNLKLSQQEAESGASTSSAVPKSKPNKKPLRGGGYNPLSGEGGGTCS
O.lucimarinus  96 EAKLAEIDAKAARLGLTANGKGRKLGDSGPSRPE-------------WNPLMG-GGASRG
O.tauri        86 EKKLAELDAKAARLGVLAT-RGRQLGSS-STRSE-------------WNPLTG-SGSSSG

                                     *
H.sapiens     176 WRPGRRGPSS-GGUG-
M.musculus    177 WRPGRRGPSS-GGUN-
G.gallus      181 WRPGRRGPSA-GGUG-
O.lucimarinus 142 YRPTGRPRPGGGGUGR
O.tauri       130 YRPQRRAPPG-GGUGR
```

Selenoprotein O

```
H.sapiens         1 ----------------MAVYRAALGASLAAARLLP--LGRCSP----------------S
M.musculus        1 ----------------MASVRAAVGASLAVARTRPRCVGLALP----------------S
A.thaliana        1 --MLLRVCCPSSFFYLRPLHLFSSTAKVPFCPSLPRQFRLSPSRSSSFRRMESSPASSSS
O.sativa          1 ----MPPHLSSSLFSSSPLPTALLSSLTRRARALLP-LPLPLPAASTRRRLPLRRGPLLT
O.lucimarinus     1 MLARDASARVAVPHCGTRMFERGALARLGRRAVERARAPALRPERGAAGAAHARRRRARA
O.tauri           1 ------------------------------------------------------------
P.haitanensis     1 ------------------------------------------------------------

H.sapiens        27 PAPRSTLSGAAMEPAPRWLAGLRFDN-----RALRALPVEA----PPPGPEGAPSAPRPV
M.musculus       29 SAPRSAWA-AAMEPTPRWLAGLRFDN-----RALRELPVET----PPPGPEDSLATPRPV
A.thaliana       59 PTPVTDSSADSLAKDLQNQSLGAVDEGVKIKKKLEDFNWDHSFVKELPGDPRTDVISREV
O.sativa         56 RGIMASAAAEAPAPSP--SPTAAANS--RPRRVLEELSWDDSFVRELPGDPRSDAIPREV
O.lucimarinus    61 TTTTTTMTSKPLDLHDSFEDAARR--------AGAQTWIESLDEDPESSARAPNRTSREV
O.tauri           1 ------MTSTTTCAYPTFDDALRATT------SSAQTWTRDLDVDPDWERNAPNRRSREV
P.haitanensis     1 ------------------------------------------------------------

H.sapiens        78 PGACFTRVQP-TPLRQPRLVALSEPALALLGLGAPP--AREAEAEAALFFSGNAL-----
M.musculus       79 PGACFSRARP-APLRRPRLVALSEPALALLGLEASE--EAEVEAEAALFFSGNAL-----
A.thaliana      119 LHACYSKVSPSVEVDDPQLVAWSVSVAELLDLDP----KEFERPDFPLMLSGAKP-----
O.sativa        112 LHACYTKVSPSAPVDNPKLVAWSQSVADILDLDH----KEFERPDFPQLFSGANP-----
O.lucimarinus   113 RSGHYVEVEP-EALANPRARLASTTCAEAIGFKIAR-ECENLEDGFVKYFSGDVGGARET
O.tauri          49 RSGHYVPVTP-ERLEAPRGRLASASCAREIGFALSGEDLNNPRSTFVRYFSGDVEGIDGG
P.haitanensis     1 ------------------------------------------------------------

H.sapiens       130 ----LPGAEPAAHCYCGHQFG-----QFAGQLGDGAAMYLGEVCT-ATGERWELQLKGAG
M.musculus      131 ----LPGTEPAAHCYCGHQFG-----QFAGQLGDGAAMYLGEVCT-AAGERWELQLKGAG
A.thaliana      170 ----LPGAMSYAQCYGGHQFG-----MWAGQLGDGRAITLGEVLN-SKGERWELQLKGAG
O.sativa        163 ----LVGSSPYAQCYGGHQFG-----SWAGQLGDGRAITLGEVIN-SRGERWELQLKGCG
O.lucimarinus   171 T--MRTWATPYALSIMGQRMTSNCPFGNGNGYGDGRAISVGEMVNPVTGQRYELQLKGGG
O.tauri         108 IGGMKTWATPYALSIMGSRMTSNCPFGNGNGYGDGRAISVGEMINPVTGARHELQLKGGG
P.haitanensis     1 ------------------------------------------------------------

H.sapiens       180 PTPFSRQADGRKVLRSSIREFLCSEAMFHLGVPTTRAGACVTSESTVVRDVFYDGNP---
M.musculus      181 PTPFSRQADGRKVLRSSIREFLCSEAMFHLGIPTTRAGACVTSESTVMRDVFYDGNP---
A.thaliana      220 RTPYSRFADGLAVLRSSIREFLCSETMHCLGIPTTRALCLLTTGQNVTRDMFYDGNP---
O.sativa        213 KTPYSRFADGLAVLRSSIREFLCSEAMHGLGIPTTRALCLVETGKSVVRDMFYDGNS---
O.lucimarinus   229 RTPFCRGADGRAVLRSSIREFLASEAMHALGVDTTRALCLIESVRGTTARRPWYSPTSDE
O.tauri         168 RTPFCRGADGRAVLRSSIREFLASEAMHALGVDTTRALCLIESERGSTARR---------
P.haitanensis     1 ------------------------------------------------------------

H.sapiens       237 ---------------------------------------KYEQCTVVLRVASTFIRFGSF
M.musculus      238 ---------------------------------------KYEKCTVVLRIAPTFIRFGSF
A.thaliana      277 ---------------------------------------KEEPGAIVCRVSQSFLRFGSY
O.sativa        270 ---------------------------------------KEEPGAIVCRVAPSFLRFGSY
O.lucimarinus   289 EHAKRVPTVDDPRLKDYPPEQRVEIVEMLKQQKRDPDIMIQEPCAITTRVAPSFMRIGHI
O.tauri         219 ------PTIRD----------------------------CAQPCAITTRVAPSFMRIGHI
P.haitanensis     1 ------------------------------------------------------------

H.sapiens       258 EIFKSADEHTGRAGPSVGRNDIRVQLLDYVISSFYPEIQAAHASDS--------------
M.musculus      259 EIFKPPDEHTGRAGPSVGRDDIRVQLLDYVISSFYPEIQAAHTCDTD-------------
A.thaliana      298 QIHASRGKED---------LDIVRKLADYAIKHHFPHIESMDRSDSLSFKTGDEDDSVVD
O.sativa        291 QIHATRDKED---------LEIVRHLADYTIRHHYPHLENIKKSEGLSFEAAIGDSPAID
O.lucimarinus   349 DLFSRRATAPR---ATALQKEQLKKIIRHAAFREFPETIEEHGEDM--------------
O.tauri         245 DLFSRRATASG---ATALQKEQLEKIVRHAAFREFPETIEQHGDDT--------------
P.haitanensis     1 ------------------------------------------------------------

H.sapiens       304 --VQRNAAFFREVTRRTARMVAEWQCVGFCHGVLNTDNMSILGLTIDYGPFGFLDRYDPD
M.musculus      306 -NIQRNAAFFREVTQRTARMVAEWQCVGFCHGVLNTDNMSIVGLTIDYGPFGFLDRYDPD
A.thaliana      349 LTSNKYAAWIVEIAERTATLVARWQGVGFTHGVLNTDNMSILGQTIDYGPFGFLDAFDPS
O.sativa        342 LTSNKYAAWAVEVAERTAFLIARWQGVGFTHGVLNTDNMSVLGLTIDYGPFGFLDAFDPS
O.lucimarinus   392 --AKVTRSMLEKSGKKIAKMVAGWIRVGFCQGNFNADNCLVGGRTMDYGPFGFMDKYDPS
O.tauri         288 --AAMTRAMLEKSGAKIAKMVAGWLRVGFAQGNFNADNCLVGGRTMDYGPFGFMDRYDPG
P.haitanensis     1 ------------------------------------------------------------
```

```
H.sapiens     362 HVCNASDNTG-RYAYSKQPEVCRWNLRKLAEALQPELPLELGEA-ILAEEFDAEFQRHYL
M.musculus    365 HICNASDNAG-RYTYSKQPQVCKWNLQKLAEALEPELPLALAEA-ILKEEFDTEFQRHYL
A.thaliana    409 YTPNTTDLPGRRYCFANQPDIGLWNIAQFSKTLAVAQLINQKEANYAMERYGDKFMDEYQ
O.sativa      402 YTPNTTDLPGKRYCFANQPDVGLWNIAQFTSPLTAAELISKDEANYVMERYGTKFMDEYQ
O.lucimarinus 450 FAKWTGSGDH--FAFMAQPKAGLTNFAVLAVSCAPLLAGGSDEATELVREMEATFENELN
O.tauri       346 FAKWTGSGEH--FAFMAQPDAGITNFAVLAVSCAPLLKGGSKEAQDIVRDAQPVFARAVD
P.haitanensis   1 ------------------------------------------------------------

H.sapiens     420 QKMRRKLGLVQVEL-----EEDGALVSKLLETMHLTGADFTNTFYLLSSFP---VELESP
M.musculus    423 QKMRKKLGLIRVEK-----EEDGTLVAKLLETMHLTGADFTNTFCVLSSFP---ADLSDS
A.thaliana    469 AIMSKKLGLTKYN------KE---VISKLLNNMSVDKVDYTNFFRLLANVK---ANPNTP
O.sativa      462 SIMTRKLGLPKYN------KQ---LIGKLLNNLAVDKVDYTNFFRLLSNVK---ADHNIP
O.lucimarinus 508 DVFRAKLGFAPNEDSVRVARDLFRSENGLEGLMYESQADWTVTWRRLAEC----AEVADE
O.tauri       404 DTFRTKLGFAS-ESSMDVARELFRAEDGLERLMYEDRADWTMTWRQLAEC----AEVSDA
P.haitanensis   1 AHYRANWVPGP----------PSR--IVLSSLLEKTQSDYTIFWRQLAQFNDKFFDKSTP

H.sapiens     472 GLAEFLARLMEQCASLEELRLAFRPQMDPRQLSMMLMLAQSNPQLFALMGTRAGIARELE
M.musculus    475 --AEFLSRLTSQCASLEELRLAFRPQMDPRQLSMMLMLAQSNPQLFALIGTQANVTKELE
A.thaliana    517 ----------------------------------------ENELLKPLKAVLLDIGKERK
O.sativa      510 ----------------------------------------EKELLVPLKAALLDIGPERK
O.lucimarinus 564 --------------------------------------SDDEALLAPLLETCFYG-NSMN
O.tauri       459 --------------------------------------NDDAALLAPLLSRCFYGGGIMC
P.haitanensis  49 --------------------------------------VNEEDVLSVFQNTFYEPLSDSL

H.sapiens     532 RVEQQSRLEQLSAAELQSRNQGHWADWLQAYRARLDKDLEGAGDAAAWQAEHVRVMHANN
M.musculus    533 RVEHQSRLEQLSPSDLQRKNRDHWEAWLQEYRDRLDKEKEGVGDTAAWQAERVRVMRANN
A.thaliana    537 ---------------------EAWIKWMRSY---IQ-E---VGGSEVSDEERKARMDSVN
O.sativa      530 ---------------------EAWISWVQTY---IE-E---LVSSGVPDEERKAAMNSVN
O.lucimarinus 585 --------------------DERKASWCA-FIRRWRDA---LKASGTSLADAAKRMRSEN
O.tauri       481 --------------------DGRKTKWLS-FIRRWRTA---LEASGTSLADAAKAMRAAN
P.haitanensis  71 --------------------KGKFVDWLNRYVGHLKTD---LDQSGLSIDETSTNMRKIS

H.sapiens     592 PKYVLRNYIAQNAIEAAERGDFSEVRRVLKLLETPYHCEAGAATDAEATEADGADGRQRS
M.musculus    593 PKYVLRNYIAQKAIEAAENGDFSEVRRVLKLLESPYHSEE-EATGPEAVARSTEE--QSS
A.thaliana    569 PKYILRNYLCQSAIDAAEQGDFSEVNNLIRLMKRPYEEQP----------------GMEK
O.sativa      562 PKYVLRNYLCQTAIDAAEQGDYDEVRRLLKVMEHPYDEQP----------------GMEK
O.lucimarinus 621 PKYVLREHLLVDAYTKASDGDFSLAEELFELTQHPYGGEG------------DDAKYDAK
O.tauri       517 PKYVLREHLLVEAYTKASSGDFSMVNELFALTQHPYGGDG------------DTAEFDAK
P.haitanensis 108 PKYIPREWMLVRTYTDAMKGDYALLKELYELFLNPYD---------------EQPEYEEK

                                       *
H.sapiens     652 YSSKPPLWAAE-------LCVTUSS
M.musculus    650 YSNRPPLWAAE-------LCVTUSS
A.thaliana    613 YARLPPAWAYR----PGVCMLSCSS
O.sativa      606 YARLPPAWAYR----PGVCMLSCSS
O.lucimarinus 669 YFVKAPEEALT---SGGVAFMSUSS
O.tauri       565 YYVKAPEEALT---SGGTAFMSUSS
P.haitanensis 153 YYKKAPIEVYEGFGLGGTAFMTUSS
```

Selenoprotein K

```
D.melanogaster     1 ----MVY---------IDHNGRVWEKR---PWDWRRIVELFVGIWFAIKQLFLTFLAPFTG
D.pseudoobscura    1 ----MVY---------IDRNGRVCEKR---PWDWPRIVEIFLRIWYIIKQLFLTFLAPFDG
C.reinhardtii      1 ----MPY---------ISRTGTVQERRS--PWRLSIVVEFFMGVWGAISTFFMTMVSPQAH
H.sapiens          1 ----MVY---------IS-NGQVLDSRSQSPWRLSLITDFFWGIAEFVVLFFKTLLQQDVK
M.musculus         1 ----MVY---------IS-NGQVLDSRNQSPWRVSFLTDFFWGIAEFVVFFFKTLLQQDVK
G.gallus           1 ----MVY---------IS-NGQVLDNRSRAPWSLSAITDFFWSIADFVVMFFQSIIQPDLR
O.lucimarinus      1 ----MAY---------VARDGAV---AAR-PFS---LVAALWGALDVVDAFFRTLLVEGHA
O.tauri            1 ----MTHPWSHFRCSIDRHRATPDDAHG-PFS---LSGACWHLVDVADAFVRTLVIDGYA
D.discoideum       1 MPPKPTY---------VS-GGSVTQTGRS-KWRLSYIPEFIWGILNQITFFFSTLIGGTVE

D.melanogaster    46 ---NNNQANPRRGNG--WGGGGGWGGGGGGGGGG-GGRPGSGSG--GLRPNRRIGRIQP
D.pseudoobscura   46 ---KSNNDN-RRG------GGSGWGGGGGGGGGG-GGGGGSGGGNEGLRPNRRIGRIQN
C.reinhardtii     47 ---EAYLKQQVKK----KDPPRTTGGPRIAGLDNI-GG--GGGSH---LTP----G----
H.sapiens         48 K-RRSYGNSSDSR----YDDGRGPPGNPPRRMGRI-NHLRG-PSP-----P---------
M.musculus        48 K-RRGYGSSSDSR----YDDGRGPPGNPPRRMGRI-SHLRG-PSP-----P---------
G.gallus          48 --RRGYTSSSYLG----QSDGRGPPGNPRRRMGRI-NHWGGGPSP-----P---------
O.lucimarinus     42 ---DAYARR-TT-----TARATTPGRVAATTARG--AAVRGFANVR-TIDHA--------
O.tauri           53 ---ETYARG-TNG----LSRASTASRAPVRRPGGG-GDVRGFANVR-TIDHS--------
D.discoideum      51 PRRRPNNQGGGRRLAGFDGNGNVTGGSGVGGSGPSKGPDNGSNNRRGDMKN---------

                                    *
D.melanogaster    98 TMSCNMPAG-GGUG--
D.pseudoobscura   95 SMSCNTPTGGGGUG--
C.reinhardtii     86 ---C---AG-GGUG--
H.sapiens         87 ------PMA-GGUGR-
M.musculus        87 ------PMA-GGUGR-
G.gallus          87 ------PMAGGGUGR-
O.lucimarinus     82 -----APTA-GGUGR-
O.tauri           95 -----APVG-GGUGR-
D.discoideum     102 --ILACNSASGSUGPK
```

Selenophosphate synthetase

```
                                                                                *
H.sapiens          1 MAEASATGACGEAMAAAEGSSGPAGLTLGRSFSNYRPFEPQALGLSPSWRLTGFSGMKGU
D.rerio            1 MSGSEPP--------SGD--SGGAGGYAVFYPAGYQALNPEEHGLDRGFRLTAFSDMKGU
D.discoideum       1 -----------------------------------MSISIKDKKEELLCRLTDFTKLKGU
T.pseudonana       1 -------------------------------------FDPTTLGLPPNWSLTSFSALKGU
C.elegans          1 ---------------------------MNRIERILEGFDPVSNGLDEDFVLTKLTGMKGC
D.pseudoobscura    1 ------------------------------------MFRPEKHGLEPDFQLTNFSTLRGU
D.melanogaster     1 ------------------------------------MFQPEKHGLEPDFQLTKFTTHTGU

H.sapiens         61 GCKVPQEALLKLLAGLTRPDVRPPLGRGLVGGQEEASQEAGLPAGAGPSPTFPALGIGMD
D.rerio           51 GCKVPQETLLKLLQGLE-PDRPPGEDGGLGTGVGDETADFGLVSAAQG----PRLGIGMD
D.discoideum      26 GCKVPQAELLSLLD-------------GIGEG------------------------IGYD
T.pseudonana      24 ACKVPQTQLQSMLSTL-----------GLGDGQN----MG----TANG-------KIALD
C.elegans         34 GCKVPRNVLLQLLQTFK---------TDLVINND----E---------------VDIGLD
D.pseudoobscura   25 GCKIPQDVLTKYLRG-----------TEIVHSNE---NEE------------HHIGAGMD
D.melanogaster    25 SCKIPQKVLEKYLRG-----------TEIENKN----NDG------------YLIGSGMD

H.sapiens        121 SCVIPLR---HG-GLSLVQTTDFFYPLVEDPYMMGRIACANVLSDLYAMGITECDNMLML
D.rerio          106 SCVIPLR---HG-GLSLVQTTDFFYPLVEDPYMMGRIACANVLSDLYAMGITECDNMLML
D.discoideum      49 CSISQTK---FP-DIVMIQTTDFFFPLVDDPYFQGKIACANVLSDLYSFGIEDCDNMLML
T.pseudonana      58 CSVRKTK---QG--HVCISTTDFFFPLVDSPYLQGRIGAANVLSDLYAEGVGECDFMLML
C.elegans         66 SCVIPLR---HP-GLRLVQTTDFFYPLIDDPYIMGRVTCANVLSDLYAMGVSECDNMLML
D.pseudoobscura   59 CAVIPLNSTRHGGEYLLAQTVDFFYPLVNDPEALGRITLANVLSDLYAVGVTDIDSIEMI
D.melanogaster    58 CAVIPLK--RHK-DYLLIQTVDFFYPMVNDPELLGRIALANVLSDVYAVGVTQFDTVEMI

H.sapiens        177 LSVSQSMSEEEREKVTPLMVKGFRDAAEEGG---TAVTGGQTVVNPWIIIGGVATVVCQP
D.rerio          162 LSVSQKMNEKERDLVLPLMMKGFRDAAEEGG---TSVTGGQTVINPWIIIGGVASVVCQP
D.discoideum     105 LACSTDMTAEQRQWSSKLMIQGFNDQAICAG---SKVSGGQTVKNPWPIVGGVATSILKT
T.pseudonana     113 LAACRDMVDEERNICTREMVRGFNDACIEAG---TTVTGGQTVLNPWPVIGGVATTICSE
C.elegans        122 LAVAIDLNEKQRDIVVPLFIQGFKDAADEAG---TKIRGGQTVRCPWLLLGGVATSVAHE
D.pseudoobscura  119 ISVSTEFTSKQRDVVTSLIIQGFRKALATNGYNQVHLKLRELKLNPWCIVGGVATSVCLK
D.melanogaster   115 VSTSTSFSEKERDVVIGLVMKGFQNSLKANGYRNTPLIIRQLKINPWCIIGGIATSVCRS

H.sapiens        234 NEFIMPDSAVVGDVLVLTKPLGTQVAVNAHQWLDNPE-RWNKVK--MVVSREEVELAYQE
D.rerio          219 NDFILPDGAVPGDVLVLTKPLGTQVAVNAHQWLDIPE-KWNKIK--LVISREEVEQAYQE
D.discoideum     162 NEFIKPVNAVPGDVLVLTKPLGTQVCVNFHQWLSKPE-RWEKIN--TITNAEECEQVFNY
T.pseudonana     170 GEYVKSDGAQVGDVVVLTKPLGTQVAVNVHEWRVRQTPKWKELTKRNVLSKEEAEDMMHS
C.elegans        179 SEIIKVDQAVPGDVLILTKPIGGQVAVNSYEWIKKKNGKIEELN----LEIPKIEKAFKQ
D.pseudoobscura  179 DEIIFPSNGRPGDALVLTKPLGTQLATNAHIWQLNQTDKYKTLL--TKFTDKEILETFEM
D.melanogaster   175 EEIILPSNAQPGDVLVLTKPLGGQMAMDAHLWQLNQTEKYKKLL--SECSDADIKETFEI

H.sapiens        291 AMFNMATLNRTAAGLMHTFNAHAATDITGFGILGHSQNLAKQQRNEVSFVIHNLPIIAKM
D.rerio          276 AMLNMATLNRTAAALMHKFNAHAATDITGFGIIGHARNLAKQ------------------
D.discoideum     219 ATLSMARLNRVGARLMKKYNAHAATDVTGFGILGHSTNLAQNQLLPIRFEIHTLPIIKHM
T.pseudonana     230 AVCSMARLNRHGGSLMVKHAAHAGTDVTGFGILGHAQNLIENQRLQVGMEIHTLPCIANT
C.elegans        235 VCEQMSRLNRNAAKLLHKYDAHSSTDVTGFGLLGHAENLARVQKQPMEFIIEKLPIIEYM
D.pseudoobscura  237 ALKSMTYLNKTAARLMHKYRAHCATDVTGFGLLGHAKNLAQFQKDKVLFKINRLPIIKNV
D.melanogaster   233 AVKSMTYLNKNAALLMHKYQAHCATDITGFGLLGHANNLAQFQKEKVLFQINKLPIIKNV

H.sapiens        351 AAVSKASGR-----FGLLQGTSAETSGGLLICLPREQAARFCSEIKSSKYGEGHQAWIVG
D.rerio              ------------------------------------------------------------
D.discoideum     279 KKLEDHLNHP----FKLLKGTSAETSGGLLISMSRENAEAFCKEIYEIE---KQPAWIIG
T.pseudonana     290 AKVEKSGVLD----FRLLKGYSAETSGGLMICMSEKDALAYCRELEELD---GEQAWIVG
C.elegans        295 DEIADKMIAKGGEGFKLYQGTSAETSGGLLIAMSEENAKKYIAELSSLD---NAPAWIIG
D.pseudoobscura  297 VKFSTLIDQS----SKLLAGKSVETSGGLLISLSPEAADQFCCEFNEITKG-EQKAFKIG
D.melanogaster   293 LKFSTLVGQS----TKFRSGRSVETSGGLLICLPADAADKFCRDFEEATNG-EQKSFQIG

H.sapiens        406 -IVEKGNRTAR------IIDKPRVIEVLPRGATAAVLAPDSSNASSEPSS
D.rerio              --------------------------------------------------
D.discoideum     332 DVIDQSSNYDRSKNTSIILENPKIIEVEPNTNF-----------------
T.pseudonana     343 RVVADEGRKAR------IVEDVTVLEV-----------------------
C.elegans        352 KVTAKTTDSSIAR----ILPDAVRISVPSHI-------------------
D.pseudoobscura  352 CVEEANESDAV------LADHVEYIEVSL---------------------
D.melanogaster   348 HVTAANESDAV------LCEDVEFIEVSL---------------------
```

Glutathione peroxidase

```
H.sapiens             1 ------------------------------------------------------------
M.musculus            1 ------------------------------------------------------------
D.rerio               1 ------------------------------------------------------------
C.elegans             1 ------------------------------------------------------------
A.thaliana            1 ------------------------------------------------------------
O.sativa              1 ------------------------------------------------------------
C.reinhardtii         1 -----------------------------------------------------------M
O.lucimarinus GpxA    1 ------------------------------------------------------------
O.tauri GpxA          1 ------------------------------------------------------------
O.lucimarinus GpxB    1 ------------------------------------------------------MPALTS
O.tauri GpxB          1 ------------------------------------------------------------
O.lucimarinus GpxC    1 ------------------------------------------------------------
O.tauri GpxC          1 ------------------------------------------------------------
O.lucimarinus GpxD    1 MRTRHSVTRGGAPARRFMSTTAARAGATRTMTTTRATTRRARATATTTRATNGRGVAVTV
O.tauri GpxD          1 ------------------------------------------------------------
O.lucimarinus GpxE    1 -----------------MPPDVESSGTAPFAAAYPGTARANRARHGPLVLILLTTVLGGF
O.tauri GpxE          1 ------------------------------------------------------------
T.pseudonana Gpx-a    1 ------------------------------------------------------------
T.pseudonana Gpx-b    1 ------------------------------------------------------------

H.sapiens             1 -------------------------MCAARLAA---AAAQSVYAFSAR---PLAGG----
M.musculus            1 -------------------------MCAARLSA---AAQSTVYAFSAR---PLTGG----
D.rerio               1 --------------------------------------------LSAK---LLSG-----
C.elegans             1 --------------------------------------MSSVYDFNVK---NANG-----
A.thaliana            1 --------------------------------MAASSEPKSLYDFTVK---DAKG-----
O.sativa              1 --------------------------------MAAAPSATSVHDFTVKGVQDASG-----
C.reinhardtii         2 LLTRKNVAVRPARAARRDVRAMSLLGNLFGGGSKPTSSTSNFHQLSAL---DIDK-----
O.lucimarinus GpxA    1 --MLASRHALANTAFARAPARPRAARASARASVR-VRAASTIYDFTLK---TLGGGARD-
O.tauri GpxA          1 --MLTQRAVVAPARLARARSRTRGLARGARAQT---NASQTIHDFTLK---TLGG-----
O.lucimarinus GpxB    7 TARAATHAFATHRGVNSRRNPRSMQAVRAFFGG-AATSSKSAHEFTVK---DIDG-----
O.tauri GpxB          1 -----MDA--V---------------VRAFFGGGAPKNTKSAHGFTVK---TIDG-----
O.lucimarinus GpxC    1 -------MTRALGAFARSFAP-ARVARSKRARGRVTRAMAAFHDLRAP---ALDG-----
O.tauri GpxC          1 ----PQQHVRGERALVRLVHHHHRILWRGGARATGVERFDARDDGRDA---TIAR-----
O.lucimarinus GpxD   61 DDAYDDDATTTRRRAMARVATLAAMVTVTPARAA--ESPESLYDLTVM---QYGK-----
O.tauri GpxD          1 ---------------MRIVALAGALETTARGRAR--PDVESLYDLTVT---QYGK-----
O.lucimarinus GpxE   44 FLHVVVRPFTDARAHARALQTASFVSADALAARDALGVFEAVLSGVLH---DIDGGVVRD
O.tauri GpxE          1 -----------------------MVDVD---ARRAMHLLETIENGLLR---DIDGGAV--
T.pseudonana Gpx-a    1 ---------------------------------------SAFYDLVDK---DMDG-----
T.pseudonana Gpx-b    1 ----------------------------------------SIYRSNIM---DIHGNQQ--

                                                   *
H.sapiens            26 ------EPVSLGSLRGKVLLIENVASLUGTTVRDYTQMNELQRRLGPRGLVVLGFPCNQF
M.musculus           26 ------EPVSLGSLRGKVLLIENVASLUGTTIRDYTEMNDLQKRLGPRGLVVLGFPCNQF
D.rerio               9 ------DLLNFSSLKGKVVLIENVASLUGTTVRDYTQMNELHSRYADQGLVVLGAPCNQF
C.elegans            15 ------DDVSLSDYKGKVLIIVNVASQCGLTNKNYTQLKELLDVYKKDGLEVLAFPCNQF
C.reinhardtii        54 ------KNVDFKSLNNRVVLVVNVASKUGLTAANYKEFATLLGKYPATDLTIVAFPCNQF
A.thaliana           21 ------NDVDLSIYKGKVLLIVNVASQCGLTNSNYTELAQLYEKYKGHGFEILAFPCNQF
O.sativa             24 ------KDVNLSTYKGKVLLIVNVASQCGLTNSNYTELSQLYEKYKVQGFEILAFPCNQF
O.lucimarinus GpxA   54 -APTDGEDLPLSRYKGKVVLINNVATFUGTTVRDFVQLNALKEKYG-DDLVVLGVPCNQF
O.tauri GpxA         48 -----GADLPLSQYKGKVVLINNVATFUGTTVRDFVQLNALAEKYG-DDLVILGVPCNQF
O.lucimarinus GpxB   58 ------KDVSMSKYAGKVCLVVNVASEUGLTDQNYKELVALDAKY--ANLEVLAFPSNEF
O.tauri GpxB         31 ------ESVELSKYAGKVCLVVNVASQUGLTDQNYKELVQLDKKY--DDLEVLAFPSNEF
O.lucimarinus GpxC   45 ------STIEFASMKGKVVLVTNVASRUGATKREYAALRDLSNKYSP-DLVVVLTPSGQF
O.tauri GpxC         49 ------R--GRWTDDAKVIYAVNVASRUGATGREYAAMKRFEERYSARGLTIVLFPCGQF
O.lucimarinus GpxD  111 ------PT-PLAAYRGKVSVVVNVASEUPLVNSNYPALRRMYETYRDRGFEIIAFPANQF
O.tauri GpxD         36 ------PR-SLGEYKGKVVVVANVASEUPLQNANYPALRSMLEKYRSRGLEFIAFPVNQF
O.lucimarinus GpxE  101 EALLGFPVAKRESSRGAFTLVTNVASHUGYKQSNVEGFRALREEFGDG-LRILGFPSDDF
O.tauri GpxE         30 ----GFGAQS-----GKLRLITNVASHUGYKKANVDGFKRLHDEFGDR-LAILGFPTDDF
T.pseudonana Gpx-a   14 ------NEVPMSSFKGDVLCVVNVASKUGLTKVNYVQFSKLYDEYNSRGFKVLAFPCNQF
T.pseudonana Gpx-b   16 ------QLMK---YAGSISLIVNVACEUGLTKSNYKELAVLYDKYHKRGLNVLAFPSNDF
```

```
H.sapiens                 80 GHQENAKNEEILNSLKYVRPGGGFEPNFMLFEKCEVN-----------------------
M.musculus                80 GHQENGKNEEILNSLKYVRPGGGFEPNFTLFEKCEVN-----------------------
D.rerio                   63 GHQENCKNEEILQSLKYVRPGNGFEPKFQILEKLEVN-----------------------
C.elegans                 69 AGQEPSCEIDIQ---AFVADKFKFEPT--LFQKIDVN-----------------------
A.thaliana                75 GNQEPGTNEEIV---QFACTRFKAEYP--IFDKVDVN-----------------------
O.sativa                  78 GGQEPGSNEEIV---QFACTRFKAEYP--IFDKVDVN-----------------------
C.reinhardtii            108 GGQEPGTNAEIK---AFASARGFSGAGALLMDKVDVN-----------------------
O.lucimarinus GpxA      112 GHQCYDKDFELLNTLKYVRPGDGYEPKFTITGKMTIN-----------------------
O.tauri GpxA            102 GHQCYDKDFELLNTLKYVRPGNGYEPKFQITGKMTIN-----------------------
O.lucimarinus GpxB      110 GGQEPGSAAQIK---AFAREKYGAKFP--LFEKTTVN-----------------------
O.tauri GpxB             83 GGQEPGSAAQIK---EFA-KKYGATFP--MFEKTMVN-----------------------
O.lucimarinus GpxC       98 GGQELAKAEDIA---AFVAKQEFETAPT-VTAKTNVN-----------------------
O.tauri GpxC            101 GGQELAKDADIL---KFVADKGLTKAR--VAAKGDIQ-----------------------
O.lucimarinus GpxD      164 GCQAPG-TSEME---RAYAYKKFGVDTFPVMDKIAVKAKPVRCKGIDPASYEEFKDDPDA
O.tauri GpxD             89 GCQGPG-TSEME---REYAYKKFGTRDFEVMDKIA-------------S-YE---D----
O.lucimarinus GpxE      160 GHQQGT-NEEIK---RDFA-----DVMDVLFEPALD------------------------
O.tauri GpxE             80 GHQMGS-QEELR---HDFGS---KEVVDILFEPTR-------------------------
T.pseudonana Gpx-a       68 GAQEPGTPAEIL---EFVEKNFQAKDKFTWFEKGHVN-----------------------
T.pseudonana Gpx-b       67 HQEKET-NEEIL---EYVRSN-FPEVRFPLFSRAP-------------------------

O.lucimarinus GpxA  149 ------------GDEEDPFWTFLKNSIREPADDKGGLGSDHIYKTQPNSMPIQWSPVRRA
O.tauri GpxA            139 ------------GEDEDAFWTFLKRAIPYPADD------DFIYNTQPNSMPLQWSPVRRS
H.sapiens                117 ------------GAGAHPLFAFLREALPAPSDD------ATALMTDPK--LITWSPVCRN
M.musculus               117 ------------GEKAHPLFTFLRNALPTPSDD------PTALMTDPK--YIIWSPVCRN
D.rerio                  100 ------------GENAHPLFAFLKEKLPQPSDD------PVSLMGDPK--FIIWSPVCRN
C.elegans                101 ------------GDKQSPLFKFLKNEK--GGFM-----------FDA-------------
A.thaliana               107 ------------GDKAAPVYKFLKSSK--GGLF-----------GDG-------------
O.sativa                 110 ------------GNNAAPLYKYLKSNK--GGLF-----------GDS-------------
C.reinhardtii            142 ------------GANASPVYNFLKVAA-----------------GDTS------------
O.lucimarinus GpxB      142 ------------GASASPLYKHLKESAPESGLL-----------AMAGS-----------
O.tauri GpxB            114 ------------GPSANPLWKHLKETAPESGLM-----------ALAGS-----------
O.lucimarinus GpxC      131 ------------GADASPVWTFLKKAS-----------------GDES------------
O.tauri GpxC            133 ------------GANANSAWRALKEAS-----------------GDVS------------
O.lucimarinus GpxD      220 VLTAPLVDDVTSSAKQSPVYEFLKRPP-----------------FDG-------------
O.tauri GpxD            124 ------------SAKESPVYEFLKRKP-----------------FDK-------------
O.lucimarinus GpxE      187 -------------LAHNPMFAQLTAGG------------------AP-------------
O.tauri GpxE            108 -------------LRDNPIFGELATS-------------------AP-------------
T.pseudonana Gpx-a      102 ------------GKDTREVYSFLKAKLP----S-----------EDGTS-----------
T.pseudonana Gpx-b       97 -------------LASNLVFQLCEKHT-----------------GES-------------

O.lucimarinus GpxA      197 DVTWNFE-KFLIGKDGVPAKRYSP-KFENANLTADIDALMKA------------
O.tauri GpxA            181 DVVWNFE-KFLIGKDGKPAKRYSP-KFENANLTADIDALIKA------------
H.sapiens                157 DVAWNFE-KFLVGPDGVPLRRYSR-RFQTIDIEPDIEALLSQGPSCA-------
M.musculus               157 DIAWNFE-KFLVGPDGVPVRRYSR-RFRTIDIEPDIETLLSQQSGNS-------
D.rerio                  140 DISWNFE-KFLIGPDGEPFKRYSR-RFLTIDIDADIKELLKRTK----------
C.elegans                123 -IKWNFT-KFLVGRDGKIIKRFGP-TTDPKDMEKDIKEALGEKL----------
A.thaliana               129 -IKWNFA-KFLVDKDGNVVDRFAP-TTSPLSIEKDVKKLLGVTA----------
O.sativa                 132 -IKWNFS-KFLVDKEGRVVDRYAP-TTSPLSIEKDIKKLLGSS-----------
C.reinhardtii            161 DIGWNFG-KFLVRPDGTVFGRYAP-TTGPLSLEKYIVELINSR-----------
O.lucimarinus GpxB      168 EIKWNFA-KFLLDKDGKTIARYAP-TSSPFSIEGDIIKAL--------------
O.tauri GpxB            140 EIKWNFA-KFLLDKDGKTVGRYAP-TSSPLSIESDILKYL--------------
O.lucimarinus GpxC      150 DVRWNFACKFLVDRDGNVVERNG---DGAADNEAKIVKLLG-------------
O.tauri GpxC            152 DTRWNFSTKFLVSRDG-VVERRE---EGADALEERIVQLLDA------------
O.lucimarinus GpxD      250 EIPWNYT-KFLVGRDGRVLRRYGPGDPLEQGFEEDLKRALDGVALTAKPRNPYL
O.tauri GpxD            142 EIEWNYV-KFLVGRDGQVLRRYSPGDPLEQGMEEDIKRALDGREL-GKPRNPYA
O.lucimarinus GpxE      203 -AEWNFA-KFLVDDRGRVVRRYPP-KMDAQAIARDIRDEL--------------
O.tauri GpxE            123 -VEWNFV-KFLVDDTGRVVQRYPA-GFDYDKIAADVRDELRRR-----------
T.pseudonana Gpx-a      124 DIRWNFA-KFMVDHEGNPFKRYGP-KTNPEDMKSDIEELLAK------------
T.pseudonana Gpx-b      114 -VAWNFH-KYLVDGEGRAVKSYGH-RVQPMDMEGDIVRLLEE------------
```

MSP

```
O.tauri           1 ----------------------------------------------MTR--ARRGVKDSN
O.lucimarinus     1 ----------------------------------------------MS----------SQ
D.discoideum      1 --MSLFNLPKVDLGEDCEGGVUARPDESTPLISKTNDEEKANIGISSTSNSPQEEQTKKP
C.reinhardtii     1 -----------------------MPPLAVRRRLAEQAAAQADADEVASELSDDELPDQKS
V.carteri         1 -----------------------MPPLASRRRLDAAPAAP----EAVS--PSGEAADKRS

                                      *
O.tauri          13 RVAVACAIAVSVPSLVGSUCWPLLAASLTGAAAS-AGAKRASHALSLGVNLLATGAVAIA
O.lucimarinus     5 RVLTAMAVLASAPSLVGAUCWPLLVGSFFGAAATSASAKHESHAISLGMNAMVTTLATVA
D.discoideum     59 LFISILTLLISIPALVGSUCWPVLIASLSGVAVS-AGSVELAHSLTFAITLSILSNLAQY
C.reinhardtii    38 LASSIAALLLVLPALVGSUCWPLLLAGFMGITAT-AGAKAFSHTFSLALTAVILTNLAQY
V.carteri        32 LAASVAALLLALPALVGSUCWPLLLAGFMGITAT-AGAKAFSHTFSLALTLAILTNLAQY

O.tauri          72 TARRAKTRSGT--YWHRKGPMILTMASVFLLCADPVRHVLQDEDVWTTG----SSMYRDD
O.lucimarinus    65 SARKARGRRGN--AWRVYGPTATTAASVALLCADPMRHVLQDHELWTTN----SAMYRPG
D.discoideum    118 HFHKCKKRPSDRGHWIKFGPFYLTAIAVPLATFDILRHILVDNSIWTIHSFISPAAYRPG
C.reinhardtii    97 TAWKGLSHGGT--HWHRYGPAYLLVVATPLLLADLTRHSLQDAGVWTGP---SSRMYRDD
V.carteri        91 TAWKSTSRSGS--HWQRYGPAWLLVIATPXMCADLVRHCLQ-------------------

O.tauri         126 CEHAD----VRCLSAVGWI-FTLCTYFGFACLIAGSAWNADAFGKIGREWNRRRHAGGDS
O.lucimarinus   119 CEHGD----IRCLSVVGWV-FLTCTYLGFACLIVGALWNADALGKLGREFRRR-------
D.discoideum    178 CENEN----ITCLSVMGWFSAIVFTYTGYACLLVGTIWAADLIPKIKKVWTQLR------
C.reinhardtii   152 CSPVTGLHGFYCLSLTGWVFSIFCTYTGFVLMIVSVFWSSKIMHKLRHAWHHIH------
V.carteri           ------------------------------------------------------------

O.tauri         181 SNAGDDADGCDEP
O.lucimarinus   167 -LEGDDAD-FEA-
D.discoideum    228 -PSKKN-------
C.reinhardtii   206 -LARR--------
V.carteri           -------------
```

>*O. tauri* hypothetical protein 1
MGYAGYAPGHRVAEQKTPLERALDSVRNHETLDVVEKLTRNVCRDPNDEKYRRIRSSSATIKRLVFDDENALSV
MLALGWRFEEGEREMTLSRDARTTMADVRAIDAARTSLRRRLEEEMHARIRERARAKDPAVQALREQMEADRGE
RSAREPVTEGSRAVERTSGRVVTASDVGASGSSGC**U**G

>*O. lucimarinus* hypothetical protein 1
MAYAGYAPGHRVVERTPLERALRAIARPETLDLLERLSKNVARAPSEAKYRKLKRANATIGREIFDDERAMRAM
MTMGWIAETIEGSECLTLPRGTTTTMREVRAMDEARTALRRRLEEEMRAKIRARAMANDPRRAALREAVEADRA
ERAVREPVTTGSRAAARGTGTTVTAKEVGASGSSGC**U**G

>*O. tauri* hypothetical protein 2
MARDVGDLIDVDASECLNATDPRAWTSVLARGTLGEPAGALASDDDHELLIRLVFVVPVRARALVIRSPTTRAD
ADVSGVGTVRVFVNGQAMGFENVARRKPAQTLEGAGEHALDATAFDRVRALTIFVESNDKGTARTVISEIRVFG
DALASTDVGALKPC**U**GR

>*O. lucimarinus* hypothetical protein 2
MLDLIDHVDERSVECLNALTDESWRNALWPGPRDRASSTLVSDDDEELILRVEFTSNVRPRAVKIAGASATHAR
EDASAPRVVKIFVNAPSLSFENAAKRRAAQVVELDGDDEVELDVTAFENVRVMTFYVESNVGGTARTEIGRIDI
KGELSGELRDVSELKPC**U**GR

>*O. tauri* hypothetical protein 3
MYAPGTGKSIYFGVFQRDVRAEAGRETPSDDVRAARRARAREELVNIDDEERARRLDVGKVAGGVTLVMAIAQL
ALGASRIERAVIAIPLFFALGFVGSAKSGL**U**NIAQAGVWDVEGAGLQKIEDETIAQKIRDKVNDFNAKSAIIVF
AITAAYCALPLR

>*O. lucimarinus* hypothetical protein 3
MRPSFATPRAATPRAATPRAARATRATPRRAATPTRATRDDRARDLFANLYRGGGGKQIYYGVFQRDAPASATK
SEEARARSRDEAAKTLTNIDAEERGRRMEVGKAAGAATAALAAAQLALGATRVERLAIAVPLFFALGFVGSAKT
GL**U**NIAQAGVWDVDGTGLQDIEDESVATAIRKKVNDFNGKSAAAVAVITAMYVALPV

>*O. lucimarinus* Prx-like protein
MRAARAPTVAPAPVAARRARAARRVAPRALDCGEIACAVDDDVGARTATINGASVSAAALRACEIVGADGTRAP
LGRATSGAPNVVVVLRHLA**U**LFCWSYARSVADSRGRIEAAGGSVTLVSLGTAEQLRTFLELNPEIPSDIAFVDD
SDDFALYDACGFGKFTDAKPEKVDLKPTNFSFKDWLAYLGNAGKLAPIKKGQKGVPEGVLRLGGTFVLAGDDVE
YAWADALPGAHPEIADVLKAVGI
