# Supplementary material for: Evolutionary dynamics of eukaryotic selenoproteomes: large selenoproteomes may associate with aquatic life and small with terrestrial life
Source: Genome Biol. 2007 Sep 19;8(9):R198. doi: 10.1186/gb-2007-8-9-r198 (PMC2375036; doi:10.1186/gb-2007-8-9-r198)
Supplement: Additional data file 3 — Sequence and predicted clover-leaf structure of T. pseudonana Sec tRNA. [file gb-2007-8-9-r198-S3.pdf]

Sequence and predicted clover-leaf structure of *T. pseudonana* Sec tRNA

GUGUGAAUGAUCCUGCCUGGUGGUGGGUUCAGGCU**UCA**AACCUGAAGGGGCUUAGCGGCCCAGUGGUUCGAUUCCACCUUUCGCACGCCA

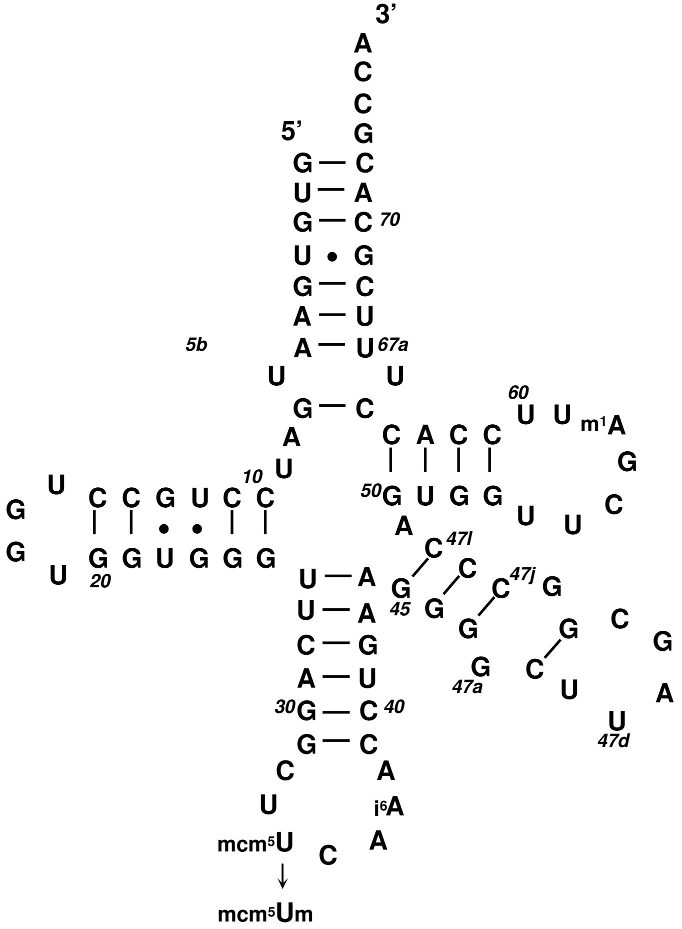

9/4

Structure of *Thalassiosira pseudonana* selenocysteine tRNA
