# Supplementary material for: Evolutionary dynamics of eukaryotic selenoproteomes: large selenoproteomes may associate with aquatic life and small with terrestrial life
Source: Genome Biol. 2007 Sep 19;8(9):R198. doi: 10.1186/gb-2007-8-9-r198 (PMC2375036; doi:10.1186/gb-2007-8-9-r198)
Supplement: Additional data file 4 — Representative phylogenetic trees of selenoproteins. [file gb-2007-8-9-r198-S4.pdf]

Additional data file 4

Each tree corresponds to one selenoprotein, which is shown above the tree. In all figures in this data file, selenoproteins are indicated by labeling organisms in red, and cysteine-containing homologs in blue. If a different selenoprotein (distant homolog) is included in the tree, the name of this protein is indicated in addition to the name of an organism from which this protein is derived. If organism name is indicated more than once in the same tree, this organism has several homologs of the same protein. For reference, bacterial homologs were included in the SelW and DI trees.

SelM

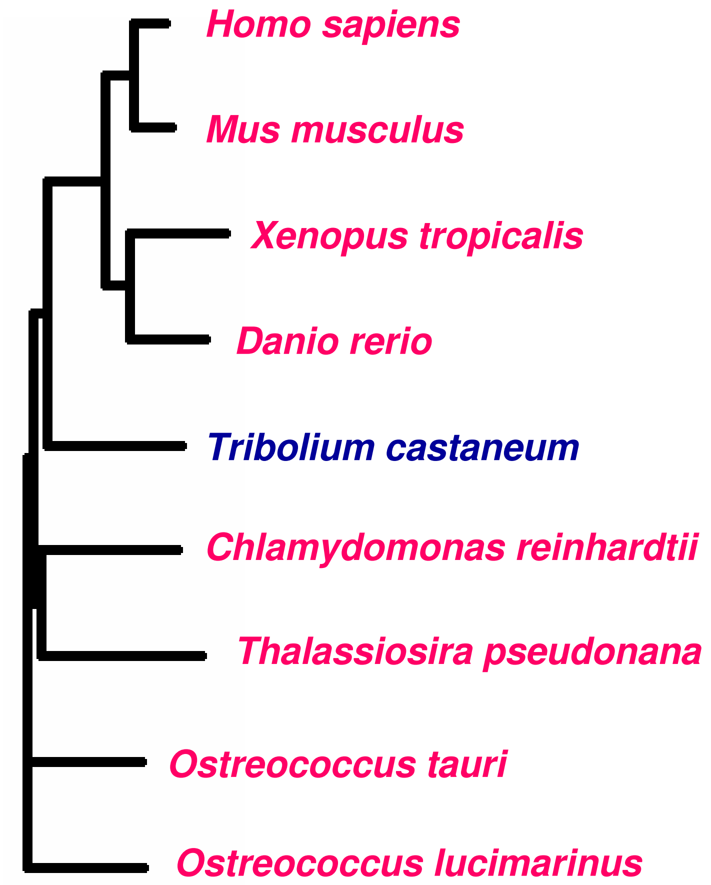

Homo sapiens
Mus musculus
Xenopus tropicalis
Danio rerio
Tribolium castaneum
Chlamydomonas reinhardtii
Thalassiosira pseudonana
Ostreococcus tauri
Ostreococcus lucimarinus

Sep15

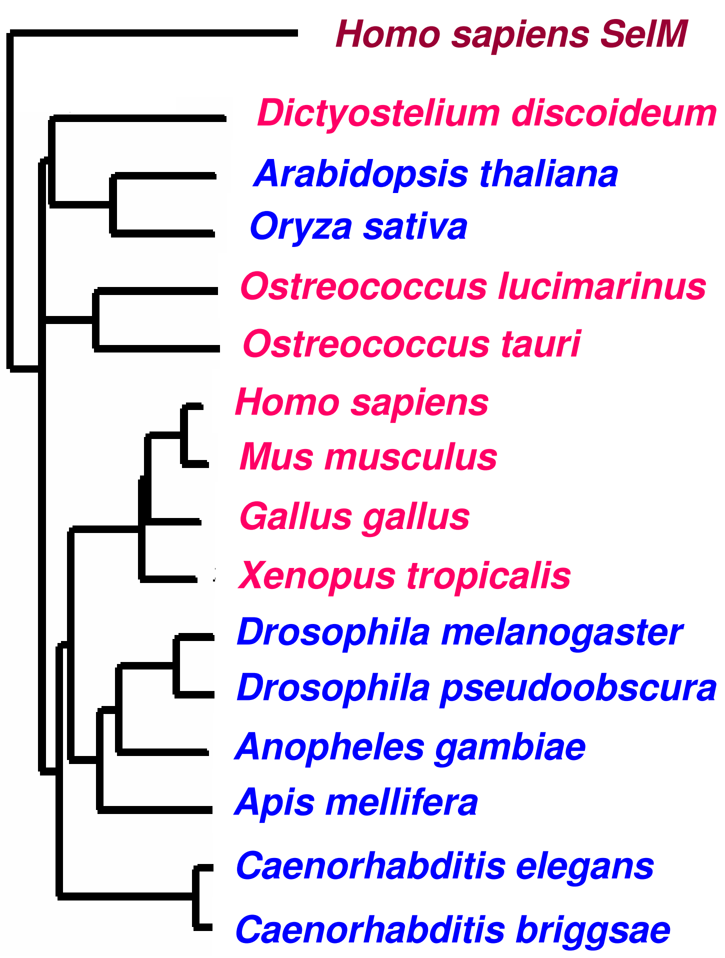
Homo sapiens SelM
Dictyostelium discoideum
Arabidopsis thaliana
Oryza sativa
Ostreococcus lucimarinus
Ostreococcus tauri
Homo sapiens
Mus musculus
Gallus gallus
Xenopus tropicalis
Drosophila melanogaster
Drosophila pseudoobscura
Anopheles gambiae
Apis mellifera
Caenorhabditis elegans
Caenorhabditis briggsae

SelS

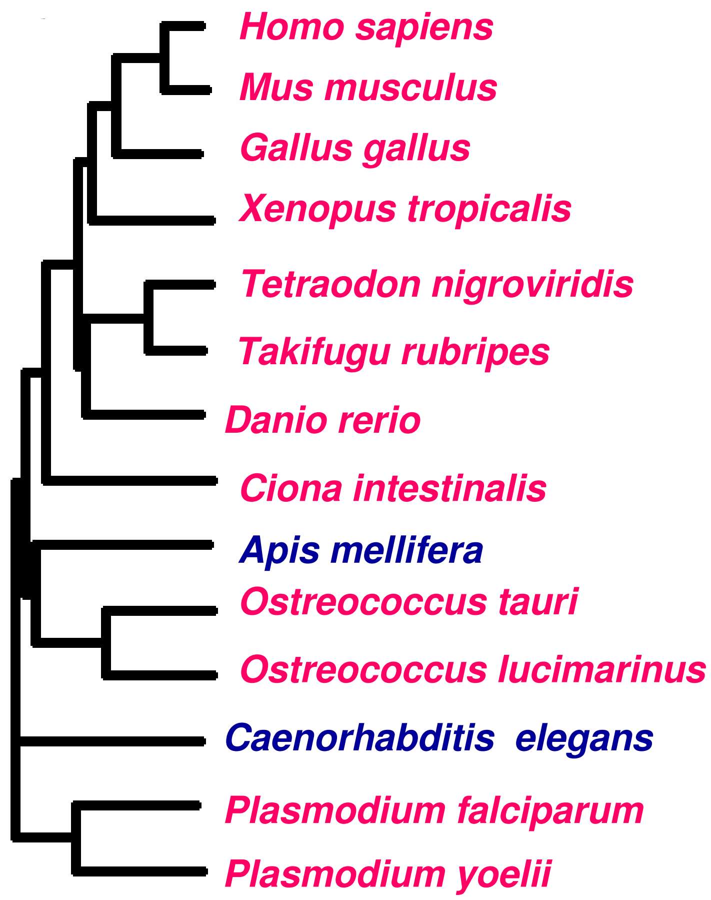
Homo sapiens
Mus musculus
Gallus gallus
Xenopus tropicalis
Tetraodon nigroviridis
Takifugu rubripes
Danio rerio
Ciona intestinalis
Apis mellifera
Ostreococcus tauri
Ostreococcus lucimarinus
Caenorhabditis elegans
Plasmodium falciparum
Plasmodium yoelii

SelK

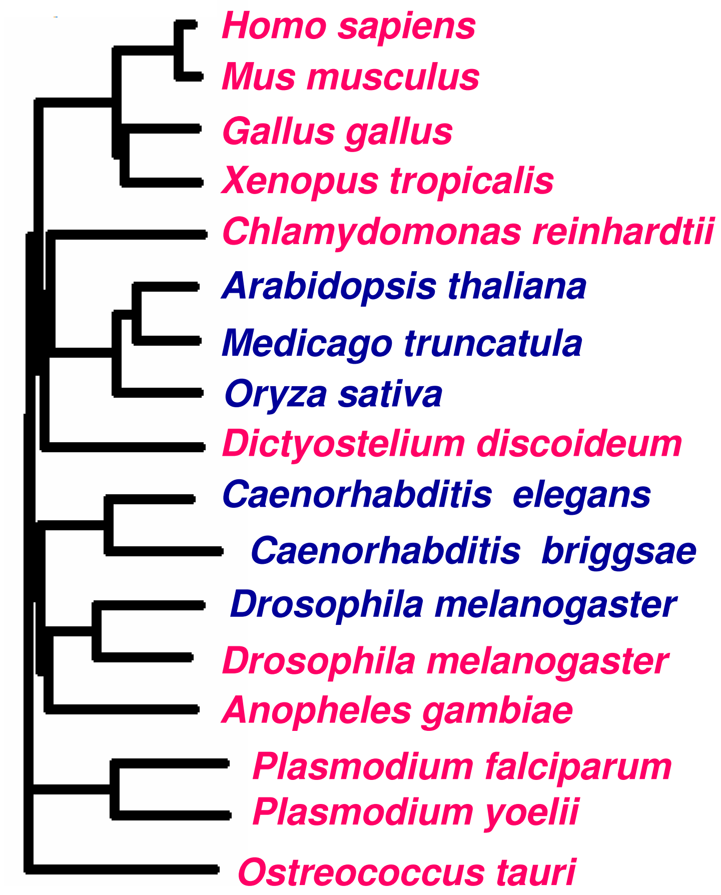
Homo sapiens
Mus musculus
Gallus gallus
Xenopus tropicalis
Chlamydomonas reinhardtii
Arabidopsis thaliana
Medicago truncatula
Oryza sativa
Dictyostelium discoideum
Caenorhabditis elegans
Caenorhabditis briggsae
Drosophila melanogaster
Drosophila melanogaster
Anopheles gambiae
Plasmodium falciparum
Plasmodium yoelii
Ostreococcus tauri

SelW

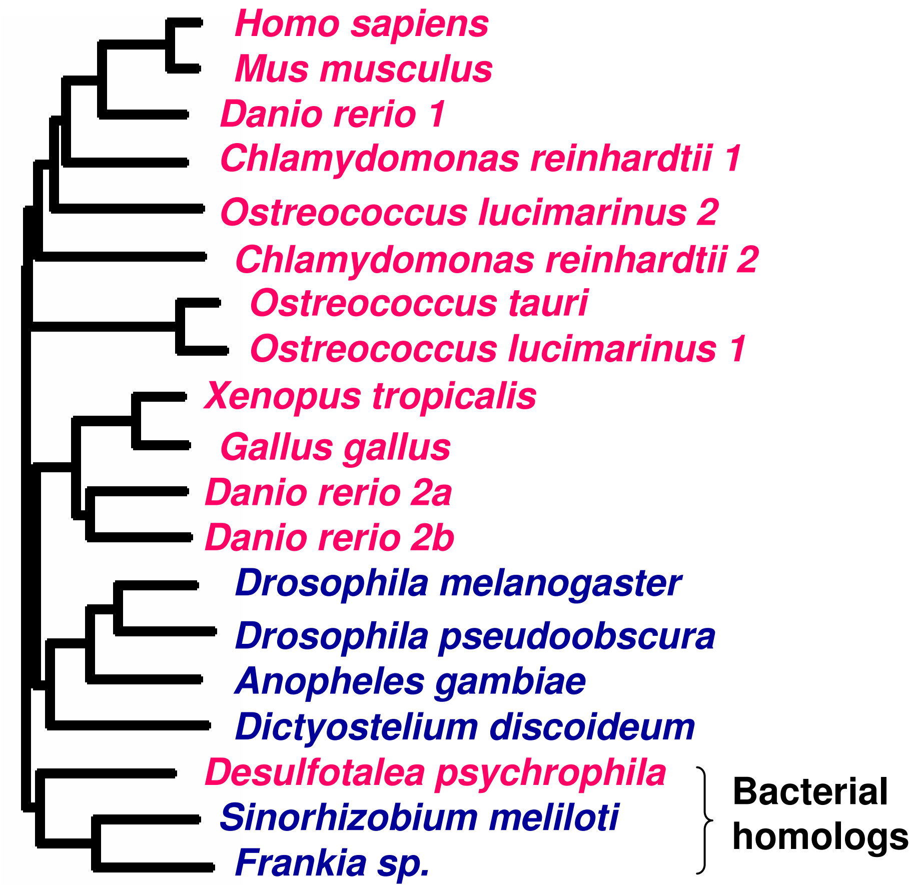
Homo sapiens
Mus musculus
Danio rerio 1
Chlamydomonas reinhardtii 1
Ostreococcus lucimarinus 2
Chlamydomonas reinhardtii 2
Ostreococcus tauri
Ostreococcus lucimarinus 1
Xenopus tropicalis
Gallus gallus
Danio rerio 2a
Danio rerio 2b
Drosophila melanogaster
Drosophila pseudoobscura
Anopheles gambiae
Dictyostelium discoideum
Desulfotalea psychrophila
Sinorhizobium meliloti
Frankia sp.
Bacterial homologs

SelN

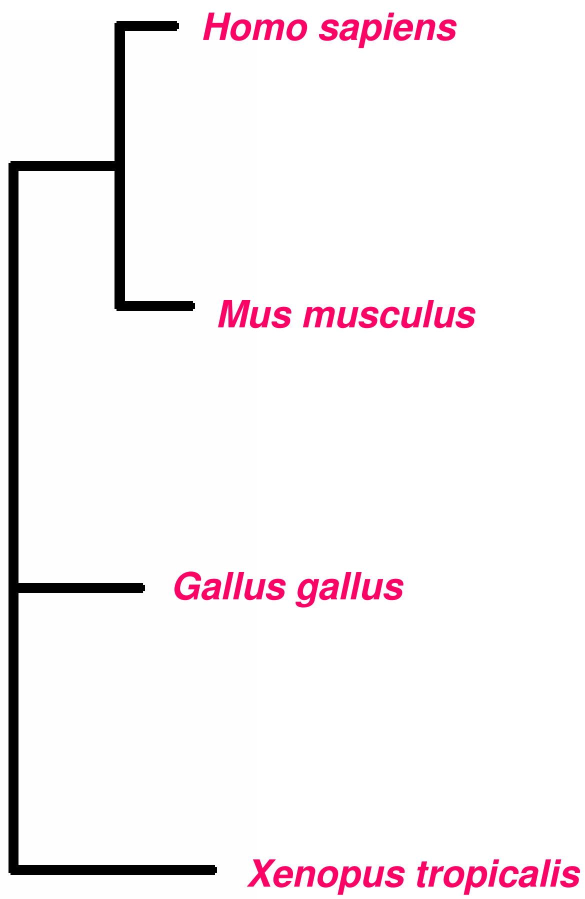
Homo sapiens
Mus musculus
Gallus gallus
Xenopus tropicalis

DI

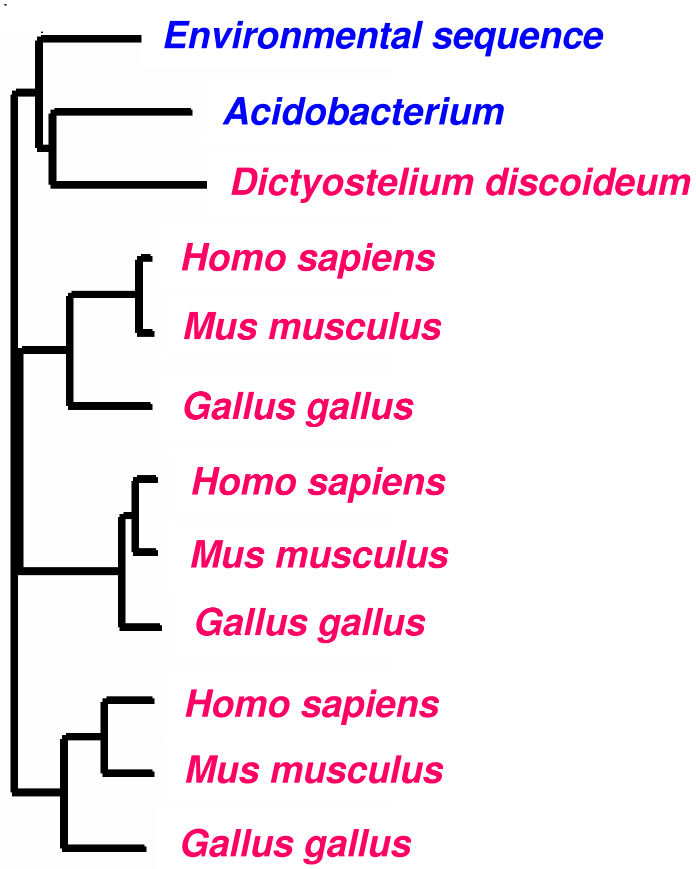
Environmental sequence
Acidobacterium
Dictyostelium discoideum
Homo sapiens
Mus musculus
Gallus gallus
Homo sapiens
Mus musculus
Gallus gallus
Homo sapiens
Mus musculus
Gallus gallus

SelT

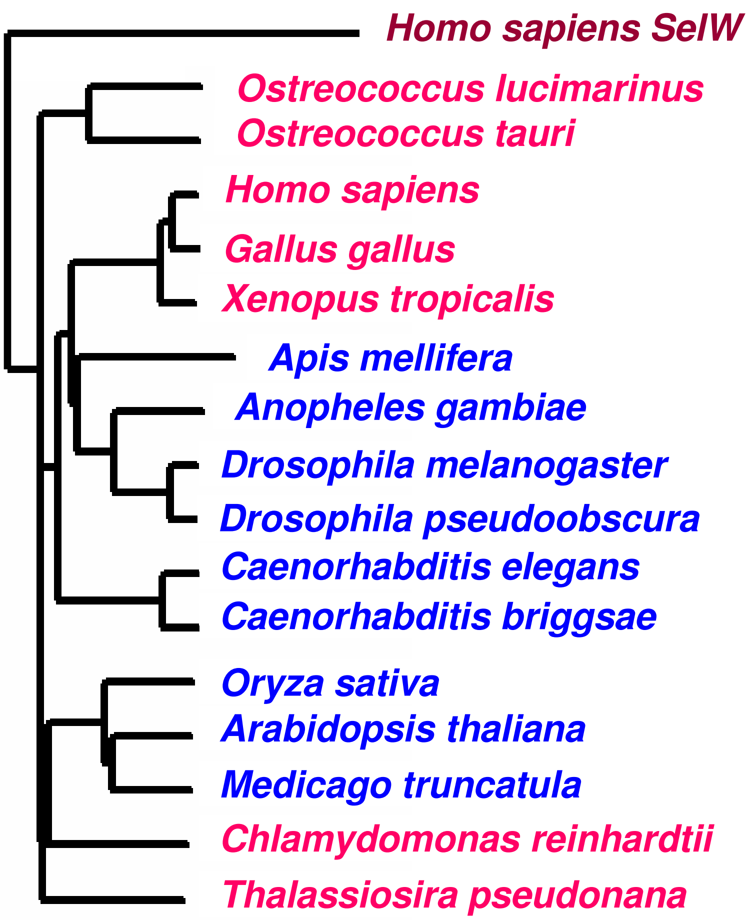
Homo sapiens SelW
Ostreococcus lucimarinus
Ostreococcus tauri
Homo sapiens
Gallus gallus
Xenopus tropicalis
Apis mellifera
Anopheles gambiae
Drosophila melanogaster
Drosophila pseudoobscura
Caenorhabditis elegans
Caenorhabditis briggsae
Oryza sativa
Arabidopsis thaliana
Medicago truncatula
Chlamydomonas reinhardtii
Thalassiosira pseudonana

SelH

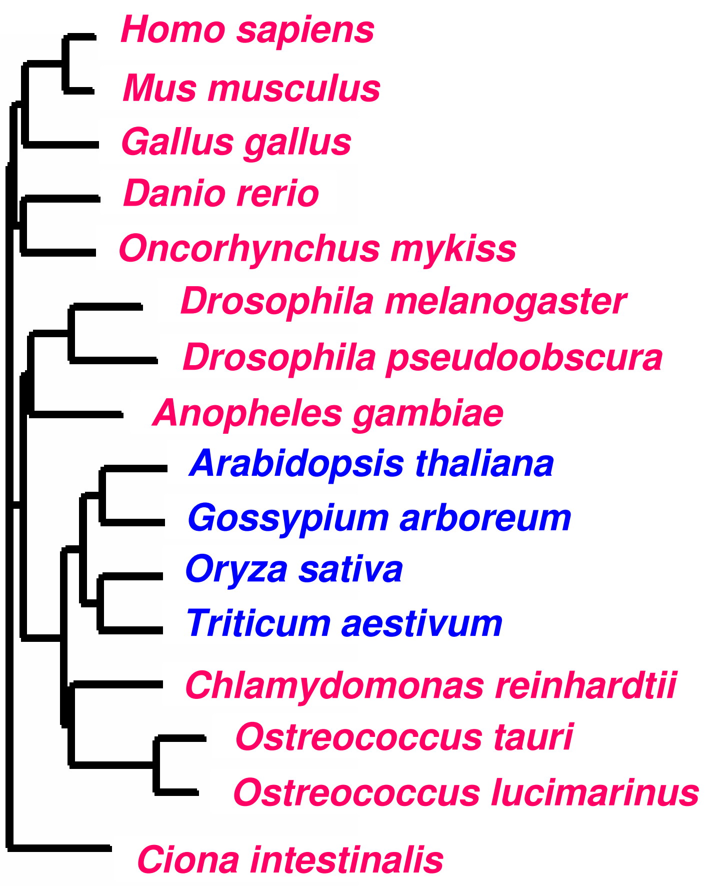
Homo sapiens
Mus musculus
Gallus gallus
Danio rerio
Oncorhynchus mykiss
Drosophila melanogaster
Drosophila pseudoobscura
Anopheles gambiae
Arabidopsis thaliana
Gossypium arboreum
Oryza sativa
Triticum aestivum
Chlamydomonas reinhardtii
Ostreococcus tauri
Ostreococcus lucimarinus
Ciona intestinalis

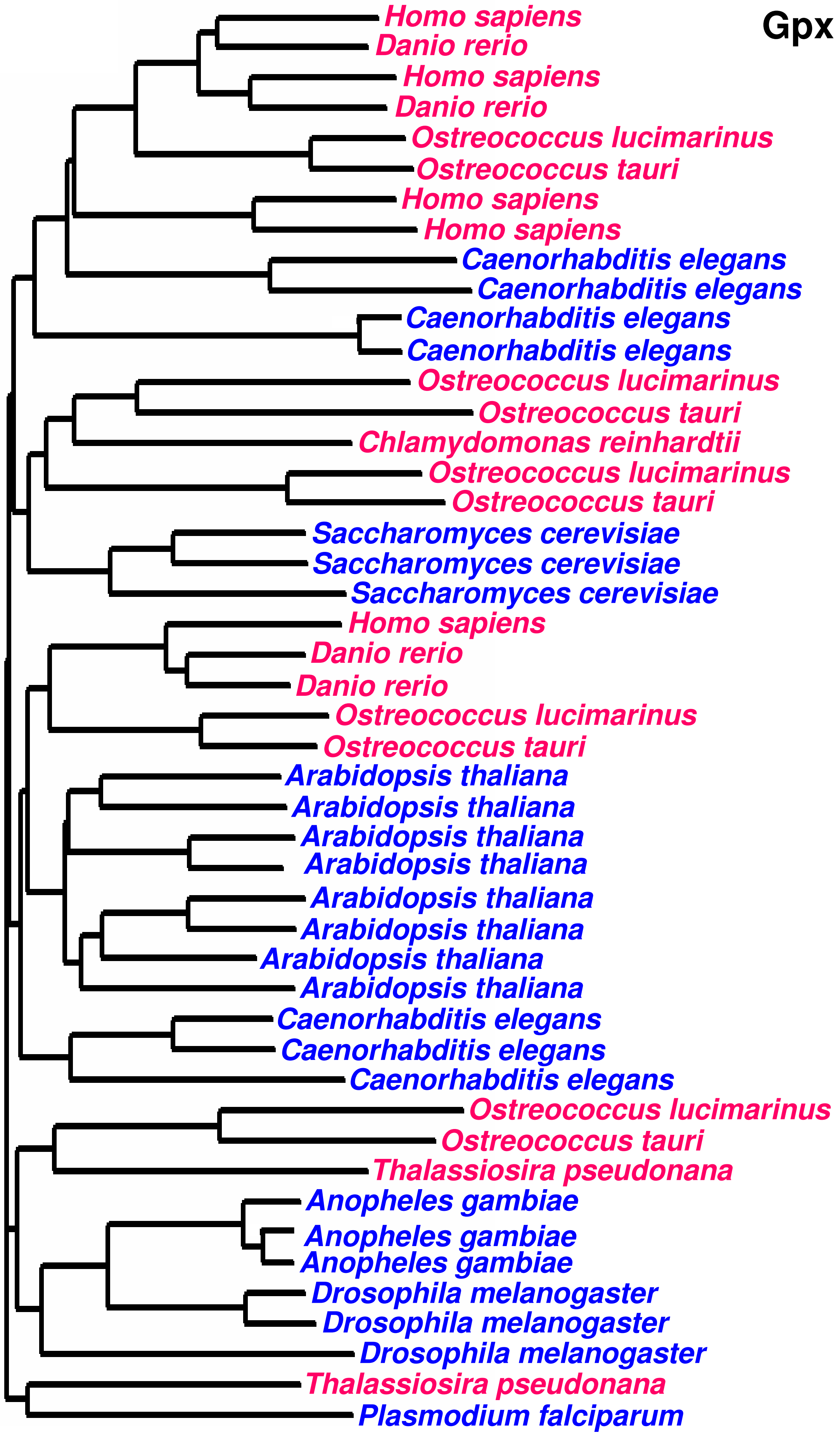
Gpx
Homo sapiens
Danio rerio
Homo sapiens
Danio rerio
Ostreococcus lucimarinus
Ostreococcus tauri
Homo sapiens
Homo sapiens
Caenorhabditis elegans
Caenorhabditis elegans
Caenorhabditis elegans
Caenorhabditis elegans
Ostreococcus lucimarinus
Ostreococcus tauri
Chlamydomonas reinhardtii
Ostreococcus lucimarinus
Ostreococcus tauri
Saccharomyces cerevisiae
Saccharomyces cerevisiae
Saccharomyces cerevisiae
Homo sapiens
Danio rerio
Danio rerio
Ostreococcus lucimarinus
Ostreococcus tauri
Arabidopsis thaliana
Arabidopsis thaliana
Arabidopsis thaliana
Arabidopsis thaliana
Arabidopsis thaliana
Arabidopsis thaliana
Arabidopsis thaliana
Arabidopsis thaliana
Caenorhabditis elegans
Caenorhabditis elegans
Caenorhabditis elegans
Ostreococcus lucimarinus
Ostreococcus tauri
Thalassiosira pseudonana
Anopheles gambiae
Anopheles gambiae
Anopheles gambiae
Drosophila melanogaster
Drosophila melanogaster
Drosophila melanogaster
Thalassiosira pseudonana
Plasmodium falciparum

SelU

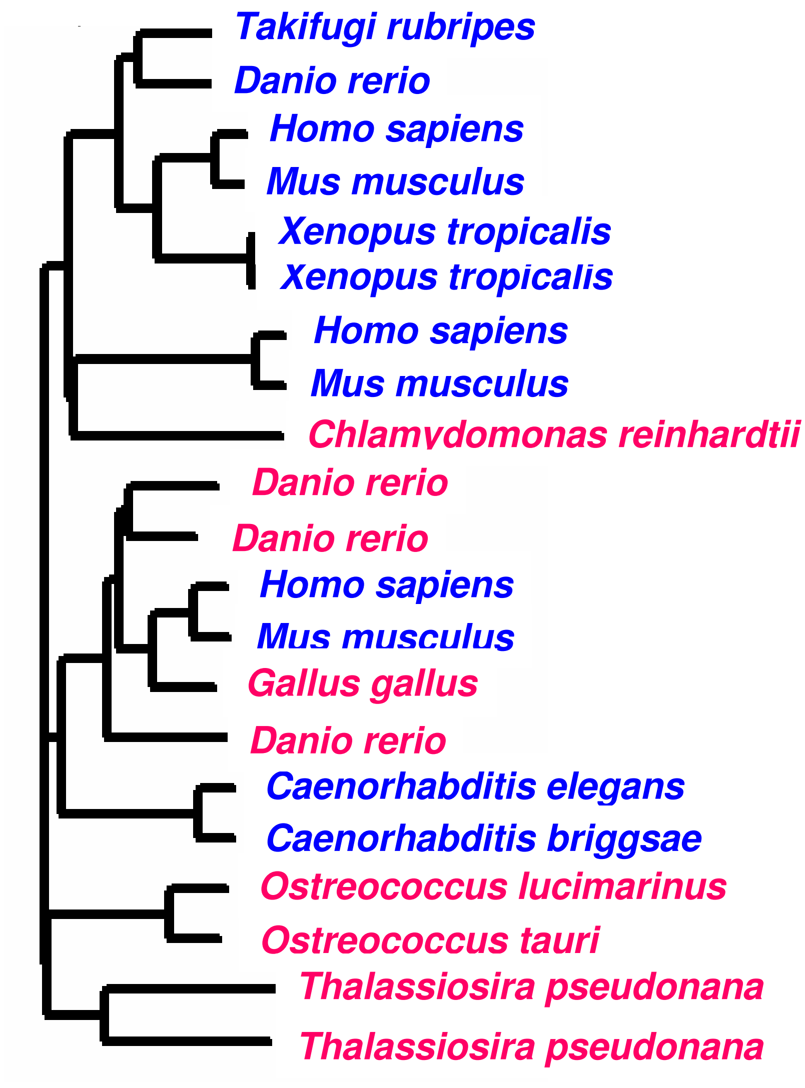
Takifugi rubripes
Danio rerio
Homo sapiens
Mus musculus
Xenopus tropicalis
Xenopus tropicalis
Homo sapiens
Mus musculus
Chlamydomonas reinhardtii
Danio rerio
Danio rerio
Homo sapiens
Mus musculus
Gallus gallus
Danio rerio
Caenorhabditis elegans
Caenorhabditis briggsae
Ostreococcus lucimarinus
Ostreococcus tauri
Thalassiosira pseudonana
Thalassiosira pseudonana
